# Should I Stay or Should I Go? Applying a Combined Experimental and Theoretical Approach to Predict the Excited-State Intramolecular Proton Transfer (ESIPT) in Imidazo[1,5‑*a*]pyridine-3-yl Phenols

**DOI:** 10.1021/prechem.6c00034

**Published:** 2026-05-06

**Authors:** Anita Cinco, Gioele Colombo, Stefano Brenna, Chiara Vola, Bruno Therrien, G. Attilio Ardizzoia

**Affiliations:** † Department of Science and High Technology, University of Insubria and CIRCC, Via Valleggio, 9, 22100 Como, Italy; ‡ Department of Science, Technology and Society, University School for Advanced Studies IUSS, Palazzo del Broletto, Piazza Vittoria 15, 27100 Pavia, Italy; § Institute of Chemistry, 27214 University of Neuchatel, Avenue de Bellevaux 51, CH-2000 Neuchatel, Switzerland

**Keywords:** imidazo[1,5-*a*]pyridine, ESIPT, fluorescence, structure–property relationship, DFT

## Abstract

The fluorescent behavior of a series of imidazo­[1,5-*a*]­pyridine-3-yl phenols was investigated both experimentally (in solution, the solid state, and thin films) and theoretically. Among these compounds, only a subset exhibited excited-state intramolecular proton transfer (ESIPT) emission, identified as a thermodynamically driven process. To understand this behavior, a comprehensive theoretical study was carried out to elucidate the nature of the electronic transitions and to identify the molecular factors governing the occurrence of ESIPT, revealing that fine-tuning the electron-donating and—withdrawing properties is needed for the proton transfer to take place. A structure–property relationship approach was employed, resulting in the development of a numerical descriptor (Ω), dependent on the substituents on the core architecture, capable of predicting whether, upon excitation, the proton “stays” on the oxygen atom or “goes” to the nitrogen atom via the ESIPT mechanism. The same approach also proved to be effective in predicting the frontier orbital energies of the studied systems.

## Graphical abstract

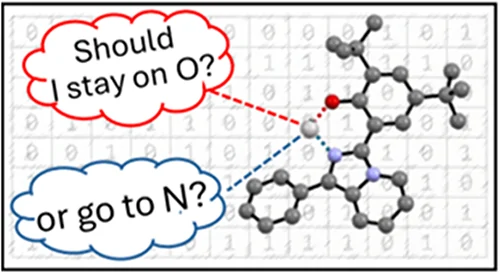

## Introduction

First observed in 1956 by Weller in salicylic acid,^1^ excited-state intramolecular proton transfer (ESIPT) is a prominent photophysical process in which a proton is transferred within a molecule from a donor site to an acceptor site upon electronic excitation.^2^ Typically, ESIPT involves the transfer of a proton from a hydrogen donor group (i.e., hydroxyl or amine group) to a nearby intramolecular acceptor group, such as a carbonyl or nitrogen atom. Some relevant examples of molecules which undergo the ESIPT process are presented in Figure 1.

**Figure 1:**
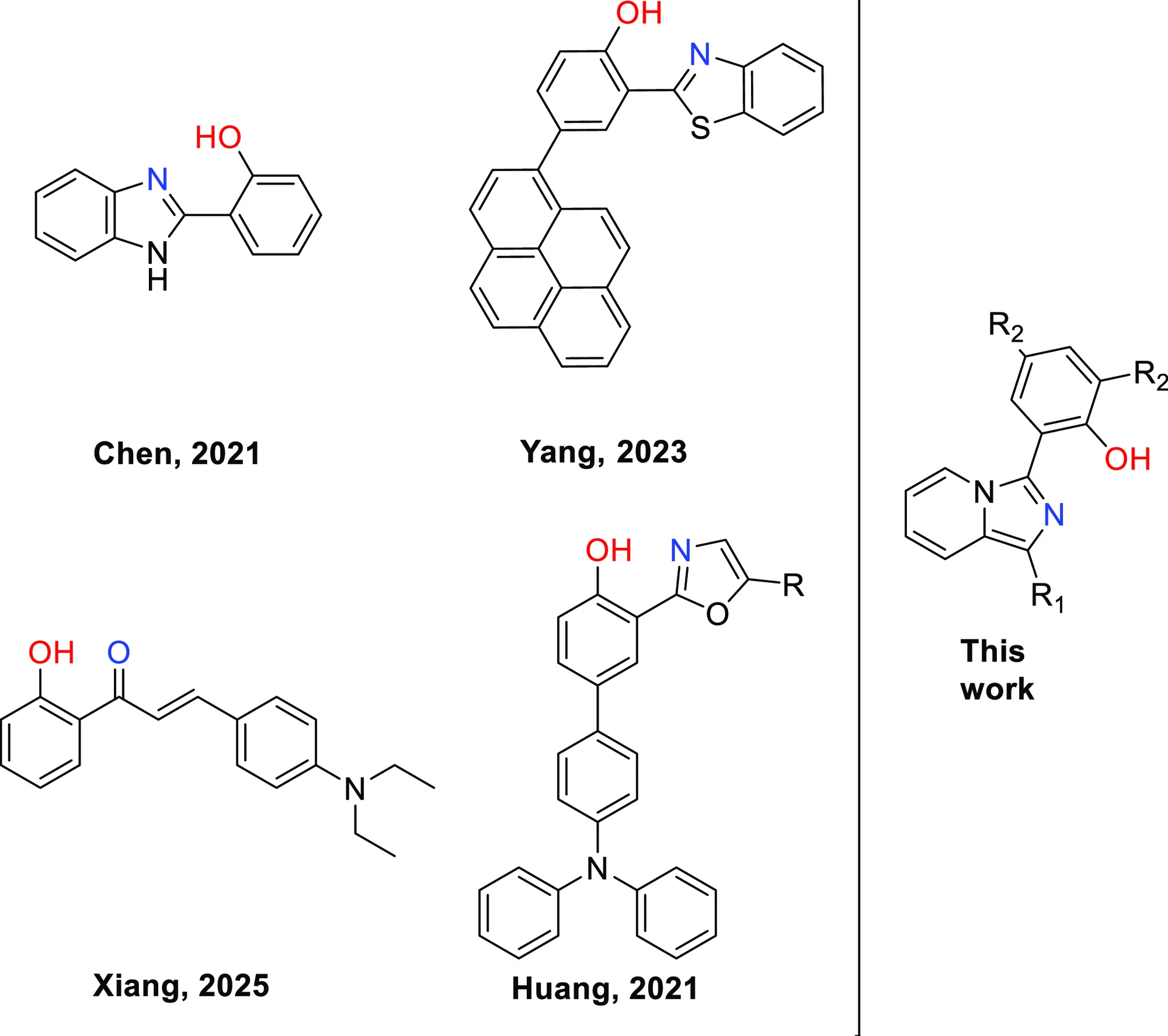
Relevant examples of compounds displaying ESIPT. Red represents the hydrogen bond donor, and blue represents the hydrogen bond acceptor.

Notably, this proton transfer occurs exclusively in the electronically excited state, with no analogous process occurring in the ground state. This phenomenon has garnered considerable interest due to its significant implications in the development of advanced fluorescent materials,^3^ molecular sensors,^4^ and photoswitches^5^ and its extensive applications in organic electronics and photophysics.^6,7^

ESIPT is an exceptionally fast process, often occurring within a femtosecond-to-picosecond time scale^8^ preceding most other excited-state relaxation pathways. This proton transfer induces a significant Stokes shift in the fluorescence spectrum, frequently spanning hundreds of nanometers.^9,10^ As a result, the absorption and emission spectra are well separated, with absorption typically originating from the enol form and emission arising from the tautomeric form of the molecule (Figure 2). The large Stokes shift, driven by excited-state structural reorganization, is a defining characteristic of ESIPT systems. It reduces the level of spectral overlap, thereby minimizing self-quenching and reabsorption effects. This makes ESIPT-active materials particularly appealing for high-efficiency fluorescence applications, such as organic light-emitting diodes (OLEDs)^11^ and dye lasers.^12^

**Figure 2:**
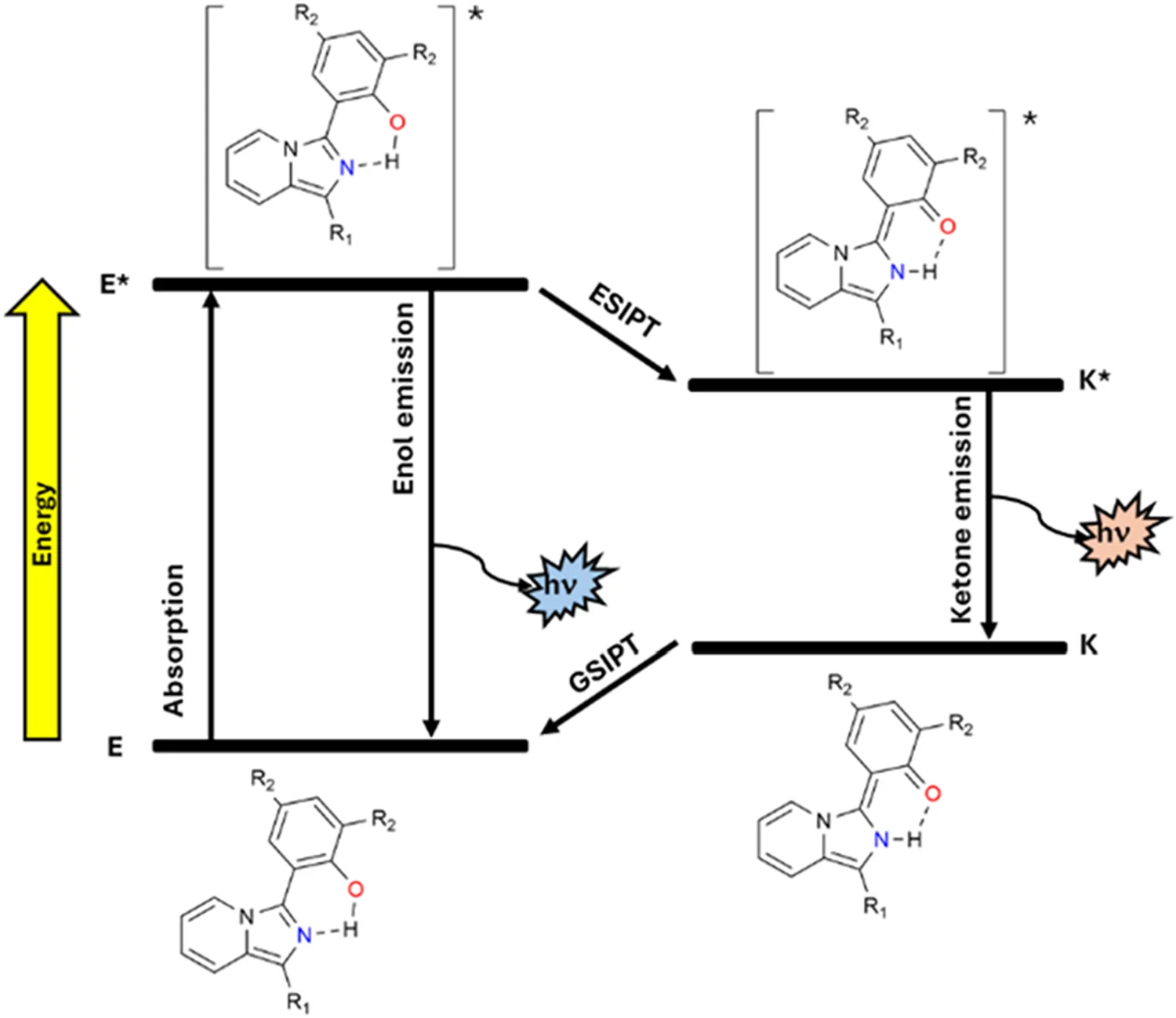
Schematic representation of the ESIPT process in imidazo­[1,5-*a*]­pyridine-3-yl phenols. E: enol ground state; E*: enol excited state; K*: ketone excited state; and K: ketone ground state.

One intriguing feature of certain ESIPT systems is the potential for dual emission, where both the enol and ketone forms emit light, generating a two-band fluorescence spectrum.^13–15^ This dual emission is highly sensitive to environmental variables such as solvent polarity,^16^ temperature, and (in solid state) molecular packing. Such sensitivity enhances the potential of ESIPT-based materials in molecular sensing and probing applications,^17,18^ while in the field of optoelectronics, dual emission opens the possibility of designing single-molecule white light emitters.^19,20^ Given the ability to fine-tune molecular properties to induce ESIPT, the design of novel ESIPT emitters remains a topic of significant scientific and technological interest. In this context, imidazo­[1,5-*a*]­pyridine phenols have emerged as a highly promising class of compounds for ESIPT applications. These heterocyclic systems are well-known for their advantageous photophysical properties,^21–23^ including large Stokes shifts, moderate to high fluorescence quantum yields, and substantial photostability. Additionally, imidazo­[1,5-*a*]­pyridines possess pharmacological relevance^24,25^ and exhibit significant synthetic versatility^26,27^ allowing for diverse functionalization of the core structure. This versatility makes them suitable for a broad range of applications,^28^ spanning from optoelectronic devices^29^ to molecular imaging probes.^30,31^

Our research group has extensively investigated the photophysical and coordination chemistry of imidazo­[1,5-*a*]­pyridine derivatives, particularly focusing on their use as ligands in luminescent coordination complexes.^32–35^ In the case of N,OH-molecules, where a phenol group is present, the potential for ESIPT emerges, offering an opportunity to further enhance the luminescence properties of these systems. Driven by this potential, we aimed to functionalize the imidazo­[1,5-*a*]­pyridine phenol scaffold to fine-tune its electronic and steric properties, with the goal of developing novel ESIPT emitters. In parallel, we also sought to establish a way to parametrize the contribution of different substituents, in order to predict the emergence of the ESIPT phenomenon. Beyond their immediate interpretative value, such models can provide a valuable predictive framework for anticipating the photophysical behavior of related compounds.^36,37^ By correlating structural features with functional outcomes, these models enable the rational design of new ESIPT-capable systems, reducing the reliance on trial-and-error synthesis and minimizing the need for resource-intensive computational or experimental screening. The results of this investigation are presented.

## Results and Discussion

### Syntheses and Characterization

The imidazo­[1,5-*a*]­pyridine derivatives were synthesized using different methodologies (Scheme 1). Compounds **1**, **5**, and **6** were obtained via the condensation of a 2-(aminomethyl)­pyridine derivative with a carboxylic acid, utilizing propanephosphonic acid anhydride (T_3_P) as the condensing agent.^38^ In contrast, compounds **2** and **3**,^21^ **4**,^39^ **7**, and **8** ^40^ were prepared through a condensation reaction between a pyridyl ketone and the substituted salicylic aldehyde in acidic medium, with ammonium acetate serving as the sp^2^ nitrogen source.^33^

**Scheme 1:**
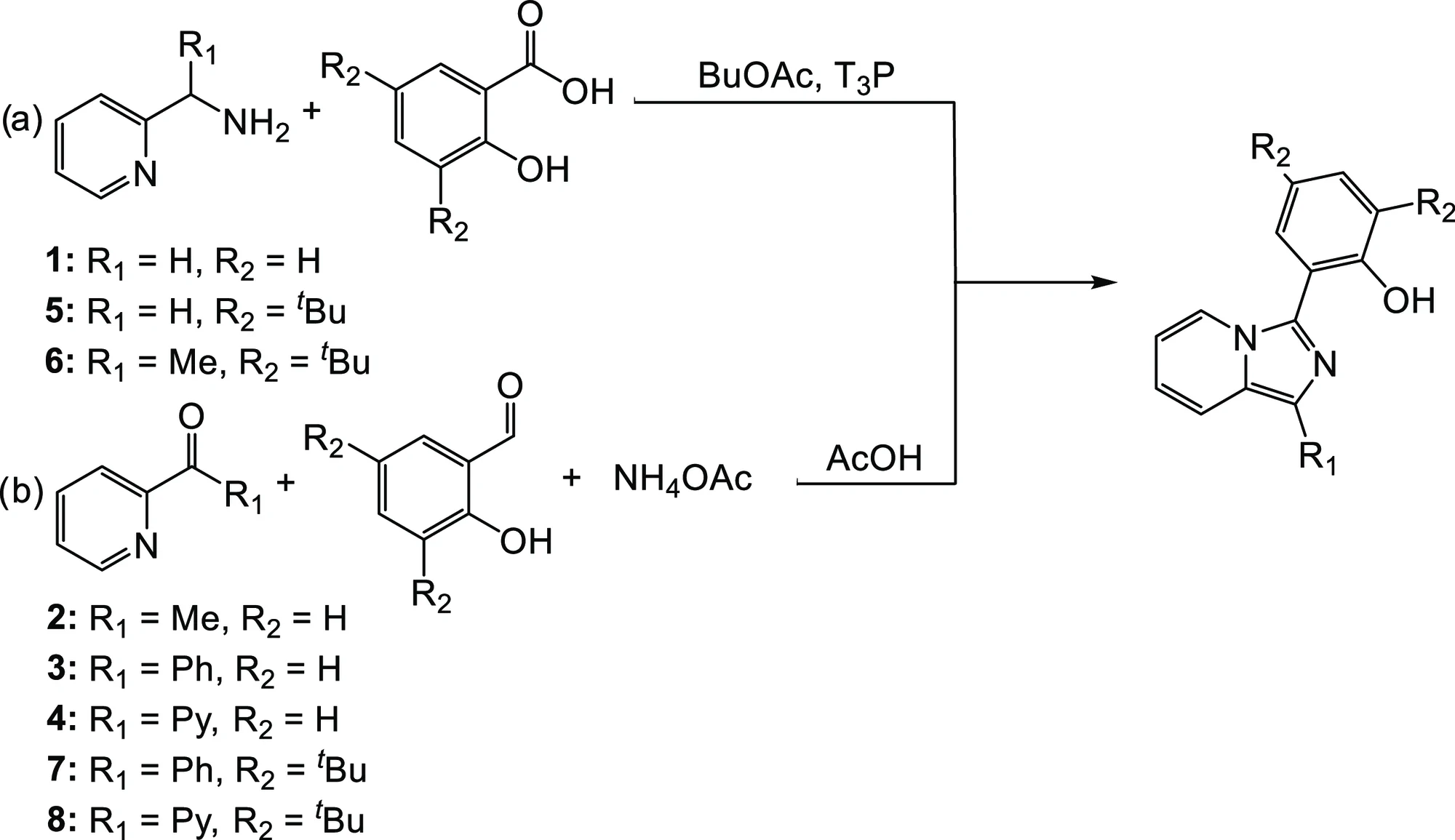
Synthetic Approach for Compounds **1**–**8**

The ^1^H NMR spectra of compounds **1**,^41^ **2** and **3**,^21^ **4**,^39^ **7**, and **8** ^40^ recorded in CDCl_3_ were in agreement with literature data. Compounds **5** and **6** exhibited the expected signals between 8.45 and 6.55 ppm, corresponding to the aromatic protons of the imidazopyridine core and the phenol ring, along with two singlets at 1.52–1.40 ppm, attributable to the *tert*-butyl groups. Additionally, compound **6** displayed a singlet at 2.56 ppm, assigned to the methyl group at position 1 on the heterocycle (Figures S1–S12).

Crystals of compound **7**, suitable for X-ray structure analysis, were obtained by slow evaporation of a saturated dichloromethane solution. Bond lengths and angles were found to be consistent with similar systems reported in the literature^21^ (Figures 3 and S33). The dihedral angle between the planes of the phenol ring and the imidazopyridine moiety was 33.6°, while the angle between the phenyl ring and the heterocyclic portion was notably smaller, at just 13.0°. This suggests that the phenyl ring may play a more significant role in the delocalization of the electron density over the ESIPT acceptor unit. The presence of an intramolecular hydrogen bond was confirmed by the short distance of 1.82 Å between the phenolic proton and the nitrogen atom at position 2 of the imidazopyridine ring, indicating a moderately strong interaction with a mostly electrostatic nature. Additional crystallographic data are provided in Table S1.

**Figure 3:**
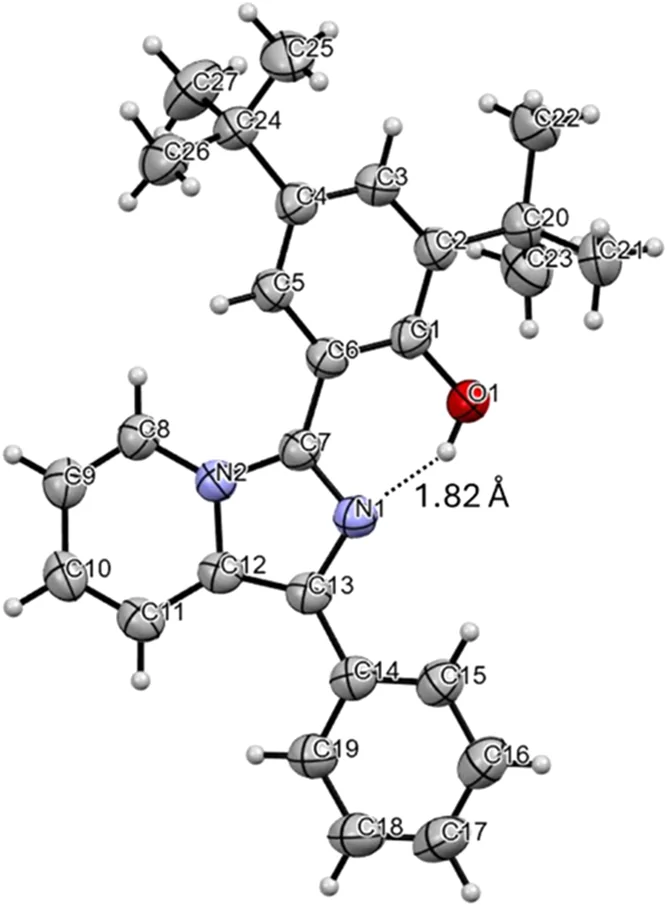
Crystal structure of compound **7** (pale gray, hydrogen; gray, carbon; blue, nitrogen; red, oxygen).

### Optical Properties in Solution

Compounds **1**–**8** exhibit similar absorption spectra (Figure 4), with a notable absorption band around 240 nm and a lower energy transition ranging from 329 to 343 nm. However, distinctive differences appear in the emission spectra: all derivatives show intense emission bands in the green-blue region, but compounds **5** and **8** exhibit a second lower energy emission band in the orange-red region. This additional band is attributed to the emission from the keto tautomer formed after proton transfer in the excited state, suggesting an ESIPT process. The emission spectra in dichloromethane solution (5·10^–5^ M) of compounds **1**–**8** are presented in Figure 4, and the CIE1931 chromaticity plot is reported in Figure S34, while detailed photophysical data are summarized in Table 1.

**Table 1:** Photophysical Data for Compounds **1-8** Recorded in Dichloromethane Solution (5·10^–5^ M)

	*R*_1_	*R*_2_	ε (M^–1^ · cm^–1^)	λ_abs_ (nm)	λ_exc_ (nm)	λ_em_ (nm)	stokes (cm^–1^)	stokes (eV)	Φ_PL_	τ (ns)	*k*_r_ (·10^7^ s^–1^)	*k*_nr_ (·10^8^ s^–1^)
**1**	H	H	49,970	341	357	464	7774	0.96	0.05	2.59	1.9	3.7
**2**	Me	H	8404	338	341	482	8839	1.09	<0.05	2.53	2.0	3.8
**3**	Ph	H	8626	343	345	484	8493	1.05	0.26	4.25	6.1	1.7
**4**	Py	H	29070	329	355	465	8889	1.10	0.40	3.32	12.0	1.8
**5**	H	^*t*^Bu	16,894	331	353	472, 598	9025, 13,489	1.12, 1.67	<0.05	0.50 (33.38%)	1.5^a^	2.9^a^
										4.70 (66.62%)		
**6**	Me	^*t*^Bu	19,305	339	341	490	9090	1.13	0.05	1.16 (92.76%)	3.2^a^	5.9^a^
										6.98 (7.24%)		
**7**	Ph	^*t*^Bu	13,108	340	342	491	9045	1.12	0.05	1.04 (95.1%)	4.3^a^	8.3^a^
										3.26 (4.9%)		
**8**	Py	^*t*^Bu	27,871	333	353	471, 628	8799, 14,106	1.09, 1.75	<0.05	0.27 (84.3%)	4.1^a^	7.9^a^
										6.27 (15.7%)		

a Calculated from the weighted average lifetime.

**Figure 4:**
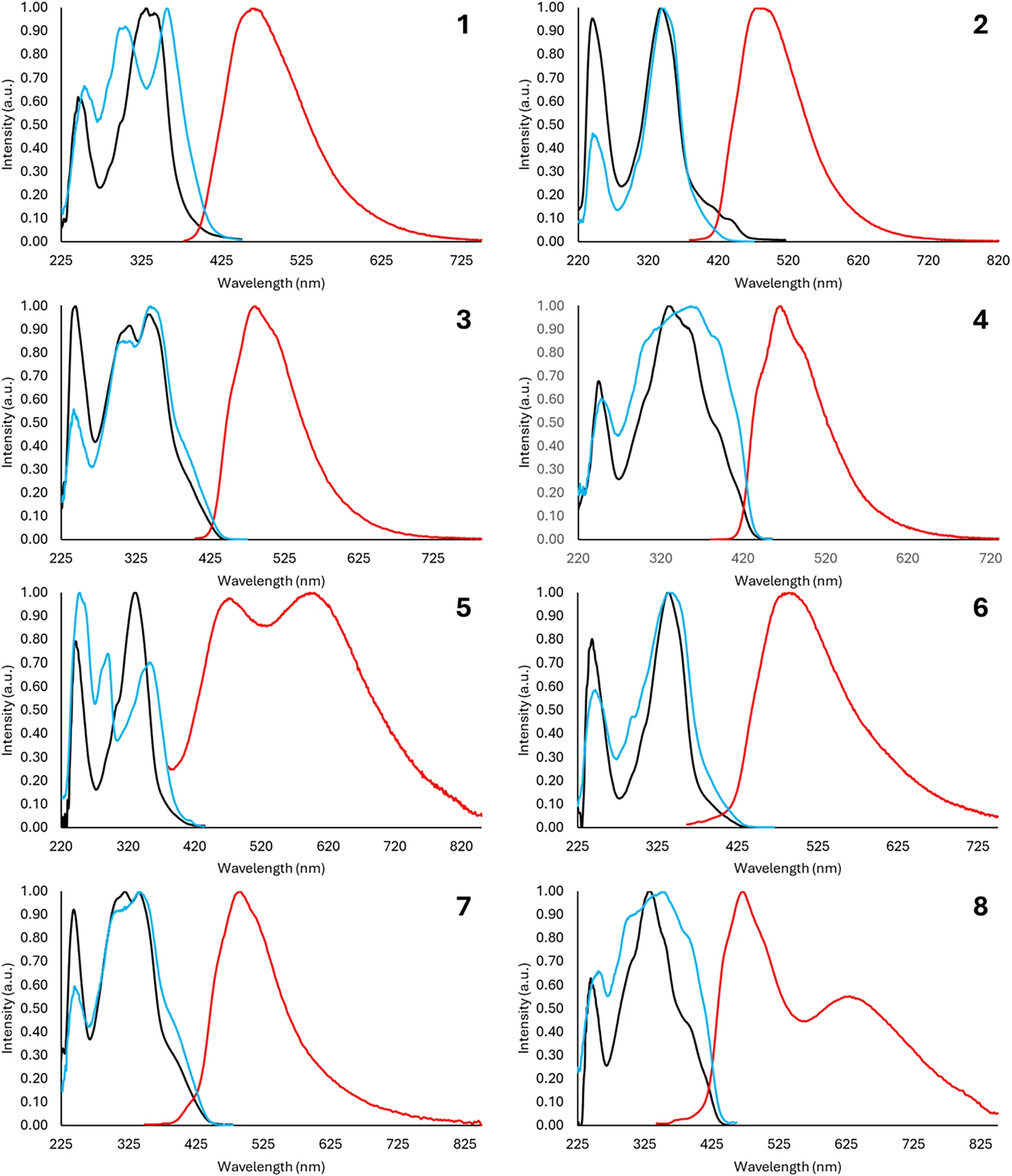
Normalized absorption (black), excitation (light blue), and emission (red) spectra of compounds **1**–**8** recorded in solution (CH_2_Cl_2_, 5·10^–5^ M).

Fluorescence lifetime decays are monoexponential for derivatives **1**–**4**, with values ranging from 2.5 to 4.3 ns. In contrast, derivatives **5**–**8** show biexponential decays, with shorter lifetimes between 0.27 and 1.16 ns and longer ones ranging from 3.26 to 6.98 ns. The difference between mono- and biexponential decays is likely due to the presence of *tert*-butyl groups, which may promote nonradiative decay pathways. Fluorescence quantum yields are notably higher for compounds **3** and **4**, while they are quite low for the other derivatives. In the case of compounds **1** and **2**, the low quantum yields could be due to less extensive electron delocalization compared to compounds **3** and **4**.

For the ESIPT-displaying compounds **5** and **8**, fluorescence lifetimes decays measured at the ESIPT emission wavelengths were very similar to those recorded at the standard emission (Table S2).

To investigate the ESIPT nature of the red-shifted emission, compound **8** was subjected to several experiments. First, fluorescence analyses were performed in solvents with varying polarities, and the emission profile was evaluated accordingly. The absorption spectra showed minimal changes, with absorption maxima ranging from 327 nm in methanol to 334 nm in toluene. However, the emission spectra displayed a stronger solvent dependence, with the first emission maximum (i.e., the so-called standard emission) shifting from 451 to 471 nm and the second (ESIPT emission) shifting from 581 to 641 nm. Notably, the red-shifted emission is effectively suppressed in polar or protic solvents such as acetone, acetonitrile, and methanol. In contrast, the ESIPT process persists in nonpolar media including dichloromethane and toluene. Interestingly, in solvents such as THF and 2-propanol, the quenching effect is present but significantly less prominent. In the specific case of isopropanol, despite the presence of a hydroxyl group that could potentially disrupt the intramolecular hydrogen bond, the steric bulk of the secondary alcohol likely hinders its approach to the ESIPT-active site.^42^ This prevents the full suppression of the tautomeric emission, unlike what is observed with the sterically unhindered methanol (Figure S35, and Table S2).

The ESIPT emission, when compared to normalized standard emission (Figure S36), did not show a clear correlation, aside from a general trend of decreasing intensity in more polar solvents. To confirm this, additional experiments were carried out by measuring the emission spectra of compound **8** in dichloromethane/methanol mixtures at different ratios, in acidic media using tetrafluoroboric acid in dichloromethane, and after deuteration. As expected, both deuteration (Figure S37) and protonation (Figure 5a) affected the ESIPT emission, which was diminished and quenched. Increasing the methanol percentage in dichloromethane (Figure 5b) initially caused a general decrease in fluorescence intensity, followed by an increase and blue shifting of the standard emission (Figure S38), while the ESIPT emission was gradually suppressed, resulting in an overall relative decrease in the ESIPT emission and further confirming the occurrence of such a process.

**Figure 5:**
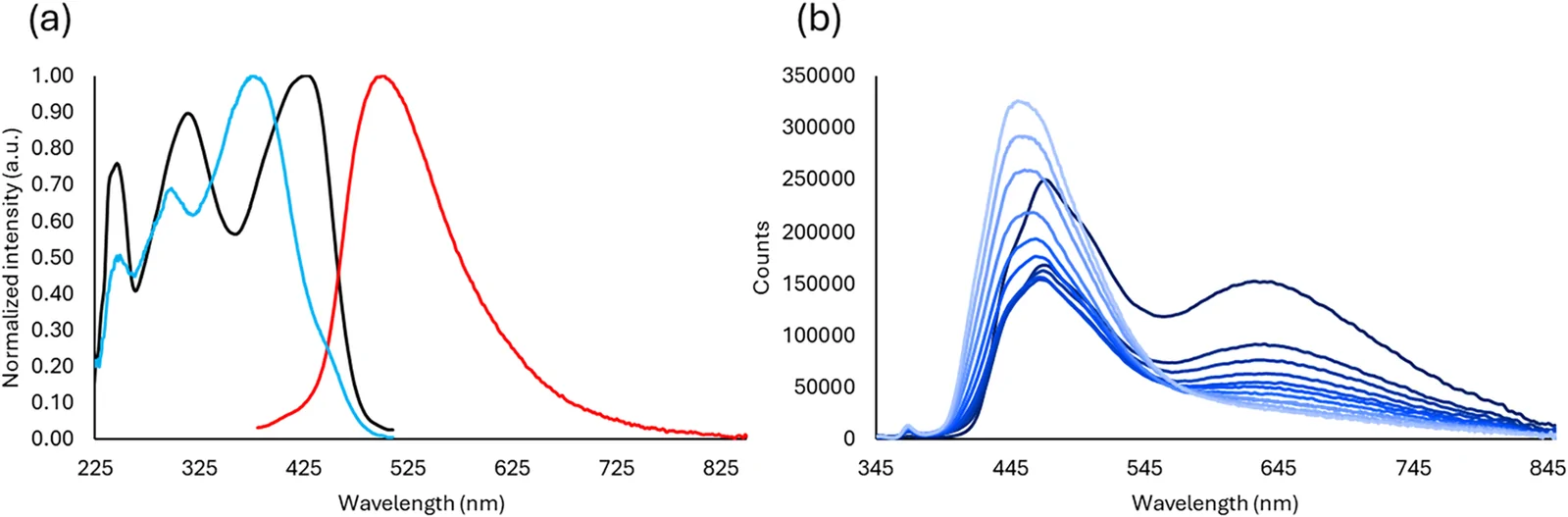
(a) Absorption (black), excitation (light blue) and emission (red) spectra of compound **8** measured in dichloromethane acidic conditions. (b) Emission profile of compound **8** measured in dichloromethane/methanol solution with different solvent ratios. Starting point (darkest blue line): 0% methanol. End point (lightest blue line): 100% methanol.

On the contrary, measuring the luminescence properties of compound **8** in basic medium, obtained by adding an excess of tetrabutylammonium hydroxide in dichloromethane, showed the rising of a new emission band slightly blue-shifted when compared to the one due to the ESIPT emission (Figure 6). This ruled out the possibility that the lower energy emission band originally observed in dichloromethane could be due to a deprotonation phenomenon^13^ caused by the different acid–base properties of the excited state with respect to those in the ground state. This was also further corroborated by the theoretical determination of the emission maxima (Table S4).

**Figure 6:**
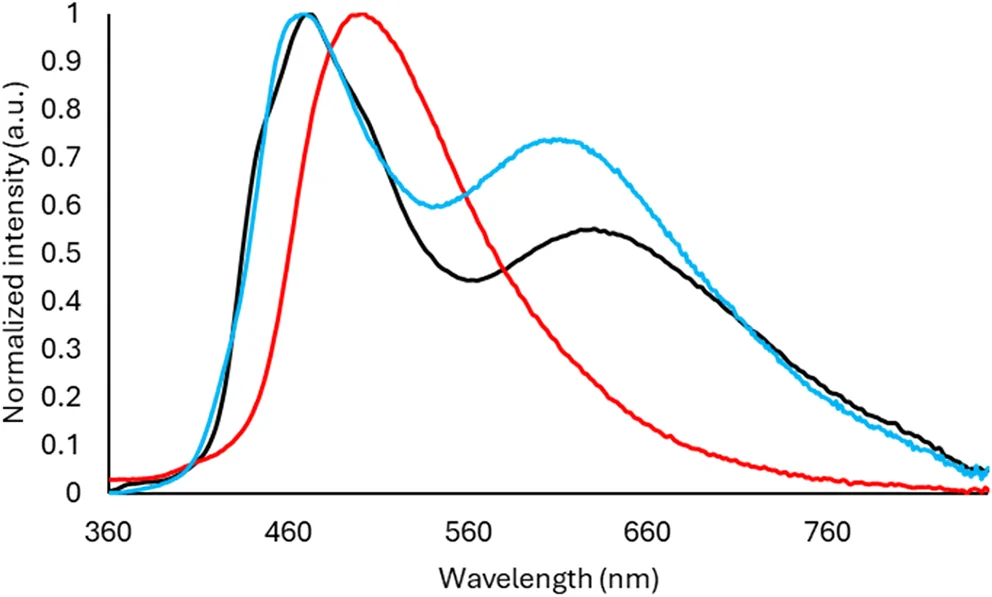
Normalized emission profiles of compound **8** measured in dichloromethane solution (black: neutral environment; red: acidic environment; light blue: basic environment).

Additionally, two experiments were performed to establish whether hindering the rotation of the phenol group over the imidazo­[1,5-*a*]­pyridine core, thus keeping the phenol in a favorable conformation, would favor the ESIPT process even in compounds not showing such behavior (e.g., compound **2**). First, compound **9** was synthesized starting from 2-acetyl-6-methylpyridine (Scheme 1b), so that a methyl group ortho to the endocyclic nitrogen was introduced in the imidazo­[1,5-*a*]­pyridine skeleton. The presence of two methyl groups, pointing toward the same direction, should hinder the rotation, favoring the correct conformation for the ESIPT process to take place. However, compound **9** displayed only blue emission with no detectable ESIPT phenomenon when measured in dichloromethane solution (Figure S39). An additional experiment was carried out on compound **2** by recording emission spectra in dichloromethane solution upon the gradual addition of eicosane, up to a concentration of 0.1 g/mL, in order to increase the viscosity of the solution (Figure S40). In principle, increasing the viscosity of the medium should progressively hinder the thermal rotation of the two moieties,^43^ thereby helping to maintain the hydroxyl group in the appropriate position to sustain proton transfer for a longer time. Nonetheless, this did not result in any ESIPT emission, leading us to conclude that the unique behavior of compounds **5** and **8** could be attributed solely to their electronic properties.

To further assess the ESIPT phenomenon, fluorescence analyses at different temperatures were conducted. Both compounds **5** and **8** were dissolved in 2-methyl tetrahydrofuran, and their fluorescence emissions were recorded as the temperature varied from 25 to 65 °C (Figures 7 and S41). In general, an increase in emission intensity was observed with the rising temperature. Since the standard and ESIPT emissions partially overlapped, spectral deconvolution and integration of the two emission profiles were necessary to evaluate relative changes (Figure S42). A slight decrease in ESIPT emission intensity relative to the standard one was observed, but it was too small to be considered significant (Figure S43). Based on this and corroborating experimental evidence, such as the very similar lifetime decays measured for both emission maxima and the minimal separation between the emission profiles, the ESIPT process was confirmed to be thermodynamic in nature.^44^

**Figure 7:**
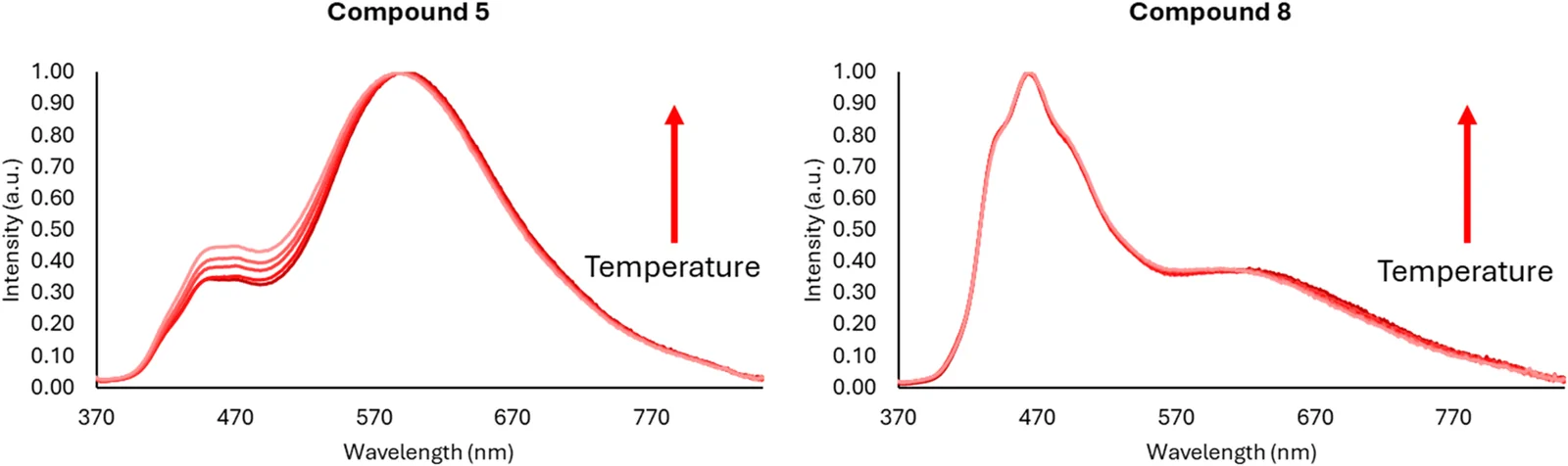
Normalized emission spectra of compounds **5** and **8** measured in 2-methyl tetrahydrofuran (5·10^–5^ M) at different temperatures.

Compounds **2** and **7** were also analyzed at various temperatures, but no ESIPT emission was observed, aside from a general increase in the fluorescence intensity (Figure S44).

To gain deeper insights into the ESIPT mechanism, fluorescence spectra of the ESIPT-active compound **8** and the ESIPT-inactive derivative **7** were recorded at low temperatures (from 298 to 193 K) in dichloromethane solution (Figure S45). For compound **8**, the dual emission persists across the entire temperature range, confirming the intrinsic nature of the process. However, a systematic decrease in the keto-to-enol (K*/E*) intensity ratio was observed, shifting from **∼**0.54 at RT to **∼**0.47 at 193 K (Figure S46). This trend suggests that although the process is thermodynamically driven with a low calculated energy barrier (*vide infra*), the increased solvent viscosity and reduced thermal energy at low temperatures partially hinder the structural reorganization required to reach the keto-like excited-state geometry. In contrast, compound **7** remained exclusively in its enolic form, even at 193 K.

### Optical Properties in the Solid State

The excitation and emission spectra of compounds **1**-**8** in the solid state are collected in Figure S47.

In the solid state, the compounds under investigation exhibited a broader emission range from 423 to 578 nm (Figure S48). Notably, while most of the compounds showed blue-green emission, compound **2** stood out with an emission in the orange region of the spectrum, distinguishing it from the others. Compound **9**, on the other hand, is nonemissive in the solid state.

Interestingly, the fluorescence quantum yields of compounds **7** and **8** increased, likely due to the enhanced rigidity of the molecules within the crystal lattice. However, none of the previously observed ESIPT emissions were detected during fluorescence analysis in the solid state: ESIPT requires a significant structural rearrangement in the excited state to stabilize the tautomeric form. Specifically, in this case, the phenol ring rotates to become almost perpendicular to the imidazopyridinic moiety in the excited state following the proton transfer (see coordinates in the Supporting file). Within a rigid crystal lattice, however, the molecules are frozen in their ground-state enolic conformation. The restrictive molecular environment of the solid state imposes a high kinetic barrier to this necessary reorganization, effectively quenching the ESIPT pathway.

Fluorescence lifetimes are always biexponential for species **1**-**4**, with the shorter ones ranging between 0.48 and 2.54 ns and the longer ones ranging between 2.28 and 6.48 ns. Apparently, compounds **3** and **4** displayed fluorescence lifetimes more similar between each other than those of compounds **1** and **2**. This could be due to the similar steric hindrance of the R_1_ substituent, which affects the intermolecular interactions within the solid lattice.

Analogously, the compounds bearing the *tert*-butyl groups on the phenolic ring displayed monoexponential lifetime decays ranging from 2.64 and 5.75 ns. Also, in this case, the presence of such bulky groups could in principle affect the solid conformation of these species, thus impairing molecular interactions. On the contrary, compound **8** displayed a biexponential lifetime decay similar to those of compounds **1**-**4**. Complete photophysical data recorded in the solid state are reported in Table 2.

**Table 2:** Photophysical Data for Compounds **1**-**8** Recorded in the Solid State

	*R*_1_	*R*_2_	λ_exc_ (nm)	λ_em_ (nm)	Φ_PL_	τ (ns)
**1**	H	H	364	423	<0.05	2.54 (76.45%)
						6.48 (23.55%)
**2**	Me	H	218	578	<0.05	1.04 (87.03%)
						3.20 (12.97%)
**3**	Ph	H	406	473	0.06	0.99 (69.54%)
						2.65 (30.46%)
**4**	Py	H	403	461	<0.05	0.48 (89.64%)
						2.28 (10.36%)
**5**	H	^*t*^Bu	405	471, 500	<0.05	2.64
**6**	Me	^*t*^Bu	417	487	<0.05	3.66
**7**	Ph	^*t*^Bu	448	503	0.17	5.75
**8**	Py	^*t*^Bu	430	501	0.13	2.79 (82.63%)
						6.17 (17.37%)

### Optical Properties in Thin Films

Fluorescence analysis of the compounds in thin films was performed with films made of emissive materials embedded in polymeric matrices (5% w/w in poly­(methyl methacrylate), PMMA, or polystyrene, PS). The emission spectra of the films showed some differences when compared to those recorded in solution or in the solid state (Table S5). Notably, dual emission was clearly observed for compound **2** in both the PMMA and PS matrices. In contrast, compounds **5** and **8**, which exhibited distinct ESIPT emission in solution, showed only broader emission peak profiles in the polymer films (Figures S49–S50). The dual emission for these species became slightly more pronounced when PS was used, likely due to π–π interactions that favored the orientation of the phenol ring and promoted ESIPT emission. Nevertheless, the emission range of the whole series is more similar to the one observed for the analysis in solution than to the one registered in the solid state (Figures S51–S52).

Interestingly, the fluorescence quantum yields of these systems increased compared to those recorded in solution (Table S5). This is well-known for ESIPT materials, as ESIPT usually triggers detrimental nonradiative decay paths, quenching the luminescence in solution. On the other hand, ESIPT dyes often increase their luminescence performances in the solid state or in polymeric thin films.^45^

### DFT Calculations

The current study utilized density functional theory (DFT) calculations to elucidate the mechanisms underlying the absorption and excited-state intramolecular proton transfer (ESIPT) processes observed in the compounds. Geometry optimization was performed by using the BLYP-D3­(BJ) functional. Theoretical calculations have shown that the most stable form in the ground state is the phenol (as also confirmed experimentally, *vide infra*), with a conformation that allows the O–H···N interaction (see the *xyz* coordinates in the SI and Table S6 for the relative energies).

In accordance with experimental results (^1^H NMR), all derivatives exhibited greater stability in the enol form in the ground state, displaying varying degrees of coplanarity between the phenolic and imidazopyridinic moieties. Based on distances and energies (Table S7), the O–H---N hydrogen bonds were classified as moderate according to Jeffrey’s criteria,^46^ thereby predisposing these molecules to the possibility to undergo proton transfer in the excited state.

To further rationalize the observed photophysical behavior and the propensity for proton transfer, we also analyzed the frontier molecular orbitals (FMOs) (Figure 8), providing insights into the electronic distribution and the nature of the electronic transitions involved.

**Figure 8:**
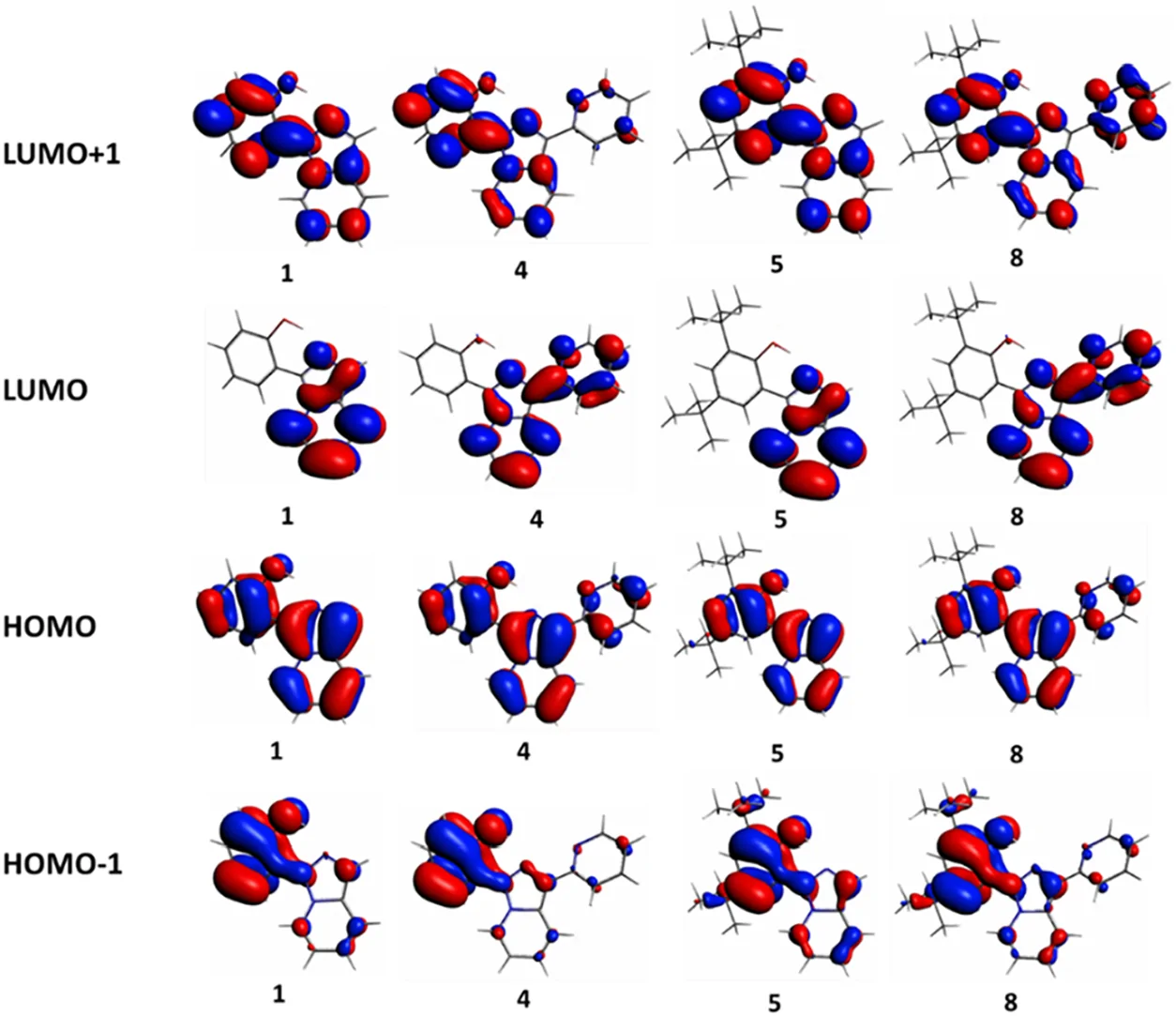
Frontier orbitals of compounds **1**, **4**, **5**, and **8** as representative examples. See Figure S53 for the whole series.

The FMOs displayed consistent topological features across all of the derivatives investigated. Specifically, the HOMO–1 was primarily localized on the phenolic moiety, although derivatives bearing *tert*-butyl substituents exhibited a slightly increased contribution from the pyridinic ring of the imidazopyridinic portion. The HOMO was uniformly delocalized over the entire molecular framework, while the LUMO was mainly distributed over the imidazopyridine portion and extended to the aromatic substituents when present. LUMO+1 also showed delocalization over the entire molecular structure.

Comprehensive details regarding the energy levels of the FMOs are listed in Table 3.

**Table 3:** Compound **1**-**8** HOMO and LUMO Orbitals’ Energies Both in the Ground (S_0_) and First Excited (S_1_) States, Computed at the DFT/BLYP-D3­(BJ) Level of Theory

	HOMO–1 S_0_	HOMO S_0_	LUMO S_0_	LUMO+1 S_0_	HOMO S_1_	LUMO S_1_	Δ*H*-L S_0_	Δ*H*-L S_1_	ESIPT
**1**	–5.600	–4.718	–2.011	–1.855	–4.466	–2.368	2.707	1.851	No
**2**	–5.518	–4.580	–1.963	–1.828	–4.303	–2.332	2.616	1.917	No
**3**	–5.525	–4.604	–2.165	–1.883	–4.397	–2.506	2.440	1.891	No
**4**	–5.541	–4.702	–2.278	–1.904	–4.512	–2.568	2.424	1.944	No
**5**	–5.280	–4.622	–1.993	–1.723	–3.841	–2.677	2.629	1.164	Yes
**6**	–5.228	–4.483	–1.958	–1.688	–4.224	–2.325	2.525	1.899	No
**7**	–5.235	–4.527	–2.157	–1.761	–4.330	–2.478	2.370	1.851	No
**8**	–5.260	–4.615	–2.265	–1.793	–3.752	–3.005	2.350	0.746	Yes

Time-dependent density functional theory (TD-DFT) calculations were employed to correlate theoretical predictions with experimental observations. The UV–visible spectra for the entire series were simulated in dichloromethane solution, yielding a favorable agreement with the experimental data (Figures 9 and S54 for the whole series). Spectral analysis indicated that the observed transitions predominantly originated from HOMO–LUMO transitions, although there was also a minor contribution from other orbitals, including HOMO–1 and LUMO+1 (Table S8).

**Figure 9:**
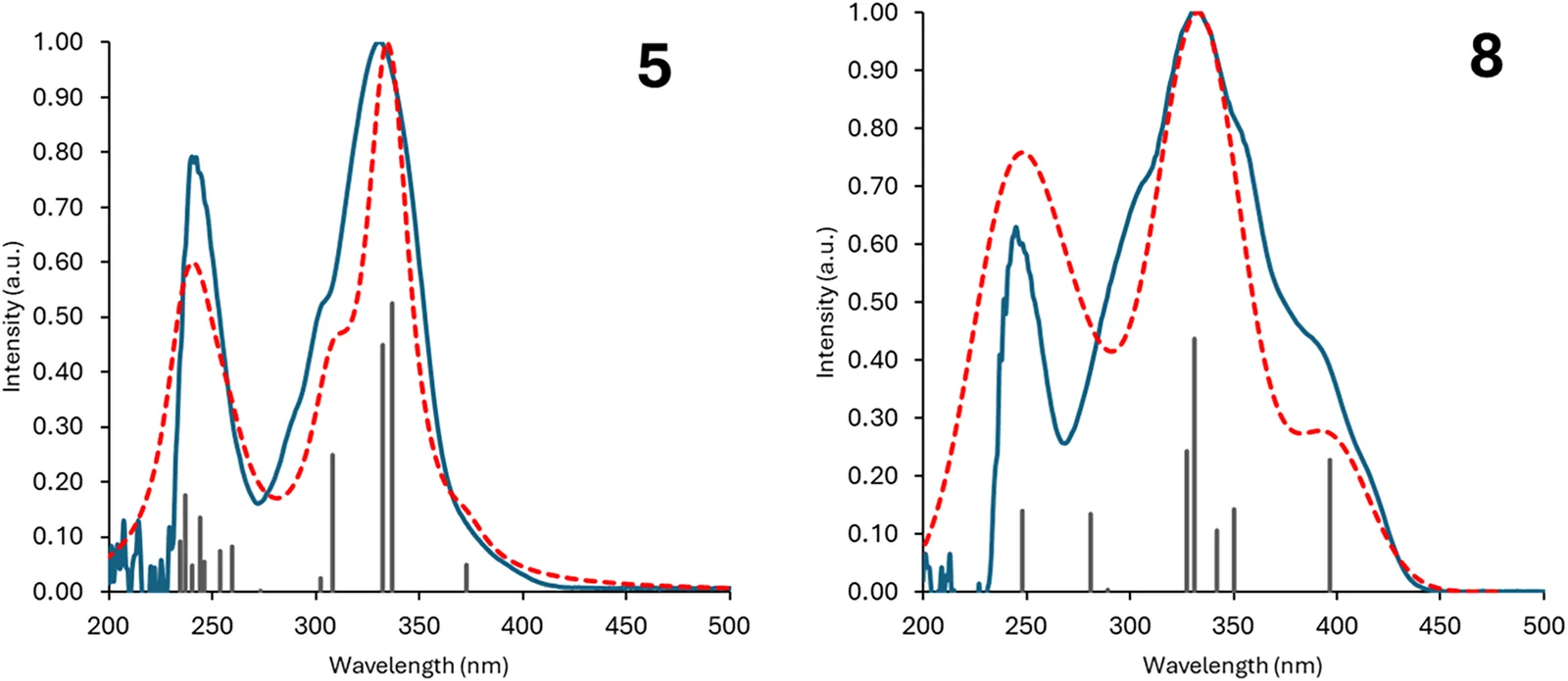
Experimental (solid blue line) and calculated (dashed red line) UV–vis spectra of compounds **5** and **8** in a dichloromethane solution. Vertical bars represent calculated transitions with an oscillator strength of *f* > 0.05.

A deeper analysis was performed to elucidate the distinct behavior toward the ESIPT of compounds **5** and **8** compared to the other species in this series. According to the optimized structures of the S_1_ state (Figure 10, see *xyz* coordinates in the SI for the whole series and Table S6 for the relative energies), the BLYP-D3­(BJ) functional successfully predicted the occurrence of the ESIPT process solely for these two compounds.

**Figure 10:**
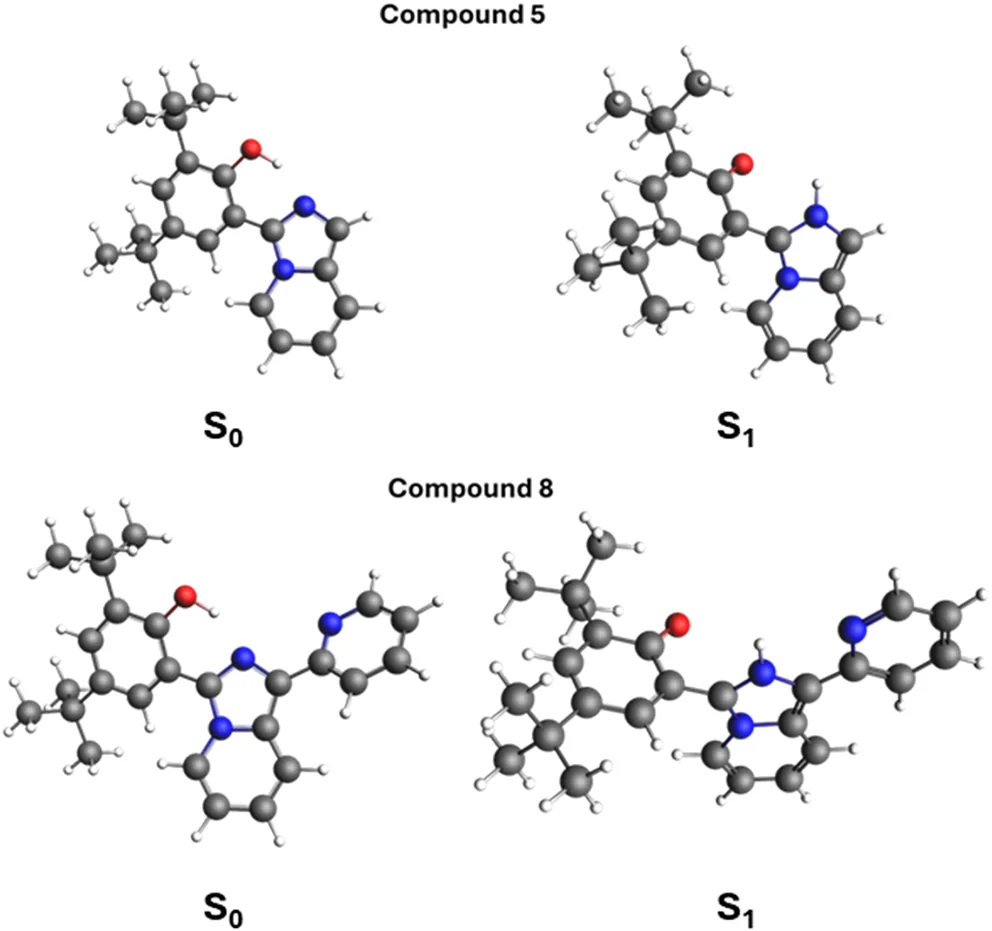
Computed optimized molecular structure of compounds **5** and **8** in the ground state (S_0_) and in the first singlet excited state (S_1_).

Potential energy scans (PESs) from the enol form (E) to the keto form (K) were computed in dichloromethane solution for derivative **8**, elongating the oxygen···hydrogen bond length of the OH group toward the nitrogen atom in the imidazole ring from 0.8 to 2.2 Å in 0.14 Å steps. In the ground state (S_0_), the enol form is predicted to be more stable, while the keto form becomes more stable in the first excited state (S_1_). As illustrated in Figure 11, the transition from the enol to the keto form in S_1_ is barrierless, with a reverse barrier of 0.56 eV (13.4 kcal/mol), which ultimately favors the ESIPT process and fluorescence emission from the keto form.

**Figure 11:**
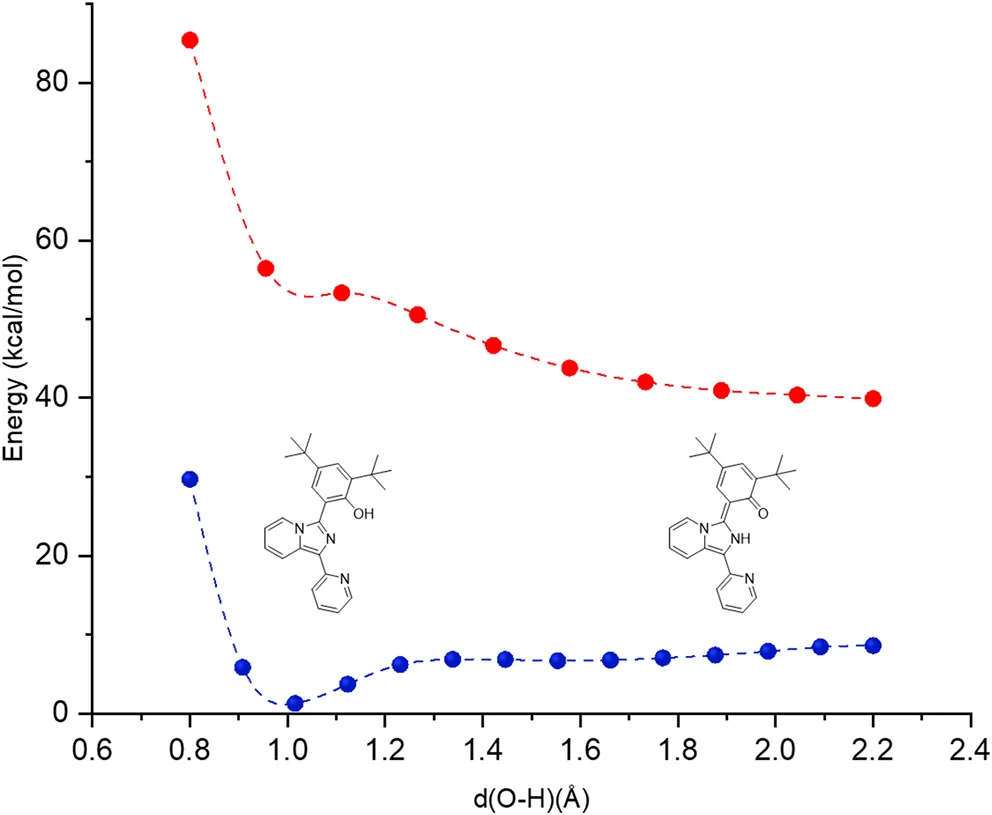
PES curves computed for compound **8** in the ΔE scale at the BLYP-D3­(BJ) level of theory in CH_2_Cl_2_ solution. Blue trace: ground state; red trace: excited state.

However, even compound **7** that does not exhibit ESIPT led to similar considerations, with PES curves resembling those of the derivative and **8** (Figure S55) and an energy barrier not sufficient to rule out the possibility to experience the ESIPT process (2.50 kcal/mol, 0.11 eV). Moreover, when calculating the energies of the phenol and keto forms, in both the ground and excited states (Figure S56), it was not possible to obtain a result capable of discriminating and rationalizing why only two of the proposed derivatives displayed this behavior. In fact, although these two methods (PES scans and evaluation of the energies of the different forms) are among the most commonly used in the literature^47^ to explain this phenomenon, they were found to be unsuitable for application to the systems under study. This would come with no surprise, as imidazo­[1,5-*a*]­pyridines are not known for their ESIPT properties, differently from imidazo­[1,2-*a*]­pyridines.^48–50^ For this reason, it was necessary to search for the discriminating factor by other means.

By analyzing the energies of the frontier orbitals in both the S_0_ and S_1_ states, a clear trend emerges. While the S_0_ state does not exhibit a notable trend on orbital energies, the HOMO and LUMO in the S_1_ state are significantly stabilized in the derivatives following the ESIPT process. This effect is particularly evident in the Δ­(HOMO–LUMO), which is consistently lower compared with derivatives that do not undergo proton transfer (Table 3). This reduction is attributed to electronic redistribution following proton transfer, leading to a marked stabilization of the LUMO in the excited state (keto form), although the HOMO is also affected, but in a less pronounced way. Consequently, the reduced energy gap facilitates greater electronic delocalization, thereby stabilizing the species.

By examining the electron density difference maps (EDDMs), we can further confirm that in the ESIPT-active species, significant electron density transfer occurs from the donor to the acceptor unit (Figure 12). Specifically, photon absorption leads to a notable depletion of electron density on the hydroxyl group, rendering it more acidic, and a concurrent increase on the nitrogen atom of the acceptor moiety, enhancing its basicity. This redistribution of electron density alters the relative acidities of the two groups in a manner that promotes ESIPT.

**Figure 12:**
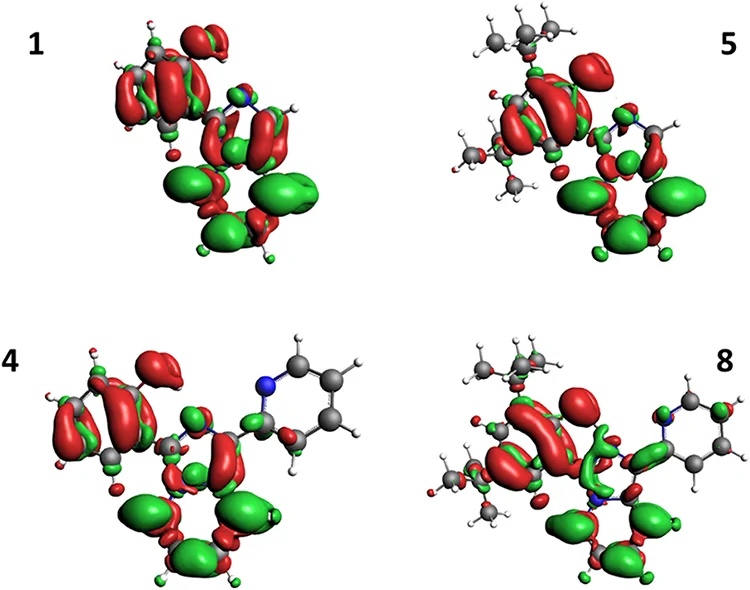
Electron density difference maps (EDDMs) for the lowest energy singlet electronic transition computed by TD-DFT (**red** indicates a decrease in electron density, **green** indicates an increase) for compounds **1**, **4**, **5**, and **8**. See Figures S57–S58 for the whole series.

Although this behavior is observed across the entire series, it is particularly evident that the presence of *tert*-butyl substituents greatly enhances the electron density on the imidazole ring within the imidazopyridine core. In contrast, in the absence of electron-donating groups on the phenol ring, the same imidazole moiety exhibits a marked decrease in electron density. Similarly, the introduction of a pyridine substituent on the imidazopyridine ring facilitates electron acceptance, thereby mitigating the extent of the electron density loss on the imidazole ring.

However, both the stabilization due to smaller Δ­(HOMO–LUMO) in the S_1_ state and the considerations from the EDDM analysis were not suitable to explain the origin of the ESIPT phenomenon, as the difference results from the fact that the ESIPT has already taken place, and they are a direct consequence of the process (i.e., the generation of the keto form).

### ESIPT Parametrization

The compounds exhibiting ESIPT in this series demonstrated low fluorescence quantum yields, which limit their potential applications in the fabrication of efficient optoelectronic devices and molecular sensors. This limitation can be attributed to the presence of *tert*-butyl groups, known for their luminescence quenching effects.^51^ However, these groups appear to be essential for facilitating the ESIPT process, as strong electron-donating groups are required to eventually increase the electron density in the acceptor moiety.

To enable the rational design of analogous compounds based on the imidazo­[1,5-*a*]­pyridine-3-yl phenol scaffold that exhibit ESIPT emission, potentially without relying on sterically demanding substituents such as *tert*-butyl groups, a theoretical investigation aimed at identifying general molecular descriptors predictive of the ESIPT behavior would be of significant value. In this context, a structure–property relationship approach could serve as a powerful tool to correlate structural and electronic features with the propensity of a given molecule to undergo ESIPT. By establishing such correlations, it may become possible to guide the design of novel systems, including potential white-emitting fluorophores with improved photophysical properties, such as higher fluorescence quantum yields and tunable emission profiles.

At this point, in order to refine the discrimination, other species were added to the *in silico* analysis, as well as compound **9**, precedingly synthesized (Table 4).

**Table 4:**
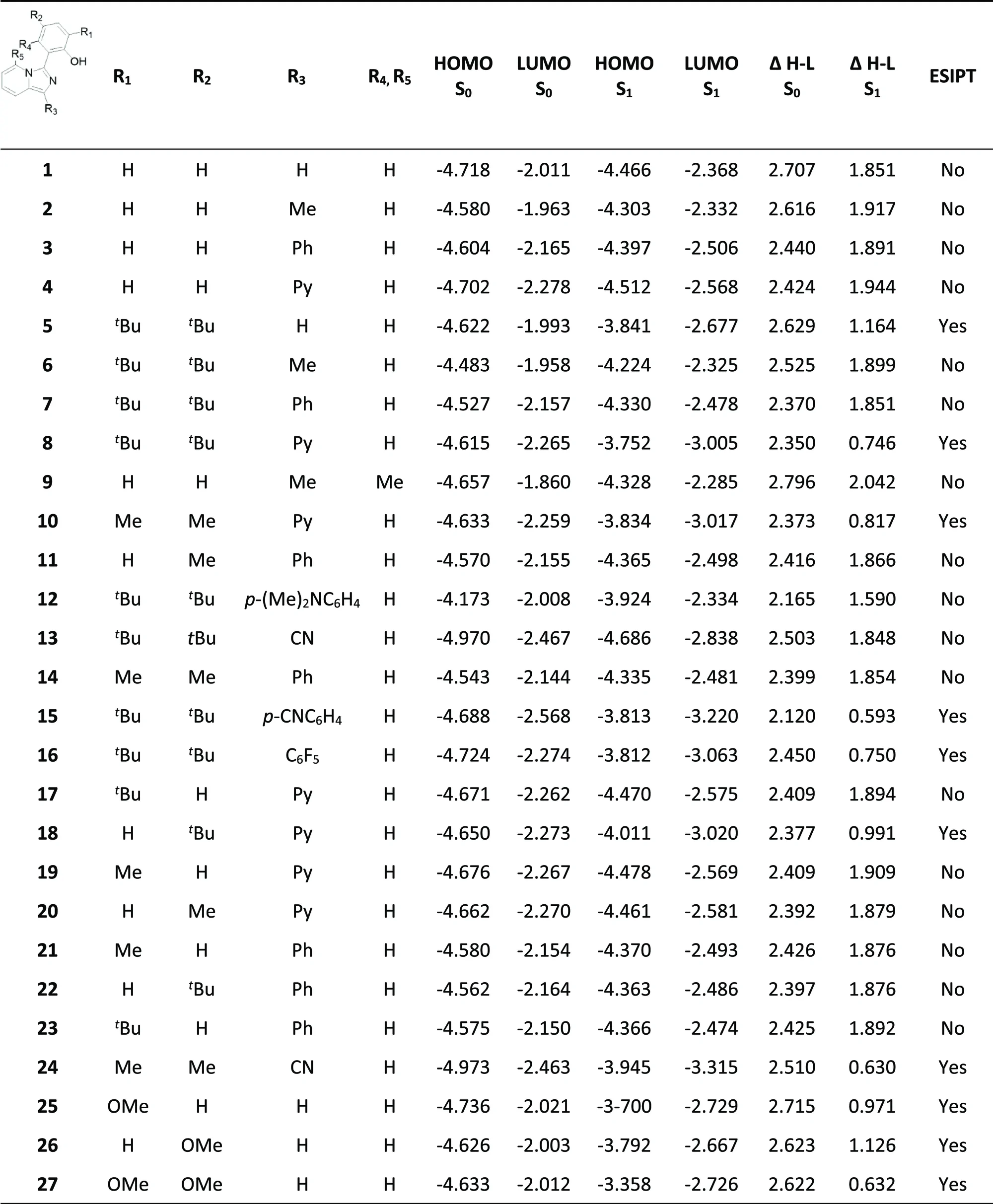
HOMO and LUMO Orbitals’ Energies Both in the Ground and First Excited States, Computed at the DFT/BLYP-D3­(BJ) Level of Theory, for All the Derivatives Considered in This Work

It seemed quite evident that the substituents played a role: in fact, electron-donating substituents on the phenol and electron-accepting or neutral substituents (such as hydrogen in compound **5**) on the imidazopyridine are required, but this is not the only requirement. For instance, derivative **13**, which has a cyanide substituent, does not undergo ESIPT, even though cyanide is a strong electron-withdrawing group, while compound **24**, which should have a weaker push–pull effect, as methyl groups are less electron-donating than *tert*-butyls, does present ESIPT. This is probably because the accepting group should be able not only to attract the electron density but also to delocalize it, thus increasing the stability of the system. Reasonably, having the stronger electron-donating force of the *tert*-butyl groups, paired with the weaker ability of the cyanide with respect to aromatic groups to delocalize the electronic density, results in the destabilization of the system, preventing ESIPT from occurring. On the other hand, the milder electron-donating power of the methyl groups allows the phenomenon to take place, with a sort of Goldilocks effect.

For this reason, we decided to identify a parameter as a function of the substituents capable of predicting the occurrence of the ESIPT process. This required the establishment of a system of equations that relates the presence of specific substituents to a physical observable capable of effectively discriminating the occurrence or absence of the ESIPT process. From previous orbital energy calculations, it could be observed that the only significant difference lies in the Δ­(HOMO–LUMO) values in the S_1_. However, as stated above, this variable was not discriminating, as the difference results from the fact that ESIPT has already taken place. Therefore, the only remaining discriminating physical observable is the actual manifestation of the ESIPT phenomenon, an outcome that rather than being a trivial confirmation emerges as the ultimate and irreducible criterion to distinguish between systems inherently predisposed to undergo the process and those fundamentally incapable of it.

Then, a system of equations (Table S9) to relate ESIPT to parameters associated with the substituents was established as follows

1 $$f_{\left(\mathrm{ESIPT}\right)}=\sum_{i}{C_{i}\cdot \alpha }_{i}$$

where *f*_(ESIPT)_ is the function defining the observed physical variable, which takes the value 1 when ESIPT is present and 0 when it is absent for a given derivative, thus applying a Boolean approach; *C_i_* represents the coefficients indicating the presence of the different substituents, taking the value 1 if the substituent is present in the right position in the derivative and 0 if it is absent; and *α_i_* represents the values associated with the individual substituents, obtained by solving the system through linear regression.

Once the values associated with the individual substituents (normalized with respect to *p*-H and *o*-H, which both displayed a value of 0) were obtained (Table 5), we were able to calculate a new parameter (Ω) using the α_i_ values of the substituents present in a given derivative as follows

2 $$\Omega =\sum_{i}\alpha_{i}$$

**Table 5:** α Values for the Different Substituents^a^

R=	α value
Phenol substituents	
*p-*H	0
*o-*H	0
*o-* ^t^Bu	–0.027
*p-* ^t^Bu	0.473
*o-*Me	0.292
*p-*Me	0.292
*o*-OMe	0.413
*p*-OMe	0.413
IMPY substituents	
H	0.380
Me	–0.223
Ph	–0.294
Py	0.134
CN	–0.015
*p*-CNC_6_H_4_	0.555
C_6_F_5_	0.555
*p*-(Me)_2_NC_6_H_4_	–0.446

a Phenol substituents are differentiated between *ortho* and *para* positions to the hydroxyl group.

It can be seen (Table 6) that compounds with a Ω value greater than 0.5 (taken arbitrarily as the lowest Ω value of the ESIPT-displaying group minus half the distance between this and the highest Ω value of the non-ESIPT group, indicating the mean point between the two groups) are those that undergo the ESIPT process. Additionally, the Ω values can be normalized relative to derivative **1**, which is considered to be the general reference.

3 $$\Omega^{\prime }=\Omega -\Omega_{\mathrm{compound}1}=\Omega -0.380$$

**Table 6:** Ω and Ω′ Parameters for All of the Derivatives Investigated

compound	Ω	Ω′	ESIPT
**1**	0.380	0.00	No
**2**	–0.223	–0.603	No
**3**	–0.294	–0.674	No
**4**	0.134	–0.246	No
**5**	0.827	0.446	Yes
**6**	0.223	–0.157	No
**7**	0.152	–0.223	No
**8**	0.581	0.201	Yes
**10**	0.718	0.338	Yes
**11**	–0.002	–0.383	No
**12**	0.00	–0.380	No
**13**	0.431	0.051	No
**14**	0.290	–0.091	No
**15**	1.00	0.620	Yes
**16**	1.00	0.620	Yes
**17**	0.107	–0.273	No
**18**	0.607	0.227	Yes
**19**	0.426	0.046	No
**20**	0.426	0.046	No
**21**	–0.002	–0.383	No
**22**	0.179	–0.201	No
**23**	–0.321	–0.701	No
**24**	0.569	0.189	Yes
**25**	0.793	0.413	Yes
**26**	0.793	0.413	Yes
**27**	1.207	0.827	Yes

The resulting parameter (Ω′) establishes a new scale, where derivatives with Ω′ > 0.12 (taken arbitrarily as the lowest Ω′ value of the ESIPT-displaying group minus half the distance between this and the highest Ω′ value of the non-ESIPT group, indicating the mean point between the two groups) are those that undergo proton transfer. Furthermore, positive Ω′ values indicate a higher likelihood of the system to present the ESIPT process with respect to parent compound **1**, while negative Ω′ values indicate the opposite.

Further future work, which is beyond the scope of this study, could expand the library of substituents and their position (including, for example, compound **9** as well or substituents in the *meta* position with respect to OH), improving the agreement obtained and refining the Ω′ or Ω′ thresholds needed to discriminate between compounds that undergo ESIPT and those that do not.

This analysis clearly indicates, as previously discussed, the necessity of a consistent but balanced push–pull effect involving electron-donating substituents on the hydrogen bond donor fragment (the phenol) and electron-withdrawing substituents on the acceptor fragment (the imidazo­[1,5-*a*]­pyridine). Electron-donating groups, such as methyl or *p*-(Me)_2_NC_6_H_4_ moieties located on the acceptor portion, do not facilitate effective charge redistribution, thereby preventing the occurrence of excited-state intramolecular proton transfer (ESIPT). Likewise, the cyano group (CN), despite its strong electron-withdrawing character, appears to inhibit the ESIPT process. This may be attributed to its limited ability to delocalize electron density, which may thus destabilize the keto-like excited-state form, ultimately hindering the ESIPT process.

With regard to substituents on the donor segment, analysis of the Ω′ values for derivatives **11** vs. **21**, **17** vs. **18**, **19** vs. **20**, and **22** vs. **23** suggests that a *para*-substituent relative to the hydroxyl group plays a more critical role than the one in the *ortho* position, at least for the *tert*-butyl substituent. Furthermore, the comparison between derivatives **17** and **19** highlights that a *tert*-butyl group, although more electron-donating than a methyl group, results in a lower propensity for ESIPT when positioned *ortho*. This is presumably due to its steric bulk, which likely hinders the planarity required for efficient intramolecular hydrogen bonding and, consequently, proton transfer in the excited state.

These observations indicate that electronic properties alone are not sufficient to account for the occurrence of the ESIPT phenomenon. Instead, a combination of electronic effects, steric hindrance, and geometric constraints must be considered, underscoring the intrinsic complexity of these systems.

To prove the soundness of the calculation, five more compounds, previously only analyzed *in silico*, were synthesized, namely compounds **10**, **11**, **14**, **15**, and **20** (Figures S17–S32 for NMR spectra). Compounds **11** (differently from what was previously reported),^52^ **14**, and **20**, as predicted from the model and also from the DFT functional used, did not show any ESIPT emission in dichloromethane solution (Figures S59–S60). On the contrary, compound **15**, which from the DFT calculation and Ω values was expected to undergo ESIPT, displayed a dual emission characterized by the standard blue emission and a low-intensity red-shifted one (Figure S61). Upon measurement of the fluorescence emission spectrum of **15** in the less polar hexane, this band increased in intensity, thus revealing the presence of the predicted ESIPT process. The ESIPT nature of this second emission was further confirmed upon measurement of the emission spectrum in methanol, leading to the complete disappearance of the red-shifted emission band (Figure S62). Compound **10**, instead, showed dual emission even in dichloromethane (Figure S63).

These additional experimental data confirmed the goodness of the mathematical model built to predict the presence of the ESIPT phenomenon in imidazo­[1,5-*a*]­pyridine-3-yl phenols, as well as the accuracy of the BLYP-D3­(BJ) functional in predicting the occurrence of the ESIPT phenomenon in these species.

It is essential to underscore that the proposed framework constitutes a mathematical model designed to predict the likelihood of ESIPT in imidazo­[1,5-*a*]­pyridin-3-yl phenol systems based on the nature and positioning of substituents. While this approach offers a valuable predictive tool that circumvents the need for resource-intensive quantum mechanical calculations, it remains a model subject to limitations. As famously stated by George Box, “All models are wrong, but some are useful”.^53^ It is therefore possible that additional factors, not currently accounted for, may influence the observed behavior, particularly in light of the fact that this class of compounds has historically been considered ESIPT-inactive.

Although the model could undoubtedly benefit from further refinement, such as the inclusion of additional substituents in varied positions, such developments fall beyond the scope of the present study.

### Ground-State Orbital Energy Parametrization

Using the same approach as that before, we then attempted to predict the frontier orbital energies in the ground state according to the substituents. This can be extremely useful in the design of optoelectronic devices, where the HOMO energy needs to be close to that of potential hole-transporting materials, while the LUMO energy should match that of possible electron-transporting materials. We then generated two additional linear systems (Tables S10 and S11), consisting of eqs 4 and 5.

4 $$E\left({\mathrm{HOMO}}_{S_{0}}^{\mathrm{DFT}}\right)=\sum_{i}C_{i}\cdot \alpha_{i}^{\mathrm{HOMO}}$$

5 $$E\left({\mathrm{LUMO}}_{S_{0}}^{\mathrm{DFT}}\right)=\sum_{i}C_{i}\cdot \alpha_{i}^{\mathrm{LUMO}}$$

where *E*(HOMO_*S*_0__^DFT^) and *E*(LUMO_*S*_0__^DFT^) are the HOMO and LUMO energies in the ground state computed via DFT, respectively; *C_i_* represents again the coefficients indicating the presence of the substituents, taking the value 1 if the substituent is present in the derivative and 0 if it is absent; and *α_i_* ^HOMO^ and *α_i_* ^LUMO^ are the values associated with the individual substituents, obtained by solving the system through linear regression for the HOMO and LUMO energy determination, respectively.

α^HOMO^ and α^LUMO^ were then used to calculate the predicted energies of these orbitals as follows

6 $$E\left({\mathrm{HOMO}}_{S_{0}}^{\mathrm{predicted}}\right)=\sum_{i}\alpha_{i}^{\mathrm{HOMO}}$$

7 $$E\left({\mathrm{LUMO}}_{S_{0}}^{\mathrm{predicted}}\right)=\sum_{i}\alpha_{i}^{\mathrm{LUMO}}$$

By using this model, it was possible to achieve excellent agreement with the observed data (with *R* ^2^ = 0.99) (Figure 13).

**Figure 13:**
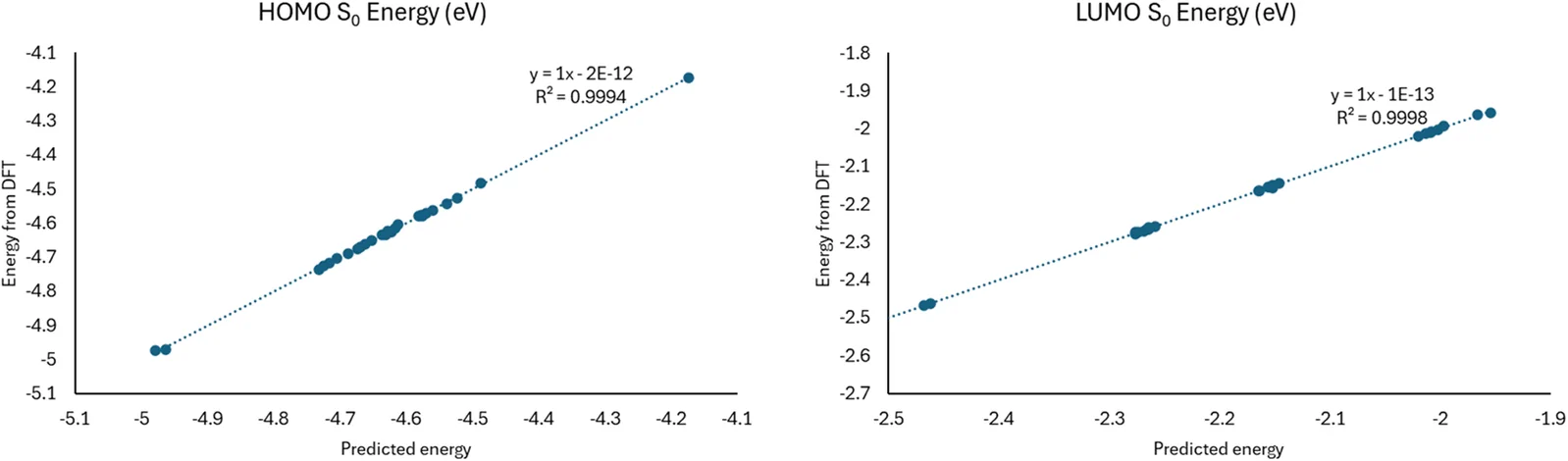
Accordance between the predicted and observed values of the frontier molecular orbitals’ energies.

Therefore, a new table can be obtained, listing the α^HOMO^ and α^LUMO^ values for each substituent (Table 7), with which it was possible to calculate *a priori* the HOMO and LUMO energies of these compounds in the ground state.

**Table 7:** Aα^HOMO^ and *α* ^LUMO^ Values for the Different Substituents^a^

R=	α^HOMO^	α^LUMO^
Phenol substituents		
*p-*H	0	0
*o-*H	0	0
*o-* ^t^Bu	0.036	0.012
*p-* ^t^Bu	0.053	–4 × 10^–4^
*o-*Me	0.031	0.010
*p-*Me	0.043	0.009
*o*-OMe	–0.015	–0.011
*p*-OMe	0.094	0.007
IMPY substituents		
H	–4.72	–2.01
Me	–4.58	–1.97
Ph	–4.61	–2.16
Py	–4.70	–2.28
CN	–5.05	–2.48
*p*-CNC_6_H_4_	–4.78	–2.58
C_6_F_5_	–4.81	–2.29
*p*-(Me)_2_NC_6_H_4_	–4.26	–2.02

a Phenol substituents are differentiated between *ortho* and *para* positions to the hydroxyl group.

## Experimental Section

### Materials and Methods

Elemental analyses were obtained with a PerkinElmer CHN Analyzer 2400 Series II. NMR spectra were recorded with an AVANCE 400 Bruker spectrometer at 400 MHz for ^1^H NMR and 100 MHz for ^13^C­{^1^H}. Chemical shifts are given as δ values in parts per million relative to residual solvent peaks as the internal reference. *J* values are given in Hz. The UV–vis, excitation, and emission spectra were measured using a fluorescence spectrometer (Edinburgh Instruments FS5) equipped with a 150 W continuous xenon lamp as a light source and an additional detector for transmittance and were corrected for the wavelength response of the instrument; lifetime measurements were performed on the same FS5 Edinburgh Instruments using an EPLED-320 (Edinburgh Instruments) as the pulsed source. Analysis of the lifetime decay curve and determination of absolute quantum yields were done using the Fluoracle Software package (Ver. 1.9.1), which runs the FS5 instrument. Absolute fluorescence quantum yields were determined on a Photon Technology International (PTI) QuantaMaster QM-40 spectrometer (Xe arc lamp, 70 W) with a PhotoMed GmbH K-Sphere Integrating Sphere (3.2 in. diameter). All of the chemicals were of reagent grade quality and were purchased commercially (AlfaAesar, Acros, TCI Chemicals, Fluorochem) and used as received.

### Synthesis

Compounds **1**,^40^ **2**, and **3**,^21^ **4**,^39^ **7**, and **8**,^41^ and **11** ^52^ were synthesized according to the literature.

**5:** 2-Picolylamine (0.50 g, 1 equiv) and 3,5-di-*tert*-butylsalicylic acid (1.3 g, 1.15 equiv) were dissolved in 25 mL of degassed butyl acetate. Then, 7.5 mL of T_3_P (50% m/m in ethyl acetate) was added to the mixture, which was left stirring at room temperature for 1 h, in which the precipitation of a white solid occurred. After this time, the reaction mixture was heated at 130 °C for 18 h, cooled to room temperature, and added slowly to 50 mL of aqueous sodium bicarbonate. The mixture was then extracted with dichloromethane (2 × 60 mL) and dried over anhydrous sodium sulfate. The solvent was removed under reduced pressure to give a yellow oil. The crude product was purified using column chromatography on silica gel using dichloromethane as the eluent to give the final product as a white solid.

Yield: 2.6 mg (68%). Anal. Calcd. (%) for C_21_H_26_N_2_O: C, 78.22; H, 8.13; N, 8.69. Found (%) C, 77.89; H, 8.06; N, 9.01. ^1^H NMR (400 MHz, CDCl_3_, 298 K, *J* [Hz]): δ = 11.57 (s, 1H), 8.43 (d, *J* = 7.3 Hz, 1H), 7.59 (d, *J* = 2.2 Hz, 1H), 7.55 (s, 1H), 7.50 (d, *J* = 9.1 Hz, 1H), 7.41 (d, *J* = 2.2 Hz, 1H), 6.79–6.75 (m, 1H), 6.66–6.61 (m, 1H), 1.52 (s, 9H), 1.40 (s, 9H). ^13^C NMR (101 MHz, CDCl_3_, 298 K): δ = 152.71, 140.54, 137.44, 137.29, 130.99, 124.70, 122.20, 119.58, 119.40, 119.17, 118.51, 113.72, 113.68, 35.50, 34.58, 31.80, 29.71.

**6**: 2-Acetylpyridine (1.0 g, 1 equiv), hydroxylammonium chloride (688 mg, 1.2 equiv), and potassium carbonate (3.4 g, 3 equiv) were added to 20 mL of degassed methanol. The mixture was left stirring at room temperature for 4 h; then, it was filtered, and the organic phase was collected and degassed again. At this point, zinc (2.7 g, powder, 5 equiv) and ammonium chloride (2.2 g, 5 equiv) were added to the reaction mixture and left stirring at room temperature overnight. The reaction mixture was then filtered, and the solvent was removed under reduced pressure. 20 mL of distilled water was added to the oil obtained, and the pH was adjusted to 13 using a NaOH 10 M solution. The mixture was extracted with dichloromethane (3 × 15 mL), the organic phases were collected and dried over anhydrous sodium sulfate. and the solvent was removed under reduced pressure affording 232 mg of 1-(pyridin-2-yl)­ethan-1-amine as a yellow oil, which was used without further purification.

1-(Pyridin-2-yl)­ethan-1-amine (0.23 g, 1 equiv) and 3,5-di-*tert*-butylsalicylic acid (0.55 mg, 1.15 equiv) were dissolved in 10 mL of degassed butyl acetate. Then, 7.5 mL of T_3_P (50% m/m in ethyl acetate) was added to the mixture, which was left stirring at room temperature for 1 h, in which the precipitation of a white solid occurred. After that time, the reaction mixture was heated at 130 °C for 18 h, cooled to room temperature, and added slowly to 50 mL of aqueous sodium bicarbonate. The mixture was then extracted with dichloromethane (2 × 50 mL) and dried over anhydrous sodium sulfate. The solvent was removed under reduced pressure to give a yellow oil. The crude product was purified using column chromatography on silica gel using dichloromethane as an eluent to give the final product as a white solid.

Yield: 0.49 mg (77%). Anal. Calcd. (%) for C_22_H_28_N_2_O: C, 78.53; H, 8.39; N, 8.33. Found (%) C, 78.31; H, 8.60; N, 8.74. ^1^H NMR (400 MHz, CDCl_3_, 298 K, *J* [Hz]): δ = 11.87 (s, 1H), 8.35 (d, *J* = 7.3 Hz, 1H), 7.57 (d, *J* = 1.9 Hz, 1H), 7.42–7.38 (m, 2H), 6.72–6.61 (m, 1H), 6.56 (t, *J* = 6.8 Hz, 1H), 2.56 (s, 3H), 1.52 (s, 9H), 1.39 (s, 9H). ^13^C NMR (101 MHz, CDCl_3_, 298 K): δ = 152.61, 140.42, 137.29, 135.53, 127.25, 127.01, 124.37, 121.96, 119.31, 118.71, 117.60, 113.77, 113.49, 35.47, 34.57, 31.81, 29.74, 12.41.

**9**: 2-Hydroxy-6-methylbenzaldehyde (1.5 g, 1 equiv) and ammonium acetate (2.12 g, 2.5 equiv) were added to 20 mL of degassed acetic acid. The resulting solution turned dark yellow upon mixing. Subsequently, 2-acetyl-3-methylpyridine (0.74 g, 0.5 equiv) was added to the reaction mixture, which was stirred at room temperature for 7 days. After this period, 50 mL of distilled water was added to the reaction mixture, followed by extraction with dichloromethane (3 × 70 mL). The combined organic phases were washed with saturated sodium bicarbonate solution and dried over sodium sulfate, and the solvent was removed under reduced pressure. This yielded a brown oil, which was triturated with hexane to afford the final product as a brown solid. Yield: 0.41 mg (30%). Anal. Calcd. (%) for C_22_H_28_N_2_O: C, 76.16; H, 6.39; N, 11.10. Found (%) C, 76.76; H, 6.21; N, 10.98. ^1^H NMR (400 MHz, CDCl_3_, 298 K, *J* [Hz]): δ = 7.28 (d, *J* = 9.2 Hz, 1H), 7.10 (d, *J* = 7.2 Hz, 1H), 6.96 (d, *J* = 7.6 Hz, 1H), 6.78 (t, *J* = 7.5 Hz, 1H), 6.64 (m, 1H), 6.32 (d, *J* = 6.4 Hz, 1H), 2.32 (s, 3H), 2.25 (s, 6H). ^13^C NMR (101 MHz, CDCl_3_, 298 K): δ = 153.75, 134.25, 133.55, 131.55, 129.03, 128.43, 127.47, 126.44, 118.55, 118.37, 118.29, 116.14, 114.59, 21.36, 16.53, 12.04.

**10**: A mixture of di­(2-pyridyl) ketone (0.32 g, 1 equiv), 3,5-dimethyl salicylaldehyde (460 μL, 2 equiv), and ammonium acetate (0.67 g, 5 equiv) in glacial acetic acid (20 mL) was refluxed for 6 h. Then, the mixture was poured into 150 mL of water and extracted with CH_2_Cl_2_ (200 mL). The organic phase was washed with a saturated aqueous solution of NaHCO_3_ and dried with Na_2_SO_4_, and the suspension was filtered. The solvent was evaporated under a vacuum, and the crude product was triturated with hexane. Yield 0.37 g (68%). Anal. Calcd. (%) for C_20_H_17_N_3_O: C, 80.23; H, 5.77; N, 8.91. Found (%) C, 79.98; H, 5.76; N, 9.01. ^1^H NMR (400 MHz, CDCl_3_, 298 K, *J* [Hz]): δ = 11.68 (s, 1H), 8.76 (d, *J* = 9.2 Hz, 1H), 8.63 (m, 1H), 8.52 (d, *J* = 7.2 Hz, 1H), 8.12 (d, *J* = 7.9 Hz, 1H), 7.71 (t, *J* = 7.7 Hz, 1H), 7.40 (s, 1H), 7.13–7.06 (m, 1H), 7.03 (s, 1H), 6.97–6.94 (m, 1H), 6.73 (t, *J* = 6.7 Hz, 1H), 2.36 (s, 6H). ^13^C NMR (101 MHz, CDCl_3_, 298 K): δ = 154.14, 152.37, 149.19, 136.43, 136.17, 132.33, 129.62, 128.53, 127.69, 126.69, 122.67, 122.56, 122.29, 121.66, 120.87, 119.89, 114.69, 113.08, 20.95, 16.50.

**14**: A mixture of 2-benzoyl pyridine (0.16 g, 1 equiv), 3,5-dimethyl salicylaldehyde (230 μL, 2 equiv), and ammonium acetate (0.33 g, 5 equiv) in glacial acetic acid (15 mL) was refluxed for 6 h. Then, the mixture was poured into 150 mL of water and extracted with CH_2_Cl_2_ (200 mL). The organic phase was washed with a saturated aqueous solution of NaHCO_3_ and dried with Na_2_SO_4_, and the suspension was filtered. The solvent was evaporated under a vacuum, and the crude product was triturated with hexane. Yield 0.17 g (62%). Anal. Calcd. (%) for C_21_H_18_N_2_O: C, 76.16; H, 6.39; N, 11.10. Found (%) C, 76.76; H, 6.21; N, 10.98. ^1^H NMR (400 MHz, CDCl_3_, 298 K, *J* [Hz]): δ = 11.88 (s, 1H), 8.52 (d, *J* = 7.3 Hz, 1H), 7.89 (m, 3H), 7.47 (t, 7.6 Hz, 2H), 7.41 (s, 1H), 7.33 (t, *J* = 7.3 Hz, 1H), 7.03 (s, 1H), 6.87–6.84 (m, 1H), 6.69 (t, J = 6.8 Hz, 1H), 2.36 (s, 6H). ^13^C NMR (101 MHz, CDCl_3_, 298 K): δ = 152.50, 136.29, 133.97, 132.30, 129.89, 128.94, 127.64, 127.08, 127.01, 126.88, 126.80, 122.91, 122.39, 120.48, 119.55, 114.09, 113.13, 20.98, 16.55.

**15**: 4-Picolinoylbenzonitrile was prepared according to the literature.^54^ A mixture of 4-picolinoylbenzonitrile (1.20 g, 1 equiv), 3,5-diterbutyl salicylaldehyde (2.70 g, 2 equiv), and ammonium acetate (2.22 g, 5 equiv) in glacial acetic acid (30 mL) was refluxed for 6 h. Then, the mixture was poured into 150 mL of water and extracted with CH_2_Cl_2_ (200 mL). The organic phase was washed with a saturated aqueous solution of NaHCO_3_ and dried with Na_2_SO_4_, and the suspension was filtered. The solvent was evaporated under a vacuum, and the crude product was triturated with hexane. Yield 0.14 g (51%). Anal. Calcd. (%) for C_28_H_29_N_3_O: C, 79.40; H, 6.90; N, 9.92. Found (%) C, 79.45; H, 6.69; N, 10.31. ^1^H NMR (400 MHz, CDCl_3_, 298 K, *J* [Hz]): δ = 11.21 (s, 1H), 8.49 (d, *J* = 7.3 Hz, 1H), 8.04 (d, *J* = 8.4 Hz, 2H), 7.90 (d, *J* = 9.3 Hz, 1H), 7.75 (d, *J* = 8.4 Hz, 2H), 7.57 (d, *J* = 2.2 Hz, 1H), 7.44 (d, *J* = 2.2 Hz, 1H), 7.02–6.98 (m, 1H), 6.76 (t, *J* = 6.7 Hz, 1H), 1.51 (s, 9H), 1.38 (s, 9H). ^13^C NMR (101 MHz, CDCl_3_, 298 K): δ = 152.77, 141.05, 132.81, 128.43, 126.71, 125.67, 123.17, 122.41, 120.04, 119.29, 119.01, 114.49, 112.95, 35.56, 34.65, 31.77, 29.71.

**20**: A mixture of di­(2-pyridyl) ketone (0.20 g, 1 equiv), 2-hydroxy-5-methylbenzaldehyde (0.28 g, 2 equiv), and ammonium acetate (0.41 g, 5 equiv) in glacial acetic acid (20 mL) was refluxed for 6 h. Then, the mixture was poured into 150 mL of water and extracted with CH_2_Cl_2_ (200 mL). The organic phase was washed with a saturated aqueous solution of NaHCO_3_ and dried with Na_2_SO_4_, and the suspension was filtered. The solvent was evaporated under a vacuum, and the crude product was triturated with hexane. Yield 0.21 g (64%). Anal. Calcd. (%) for C_19_H_15_N_3_O: C, 75.73; H, 5.02; N, 13.94. Found (%) C, 75.50; H, 5.14; N, 13.68. ^1^H NMR (400 MHz, CDCl_3_, 298 K, *J* [Hz]): δ = 11.47 (s, 1H), 8.79 (d, *J* = 9.2 Hz, 1H), 8.64 (d, *J* = 4.1 Hz, 1H), 8.54 (d, *J* = 7.2 Hz, 1H), 8.12 (d, *J* = 8.0 Hz, 1H), 7.74 (t, *J* = 7.7 Hz, 1H), 7.57 (s, 1H), 7.18–7.05 (m, 3H), 7.01–6.97 (m, 1H), 6.77 (t, *J* = 6.7 Hz, 1H), 2.39 (s, 3H). ^13^C NMR (101 MHz, CDCl_3_, 298 K): δ = 154.26, 154.10, 149.23, 136.53, 135.77, 130.97, 129.70, 128.77, 128.37, 124.94, 122.53, 122.39, 121.77, 120.97, 119.94, 117.74, 114.88, 113.86, 21.00.

### Computational Details

All calculations were carried out at the density functional (DFT) level of theory with the ADF2021.102 program package.^55^ The BLYP-D3­(BJ) functional was employed for all of the calculations. Frequency analyses were performed for all optimized structures to establish the nature of the stationary points. TD-DFT implemented in the ADF package was used to determine the excitation energies: the 30 lowest singlet–singlet excitations were calculated using optimized geometries. For geometry optimizations, C, N, and O atoms were described through TZ2P basis sets [triple-ξ Slater-type orbitals (STOs) plus two polarization functions], while H-atoms were described through TZP [triple-ξ Slater-type orbitals (STOs) plus one polarization function]. The corresponding augmented basis set was employed in TD-DFT calculations.^56^ Restricted formalism, no-frozen-core approximation (all-electron), and no-symmetry constraints were used in all calculations. Solvent effects (CH_2_Cl_2_) were simulated by employing the conductor-like continuum solvent model (COSMO)^57^ as implemented in the ADF suite.

PES scans have been performed by elongating from 0.8 to 2.2 Å the oxygen···hydrogen bond length of the OH group toward the nitrogen atom in the imidazole ring (10 steps, 0.14 Å each). In the ground state, for every step, the molecular geometry has been optimized maintaining the O–H length fixed. The same has been done for the excited states, optimizing the S_1_ using the TD-DFT method.

Resolution of the linear systems has been performed using wxMaxima^58^ (23.05.1 version).

### X-ray Characterization

A crystal of **7**, slow evaporation of saturated dichloromethane (technical grade), was mounted on a STOE STADIVARI Eulerian 4-circle diffractometer (Darmstadt, Germany) equipped with a Pilatus300 K detector (Dectris, Baden, Switzerland), using Cu Kα radiation (λ = 1.54186 Å). The structure was solved and refined by direct methods using the OLEX2 platform.^59^ The H-atoms were included in calculated positions and treated as riding atoms, while the non-H-atoms were refined anisotropically, using weighted full-matrix least-squares on *F* ^2^. Crystallographic details are summarized in Table S1. Figure 3 was drawn with Mercury.^60^

## Conclusions

In conclusion, different species belonging to the imidazo­[1,5-*a*]­pyridin-3-yl phenol family were synthesized, and their photophysical properties were thoroughly investigated. In solution, all compounds exhibited blue fluorescence. For two of them, an additional lower energy emission band was observed, attributed to the ESIPT (excited-state intramolecular proton transfer) phenomenon, so far considered inaccessible for imidazo­[1,5-*a*]­pyridine derivatives.^47^ In the solid state and in thin films, these compounds consistently displayed blue fluorescence.

Theoretical calculations were conducted to rationalize the occurrence of the ESIPT process, which was found to be dependent on the nature of the substituents. Specifically, the presence of electron-donating groups on the phenolic ring and electron-withdrawing groups on the imidazopyridine core appeared to be essential. Moreover, a delicate balance between electron donation, electron acceptance, and π-delocalization is required for the ESIPT process to take place.

Finally, a numerical descriptor, termed Ω′, was developed to empirically distinguish between ESIPT-active and ESIPT-inactive species. These findings may pave the way for more targeted studies and rational design of ESIPT-based fluorophores with enhanced fluorescence quantum yields. Furthermore, the same approach could be used to calculate *a priori* the frontier orbitals’ energies of these species, paving as well the way for a faster intelligent design of materials for optoelectronic applications.

## Supplementary Material





## Notes

NMR spectra, further photophysical and computational analysis, crystallographic data for compound **7** and mathematical models (PDF)
Cartesian coordinates (*xyz*) of optimized structures (XLSX)
