# Supplementary material for: Should I Stay or Should I Go? Applying a Combined Experimental and Theoretical Approach to Predict the Excited-State Intramolecular Proton Transfer (ESIPT) in Imidazo[1,5‑a]pyridine-3-yl Phenols
Source: Precis Chem. 2026 May 6;4(10):1660–77. doi: 10.1021/prechem.6c00034 (PMC13715319; doi:10.1021/prechem.6c00034)
Supplement: Supplementary file 1 [file pc6c00034_si_001.pdf]

Supporting Information

Should I stay or should I go? Applying a combined experimental and theoretical approach to predict the excited state intramolecular proton transfer (ESIPT) in imidazo[1,5-*a*]pyridine-3-yl phenols

Anita Cinco,[a,b] Gioele Colombo,[a*] Stefano Brenna,[a*] Chiara Vola,[a] Bruno Therrien,[c] G. Attilio Ardizzoia[a]

[a] Dipartimento di Scienza e Alta Tecnologia, Università degli Studi dell'Insubria and CIRCC, Via Valleggio, 9 - 22100 Como, Italy and CIRCC

[b] University School for Advanced Studies IUSS Pavia, Italy

[c] Institute of Chemistry, University of Neuchatel, Avenue de Bellevaux 51, CH-2000 Neuchatel, Switzerland

Corresponding author: Gioele Colombo (University of Insubria), Stefano Brenna (University of Insubria)

Tel.: +39-(0)31-2386476; Fax: +39-(0)31-2386119; e-mail address: gioele.colombo@uninsubria.it; stefano.brenna@uninsubria.it

Contents

**Figures S1-S32**	^{1}H, COSY, HSQC and ^{13}C NMR spectra of compounds **1**, **5**, **6**, **9**, **10**, **14**, **15** and **20**.
**Figure S33**	Crystal structure of compound **7** in which are highlighted the angles between moieties.
**Figure S34**	CIE 1931 chromaticity plot for compounds **1**-**8** recorded in solution.
**Figure S35**	Normalized absorption, emission and excitation spectra of compounds **8** recorded in different solvents.
**Figure S36**	Relative emission intensities of the enol and keto forms of compound **8** measured in different solvents.
**Figure S37**	Normalized emission spectra of compound **8** measured in dichloromethane solution before and after deuteration.

Figure S38 Relative emission intensities of the enol and keto forms of compound **8** measured in dichloromethane/methanol solution.
Figure S39 Normalized absorption, emission and excitation spectra of compound **9** measured in dichloromethane.
Figure S40 Normalized emission spectra of compound **2** measure in dichloromethane increasing the viscosity of the solution with eicosane.
Figure S41 Emission spectra of compounds **5** and **8** measured in 2-methyl tetrahydrofuran at different temperatures.
Figure S42 Deconvolution of the emission spectra of compounds **5** and **8** measured in 2-methyl tetrahydrofuran solution.
Figure S43 Ratio between the phenol and the keto emission intensities of compounds **5** and **8** measured in 2-methyl tetrahydrofuran solution at different temperatures.
Figure S44 Emission spectra of compounds **2** and **7** measured in 2-methyl tetrahydrofuran at different temperatures.
Figure S45 Emission spectra of compounds **7** and **8** measured in dichloromethane (5x 10^{-5} M) at low temperatures.
Figure S46 Normalized emission spectra of compounds **8** measured in dichloromethane (5x10^{-5} M) at low temperatures and relative emission intensities of the enol (blue, normalized) and keto (orange) forms of compound **8** measured at low temperatures.
Figure S47 Normalized excitation and emission spectra of compounds **1**-**8** recorded in solid state.
Figure S48 CIE 1931 chromaticity plot of compounds **1**-**8** recorded in solid state.
Figure S49 Normalized excitation and emission spectra of compounds **1**-**8** recorded in thin film using PMMA as matrix.
Figure S50 Normalized excitation and emission spectra of compounds **1**-**8** recorded in thin film using PS as matrix.
Figure S51 CIE 1931 chromaticity plot of compounds **1**-**8** recorded in thin film using PMMA as matrix.
Figure S52 CIE 1931 chromaticity plot of compounds **1**-**8** recorded in thin film using PS as matrix.
Figure S53 Frontier orbitals of compounds **1**-**8**.
Figure S54 Experimental and calculated UV-vis spectra of compounds **1**-**8** in dichloromethane solution.
Figure S55 PES Scan curves for the enol and keto forms of compound **7** computed both in the ground and first excited state.
Figure S56 Computed energies of compounds **1-8** calculated in their different virtual and real configurations.
Figure S57 Electron Density Difference Maps (EDDM) for the lowest energy singlet electronic transition computed for compounds **1**-**8**.

Figure S58 Electron Density Difference Maps (EDDM) for the lowest energy singlet electronic transition computed for compounds **5** and **8** in their ketonic form.
Figure S59 Normalized absorption and emission spectra of compound **11** recorded in dichloromethane.
Figure S60 Normalized absorption and emission spectra of compound **14** recorded in dichloromethane.
Figure S61 Normalized absorption and emission spectra of compound **20** recorded in dichloromethane.
Figure S62 Normalized emission spectra of compound **15** recorded in different media.
Figure S63 Normalized absorption and emission spectra of compound **10** recorded in dichloromethane.

Table S1 Crystallographic and structure refinement parameters for compound **7**.
Table S2 Photophysical data of compound **8** recorded in different solvents.
Table S3 Lifetime decays comparison between those measured at the standard emission and those measured at the ESIPT emission, in dichloromethane solution of compounds **5** and **8**.
Table S4 Comparison between experimental and calculated emission wavelengths of compounds **1**-**8**.
Table S5 Photophysical data of compounds **1**-**8** recorded in thin film with polymeric matrixes.
Table S6 Total energies for ground and first excited singlet state, computed for compounds **1**-**24**.
Table S7 Calculated hydrogen bond parameters of compounds **1**-**8**.
Table S8 Most important contribution of single orbital transitions to lower energy absorption of compounds **1**-**8**.
Table S9 System of linear equations written in matrix form for the calculation of Ω.
Table S10 System of linear equations written in matrix form for the calculation of HOMO energies in the ground state.
Table S11 System of linear equations written in matrix form for the calculation of LUMO energies in the ground state.

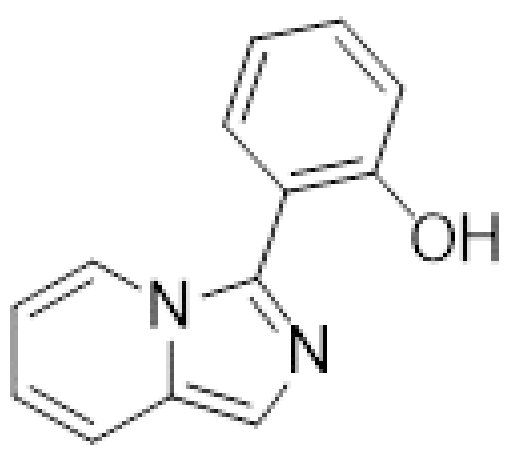

1: ^{1}H NMR (400 MHz, $CDCl_3$, 298 K, *J* [Hz]): δ = 8.50 (d, *J* = 7.3 Hz, 1H), 7.75 (d, *J* = 7.7 Hz, 1H), 7.55 (s, 1H), 7.53 (d, *J* = 9.16 Hz, 1H), 7.29 (t, *J* = 7.26 Hz, 1H), 7.16 (d, *J* = 8.1 Hz, 1H), 6.99 (t, *J* = 7.5 Hz, 1H), 6.80 (m, 1H), 6.67 (t, *J* = 6.8 Hz, 1H). ^{13}C NMR (101 MHz, $CDCl_3$, 298 K): δ = 156.58, 136.21, 131.06, 129.93, 124.33, 122.34, 119.68, 119.25, 119.08, 118.51, 117.92, 114.36, 114.07.

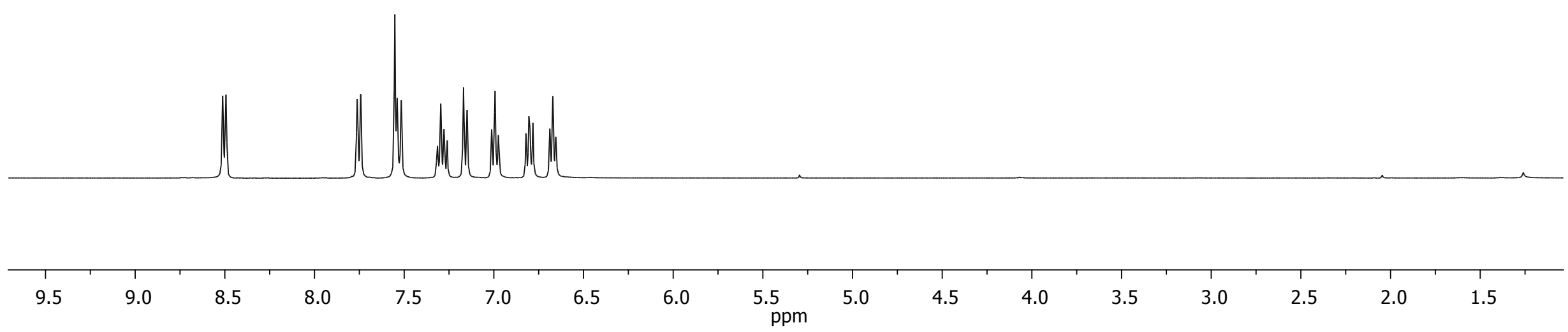

Figure S1. ^{1}H NMR spectrum ($CDCl_3$, 298 K) of **1**.

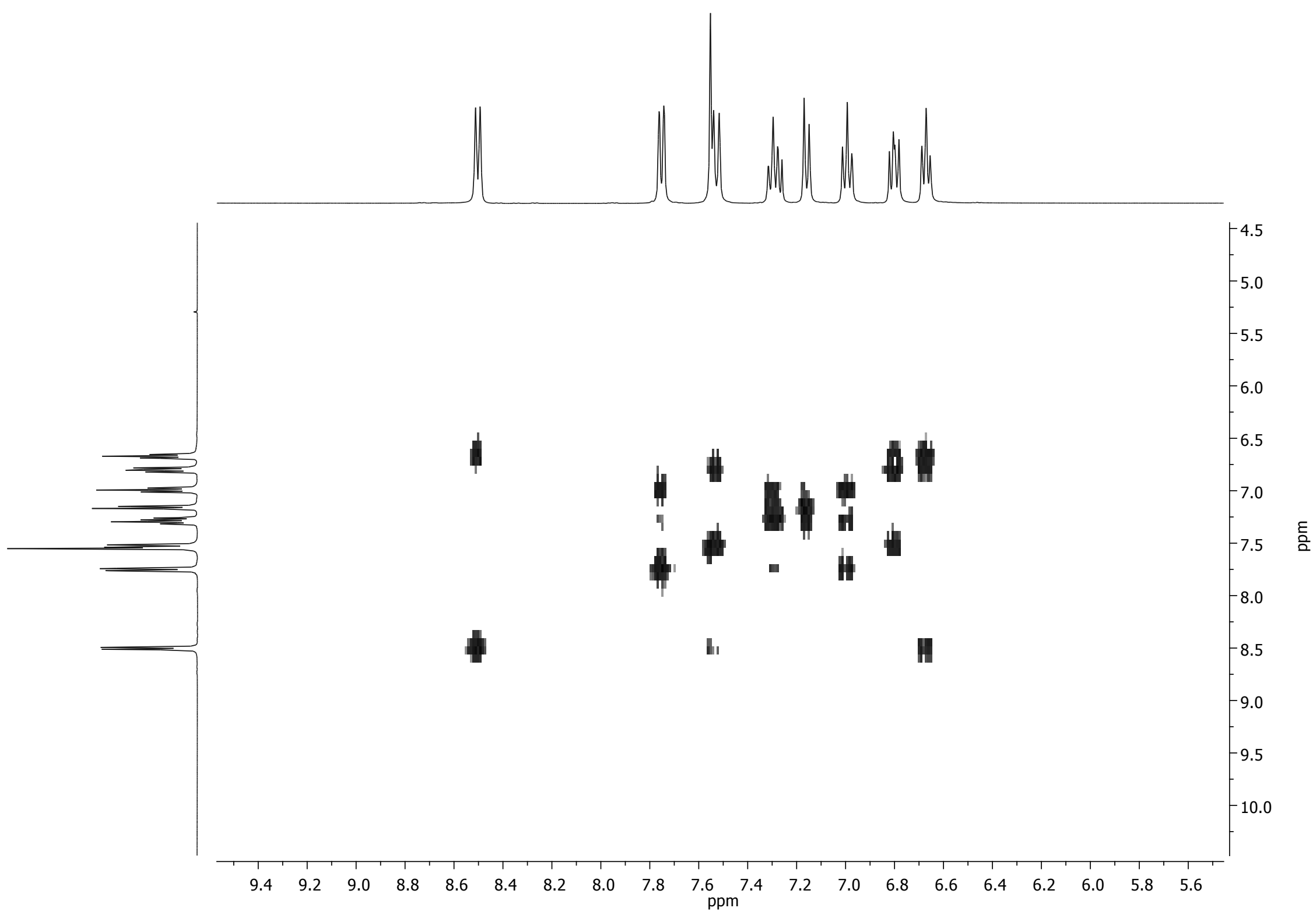

Figure S2. COSY-NMR spectrum ($CDCl_3$, 298 K) of **1**.

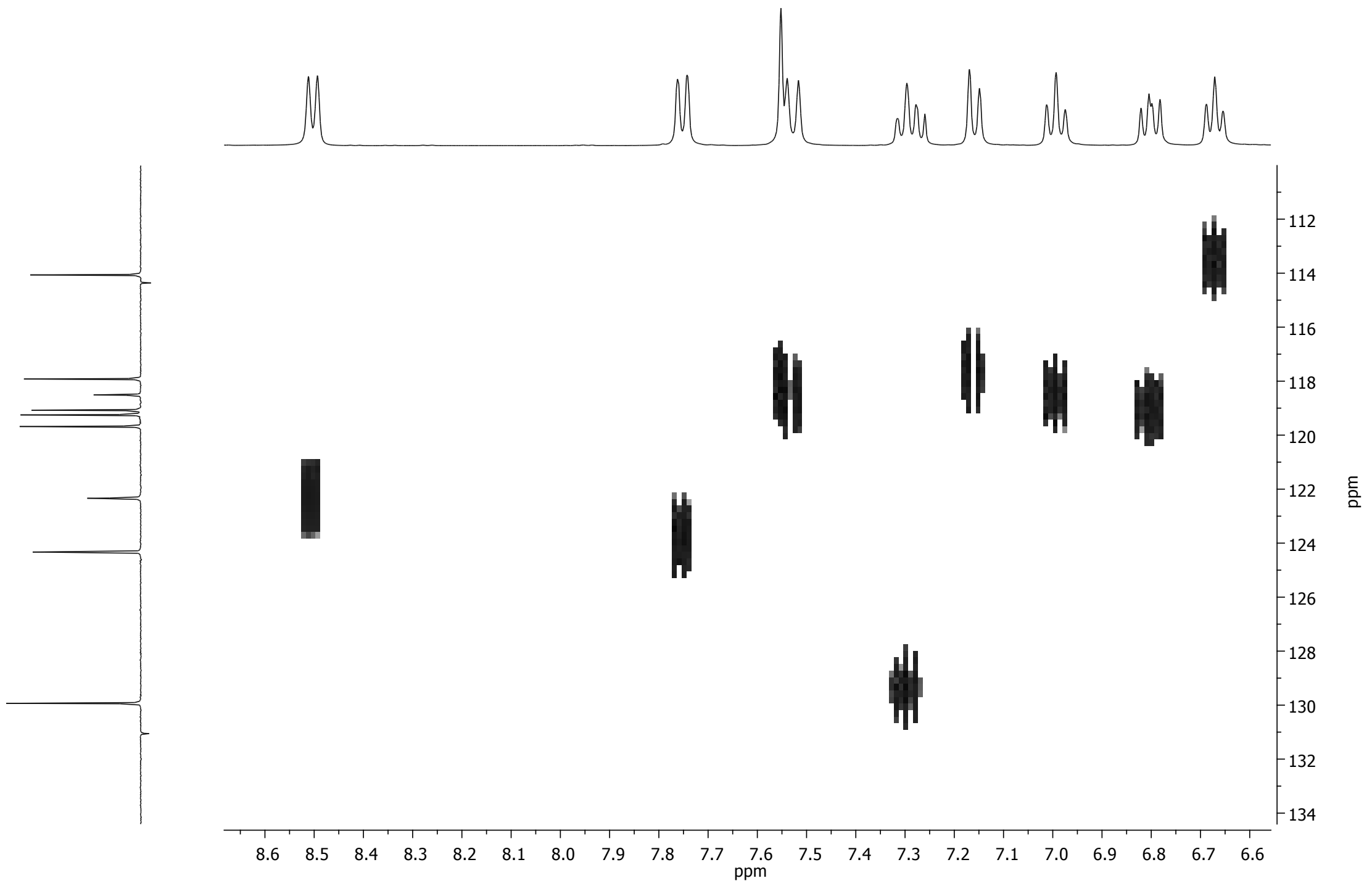

Figure S3. HSQC-NMR spectrum ($CDCl_3$, 298 K) of **1**.

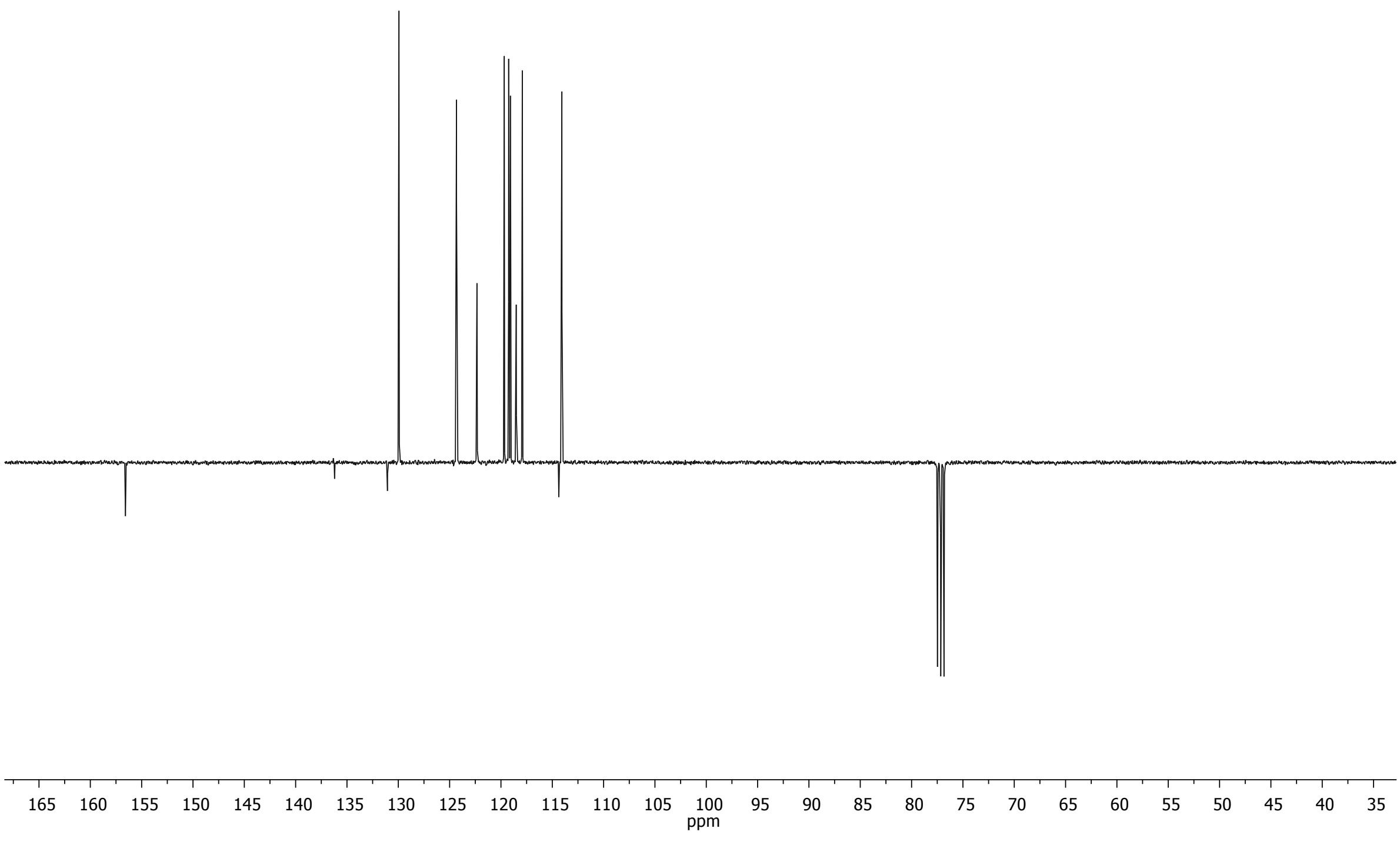

Figure S4. ^{13}C NMR spectrum ($CDCl_3$, 298 K) of **1**.

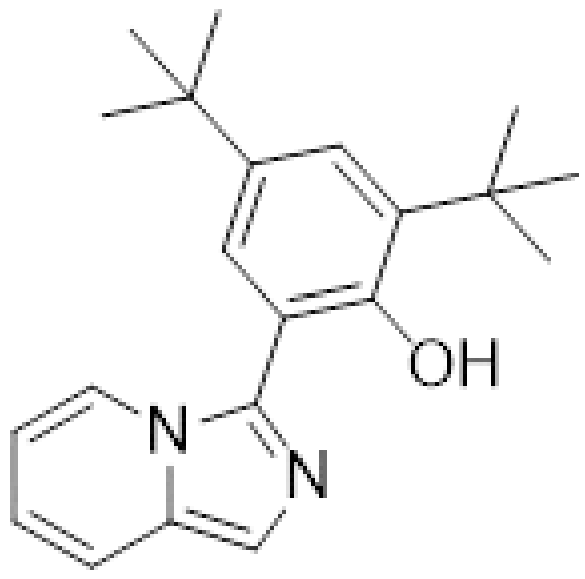

5: ^{1}H NMR (400 MHz, $CDCl_3$, 298 K, *J* [Hz]): δ = 11.57 (s, 1H), 8.43 (d, *J* = 7.3 Hz, 1H), 7.59 (d, *J* = 2.2 Hz, 1H), 7.55 (s, 1H), 7.50 (d, *J* = 9.1 Hz, 1H), 7.41 (d, *J* = 2.2 Hz, 1H), 6.79 – 6.75 (m, 1H), 6.66 – 6.61 (m, 1H), 1.52 (s, 9H), 1.40 (s, 9H). ^{13}C NMR (101 MHz, $CDCl_3$, 298 K): δ = 152.71, 140.54, 137.44, 137.29, 130.99, 124.70, 122.20, 119.58, 119.40, 119.17, 118.51, 113.72, 113.68, 35.50, 34.58, 31.80, 29.71.

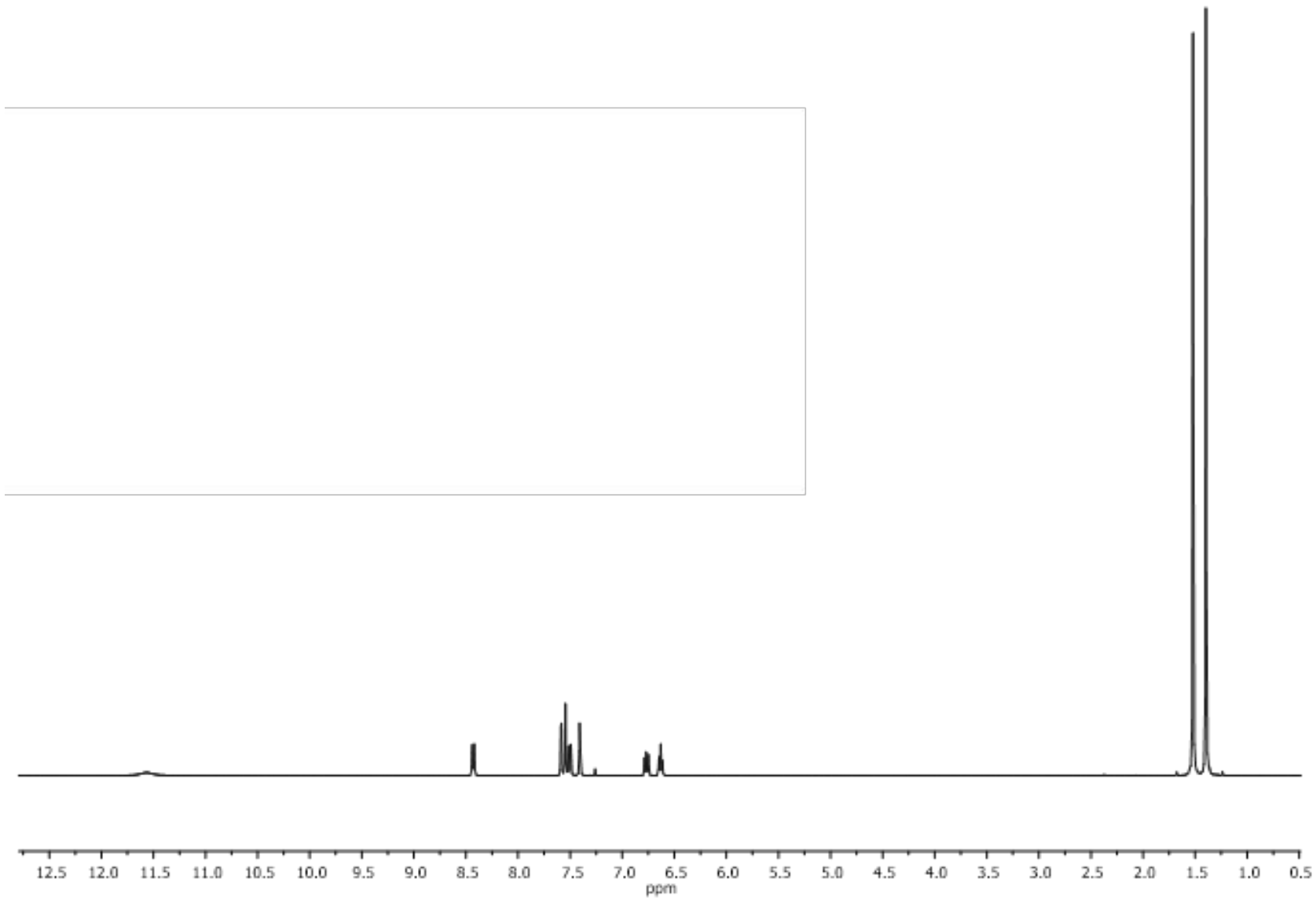

Figure S5. ^{1}H NMR spectrum ($CDCl_3$, 298 K) of **5**.

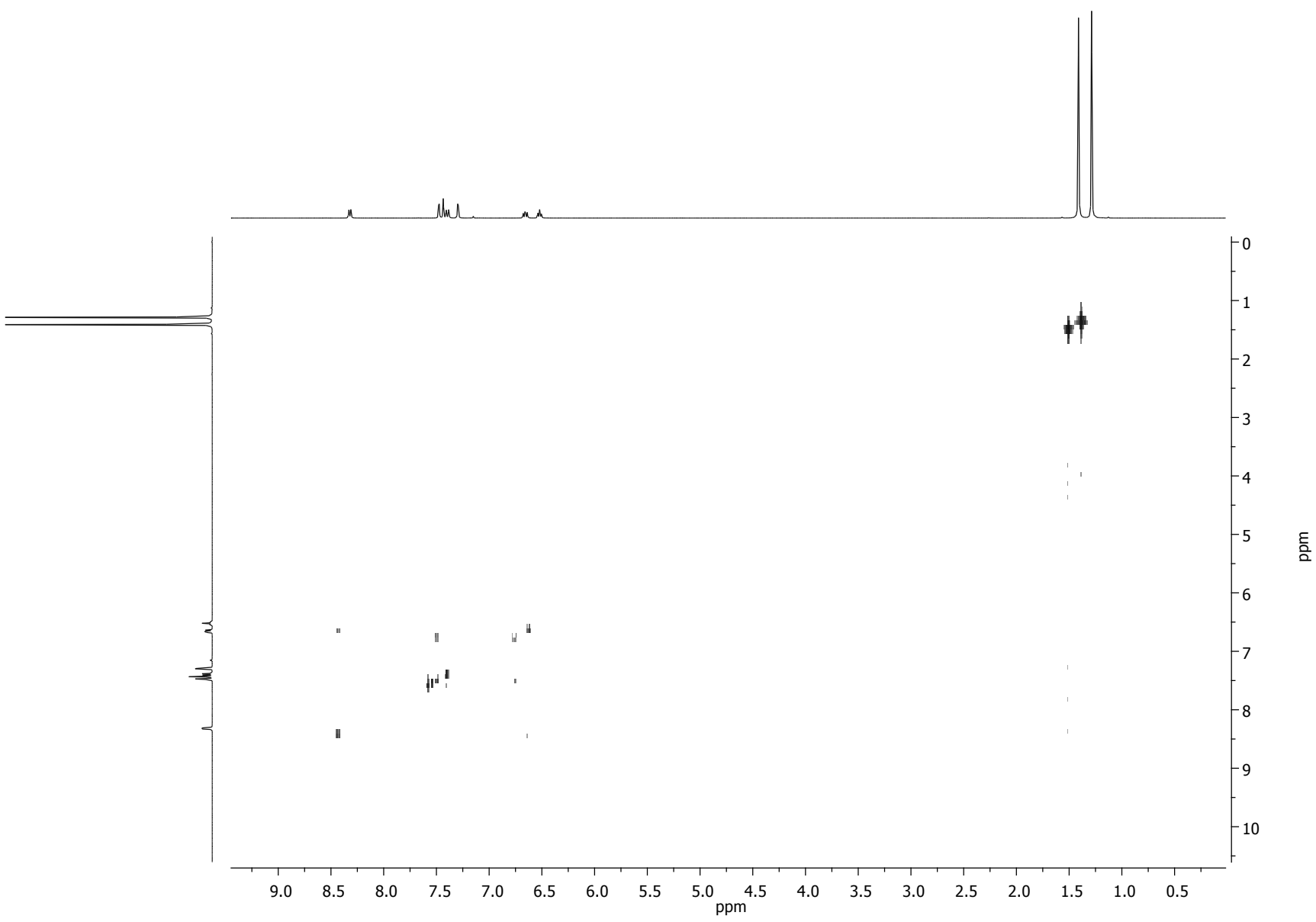

Figure S6. COSY-NMR spectrum ($CDCl_3$, 298 K) of **5**.

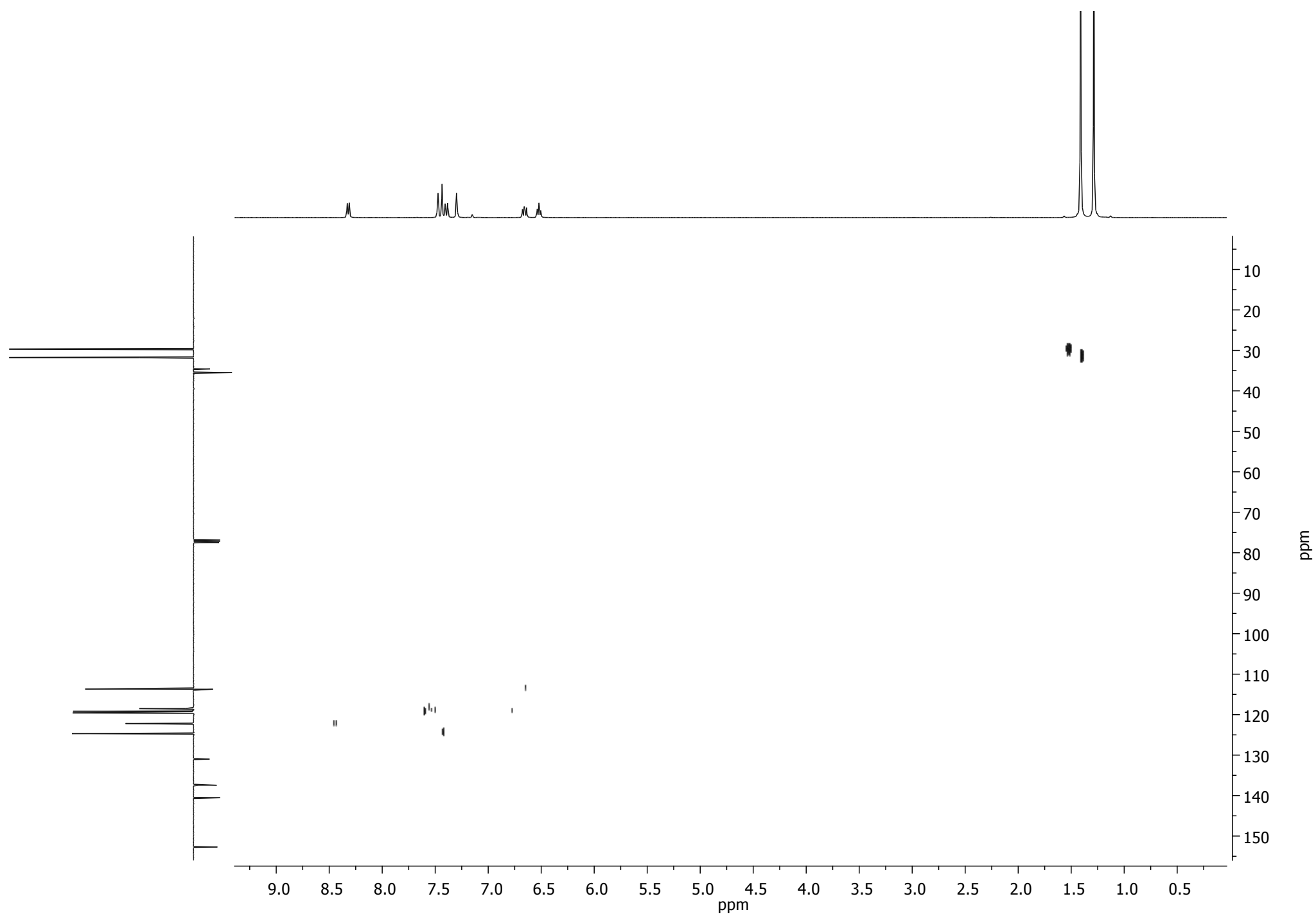

Figure S7. HSQC-NMR spectrum ($CDCl_3$, 298 K) of **5**.

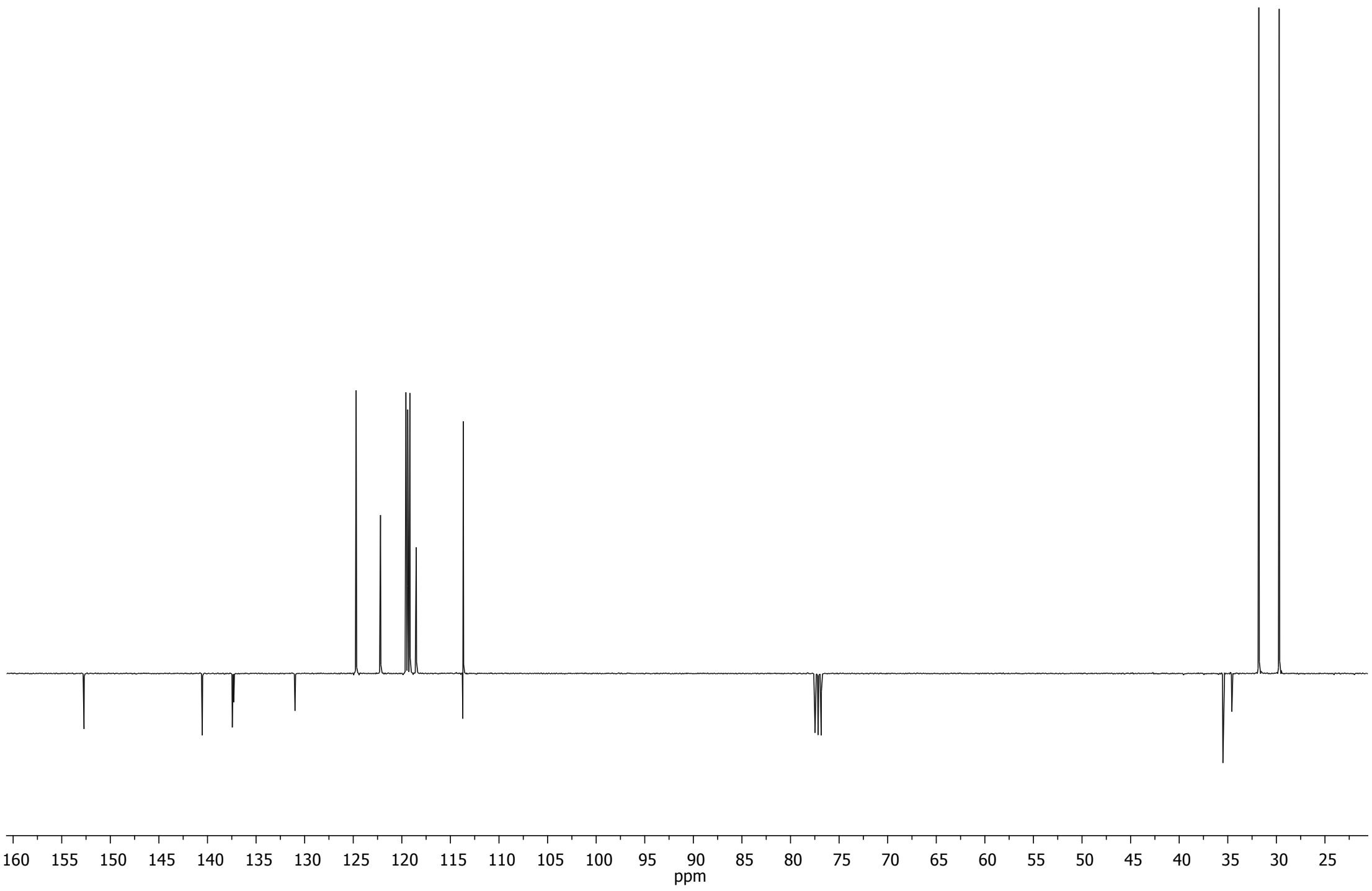

Figure S8. ^{13}C NMR spectrum ($CDCl_3$, 298 K) of **5**.

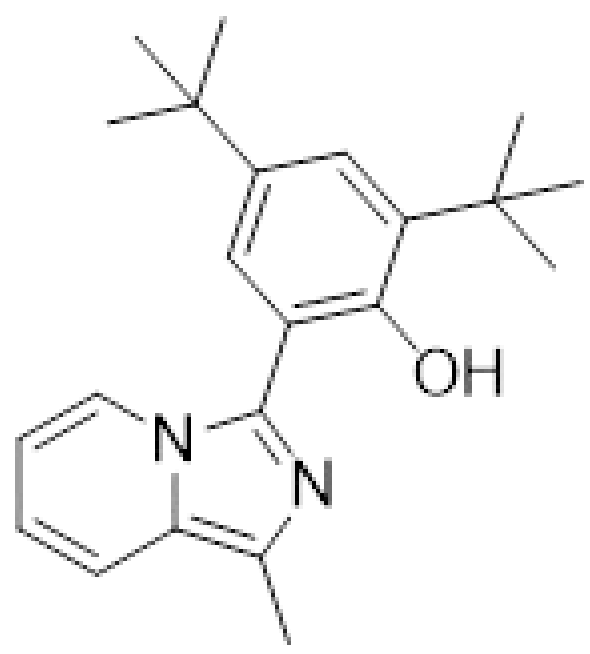

6: ^{1}H NMR (400 MHz, $CDCl_3$, 298 K, *J* [Hz]): δ = 11.87 (s, 1H), 8.35 (d, *J* = 7.3 Hz, 1H), 7.57 (d, *J* = 1.9 Hz, 1H), 7.42 – 7.38 (m, 2H), 6.72 – 6.61 (m, 1H), 6.56 (t, *J* = 6.8 Hz, 1H), 2.56 (s, 3H), 1.52 (s, 9H), 1.39 (s, 9H). ^{13}C NMR (101 MHz, $CDCl_3$, 298 K): δ = 152.61, 140.42, 137.29, 135.53, 127.25, 127.01, 124.37, 121.96, 119.31, 118.71, 117.60, 113.77, 113.49, 35.47, 34.57, 31.81, 29.74, 12.41.

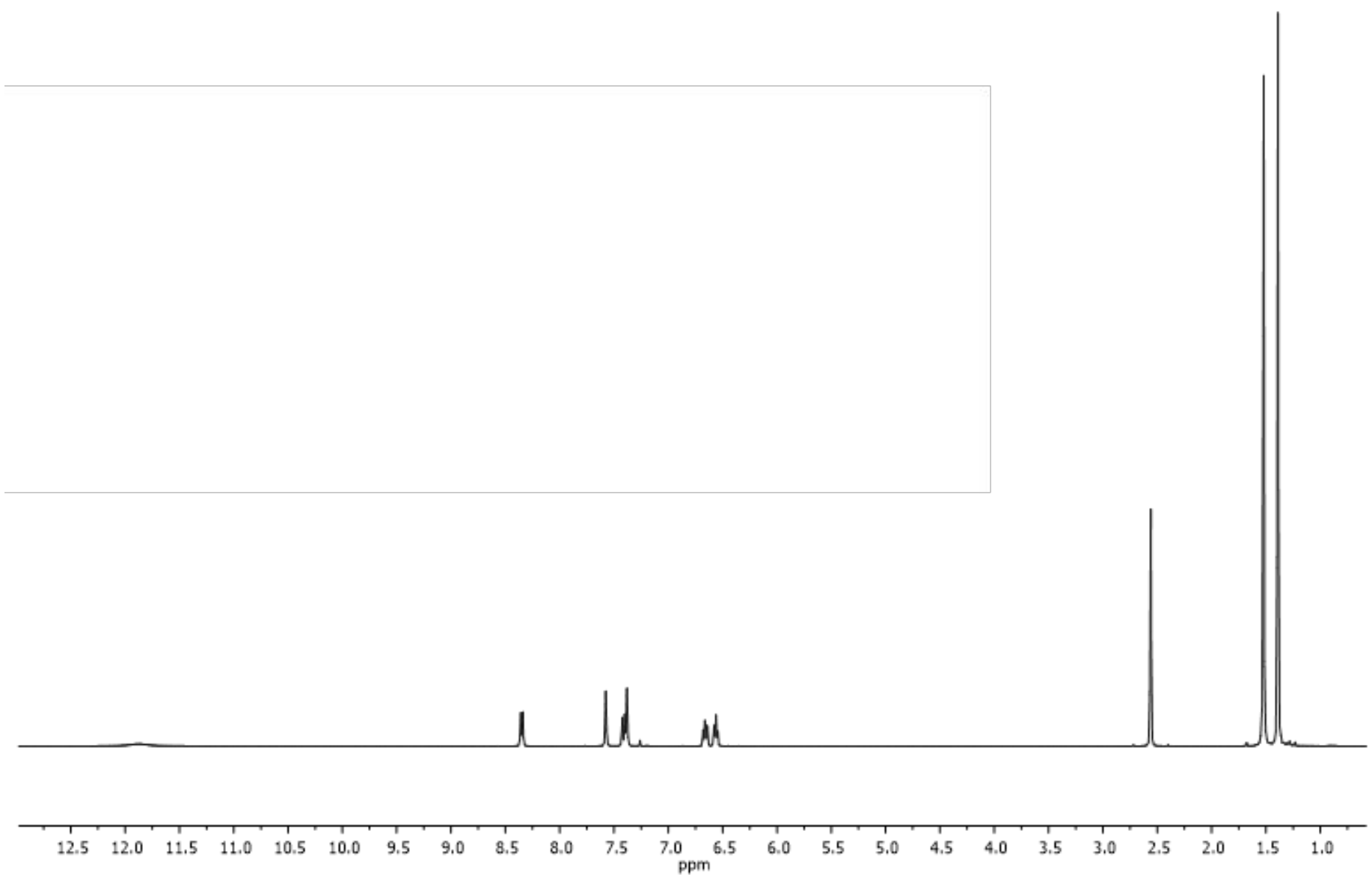

Figure S9. ^{1}H NMR spectrum ($CDCl_3$, 298 K) of **6**.

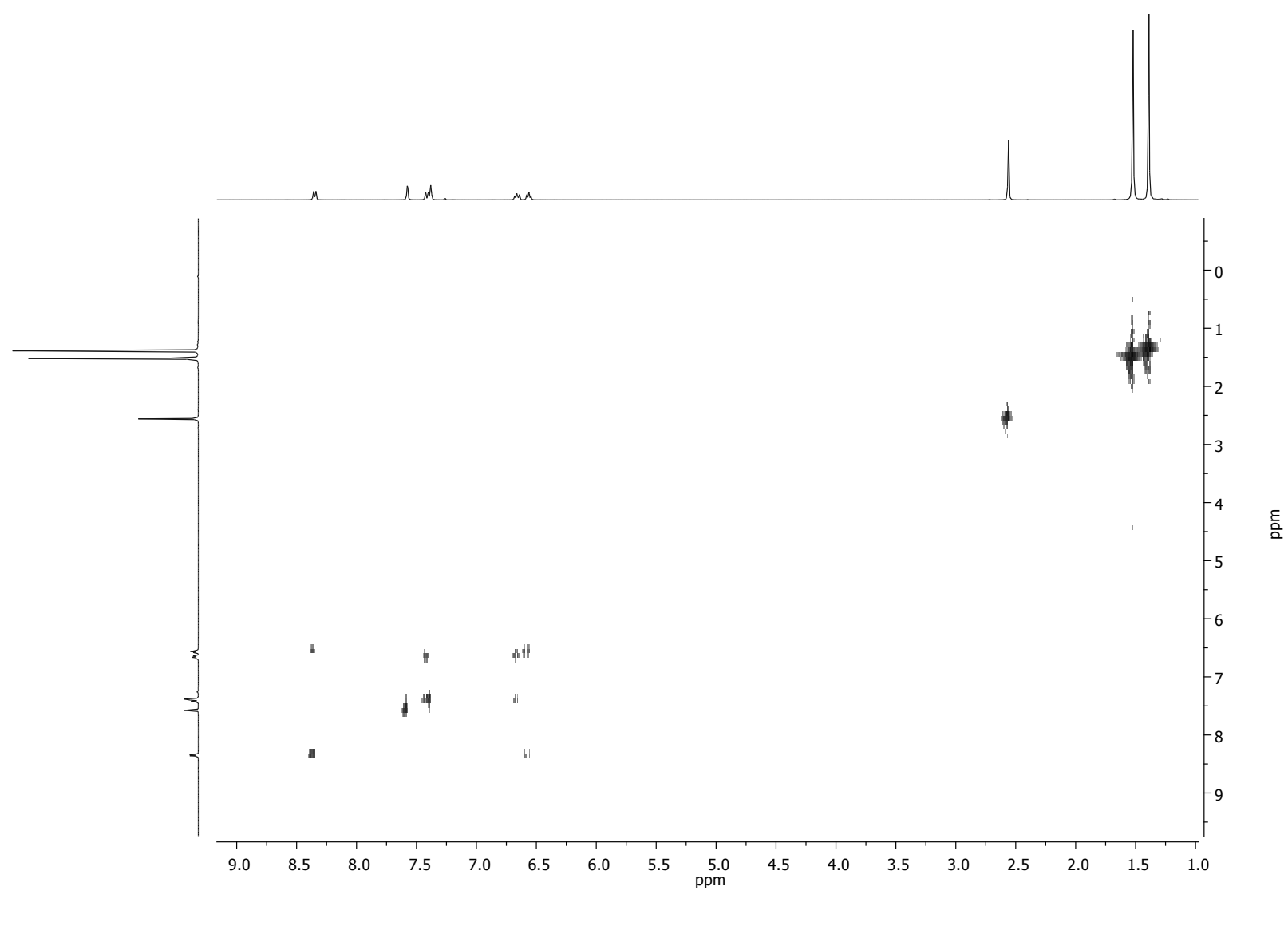

Figure S10. COSY-NMR spectrum ($CDCl_3$, 298 K) of **6**

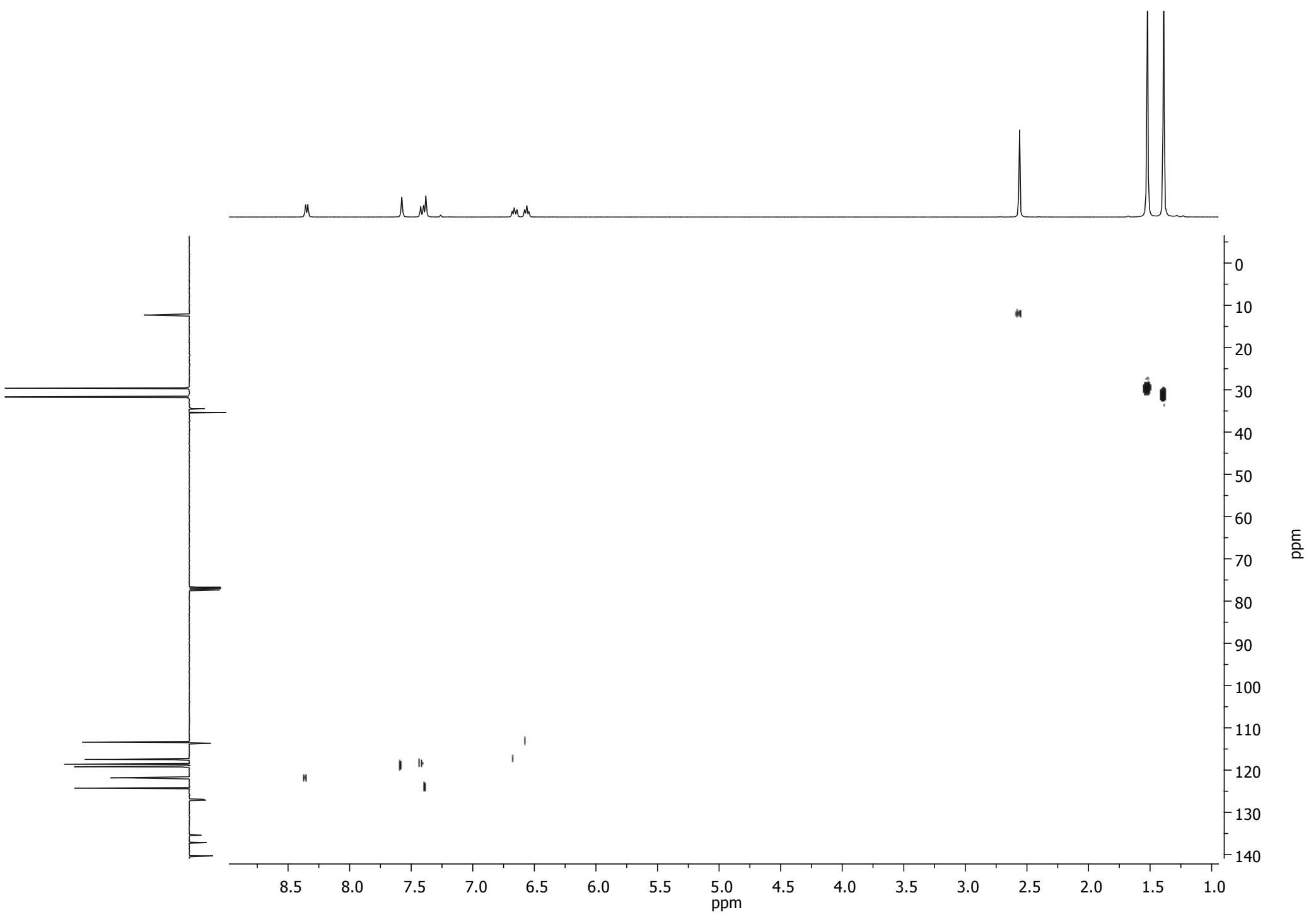

Figure S11. HSQC-NMR spectrum ($CDCl_3$, 298 K) of **6**.

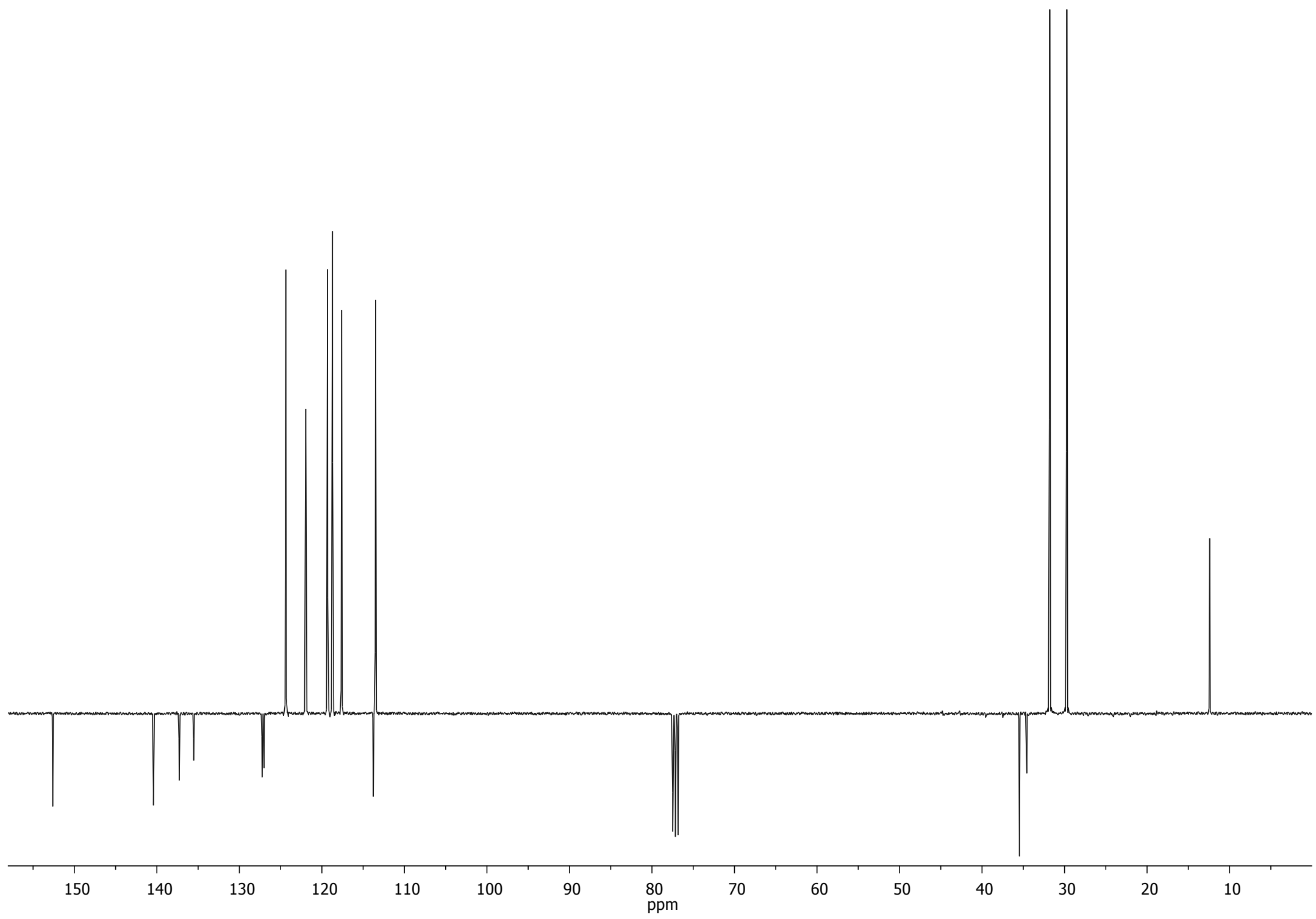

Figure S12. ^{13}C NMR spectrum ($CDCl_3$, 298 K) of **6**.

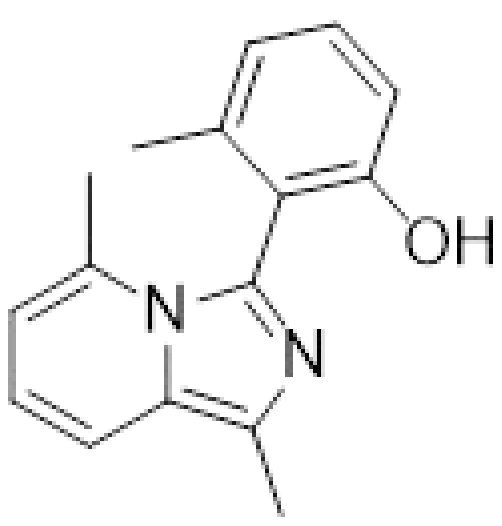

9: ^{1}H NMR (400 MHz, $CDCl_3$, 298 K, *J* [Hz]): δ = 7.28 (d, *J* = 9.2 Hz, 1H), 7.10 (d, *J* = 7.2 Hz, 1H), 6.96 (d, *J* = 7.6 Hz, 1H), 6.78 (t, *J* = 7.5 Hz, 1H), 6.64 (m, 1H), 6.32 (d, *J* = 6.4 Hz, 1H), 2.32 (s, 3H), 2.25 (s, 6H). ^{13}C NMR (101 MHz, $CDCl_3$, 298 K): δ = 153.75, 134.25, 133.55, 131.55, 129.03, 128.43, 127.47, 126.44, 118.55, 118.37, 118.29, 116.14, 114.59, 21.36, 16.53, 12.04.

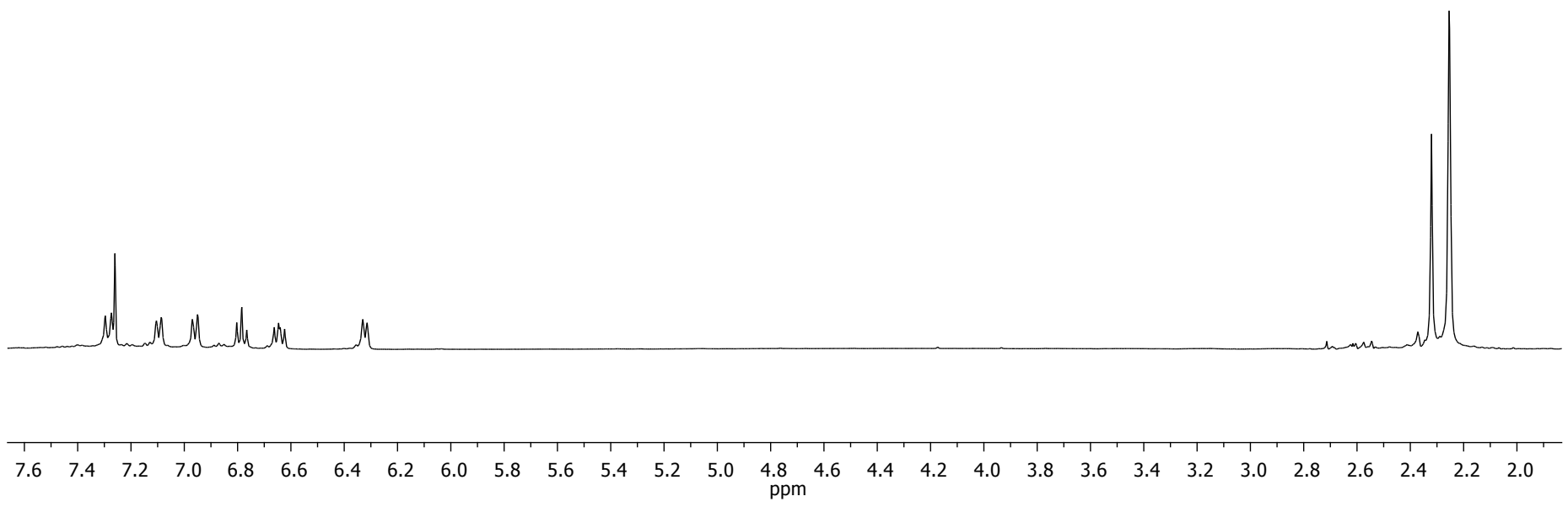

Figure S13. ^{1}H NMR spectrum ($CDCl_3$, 298 K) of **9**.

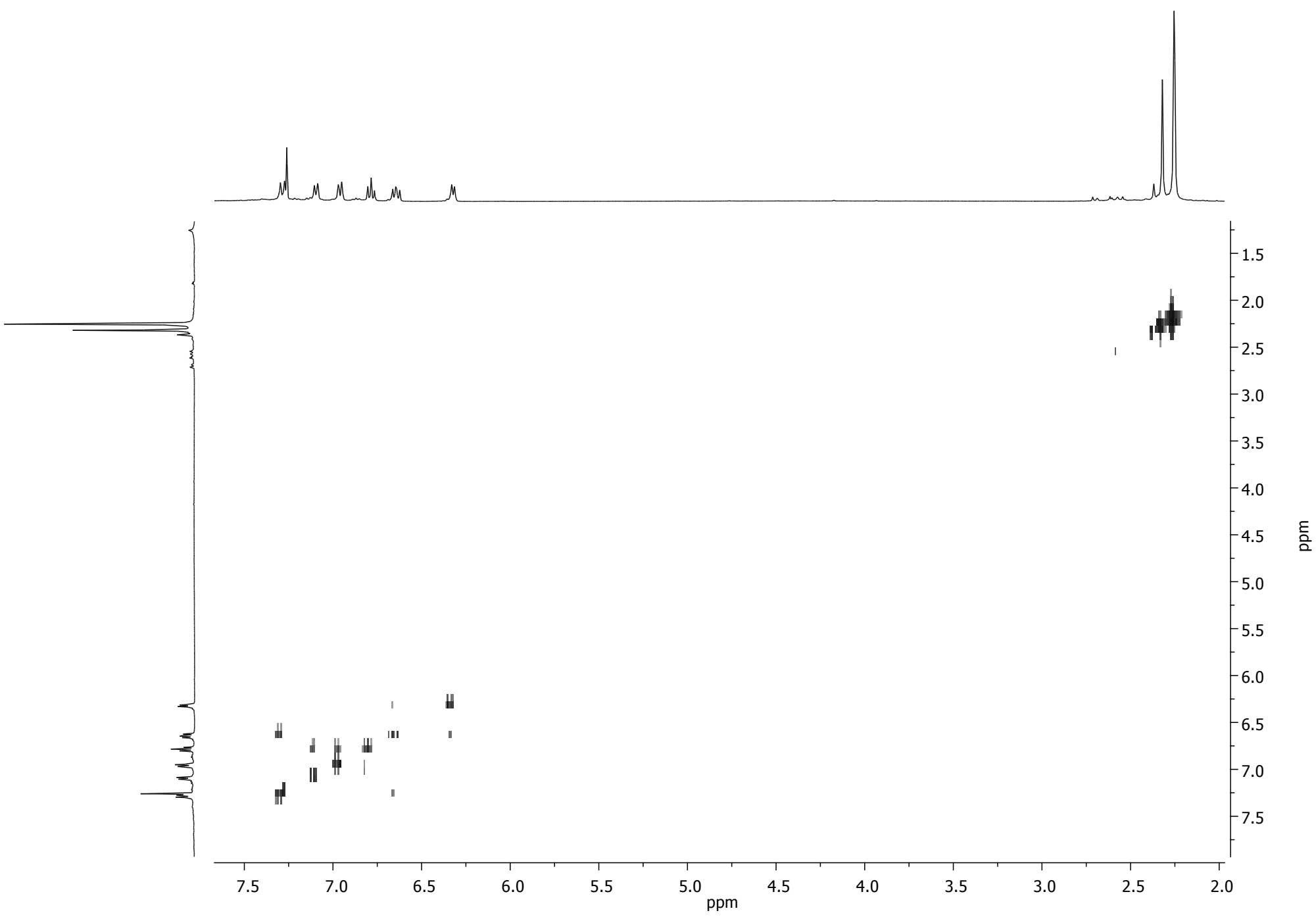

Figure S14. COSY-NMR spectrum ($CDCl_3$, 298 K) of **9.**

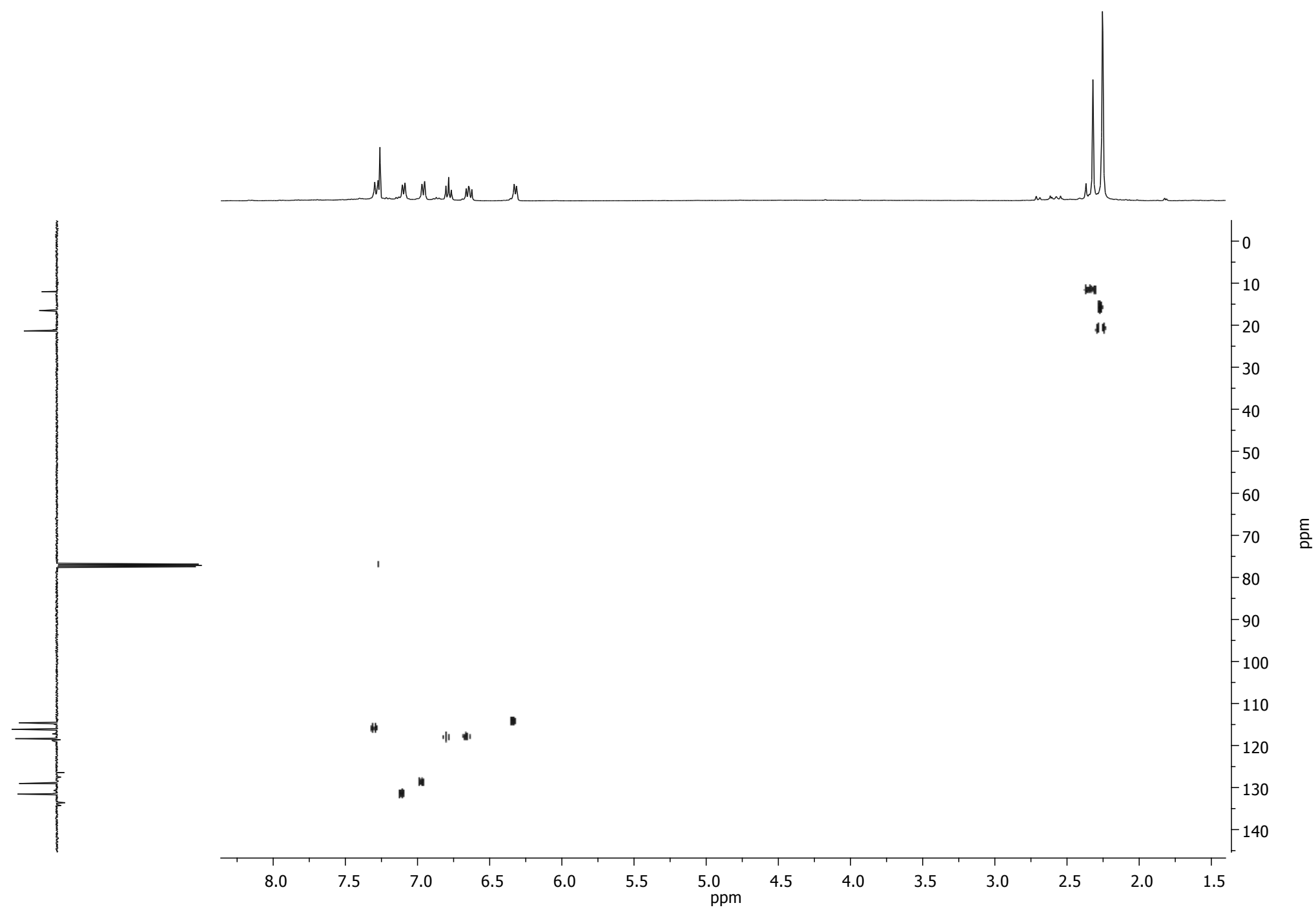

Figure S15. HSQC-NMR spectrum ($CDCl_3$, 298 K) of **9.**

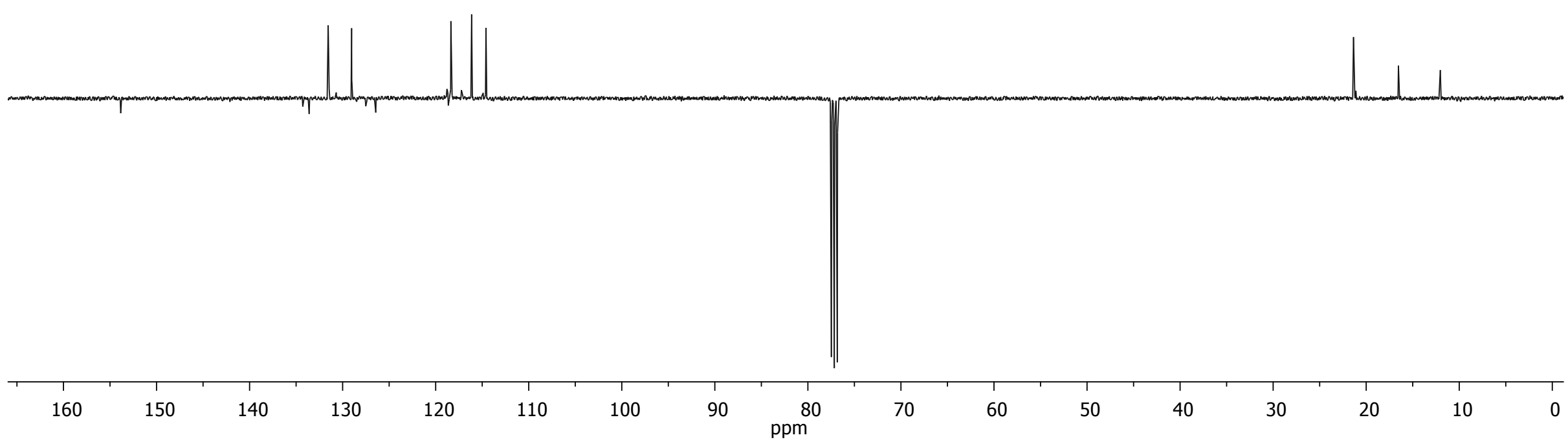

Figure S16. ^{13}C NMR spectrum ($CDCl_3$, 298 K) of **9**.

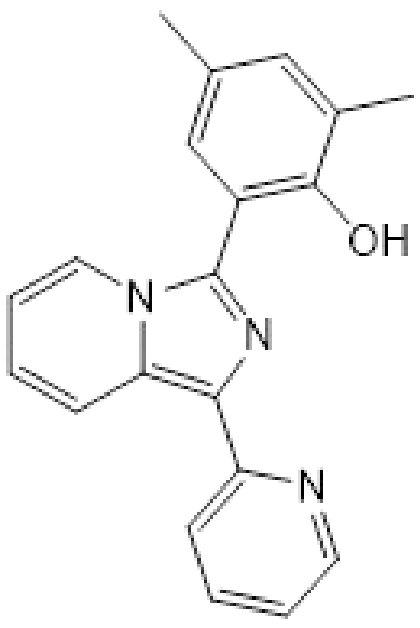

10: ^{1}H NMR (400 MHz, $CDCl_3$, 298 K, *J* [Hz]): δ = 11.68 (s, 1H), 8.76 (d, *J* = 9.2 Hz, 1H), 8.63 (m, 1H), 8.52 (d, *J* = 7.2 Hz, 1H), 8.12 (d, *J* = 7.9 Hz, 1H), 7.71 (t, *J* = 7.7 Hz, 1H), 7.40 (s, 1H), 7.13 – 7.06 (m, 1H), 7.03 (s, 1H), 6.97 – 6.94 (m, 1H), 6.73 (t, *J* = 6.7 Hz, 1H), 2.36 (s, 6H). ^{13}C NMR (101 MHz, $CDCl_3$, 298 K): δ = 154.14, 152.37, 149.19, 136.43, 136.17, 132.33, 129.62, 128.53, 127.69, 126.69, 122.67, 122.56, 122.29, 121.66, 120.87, 119.89, 114.69, 113.08, 20.95, 16.50.

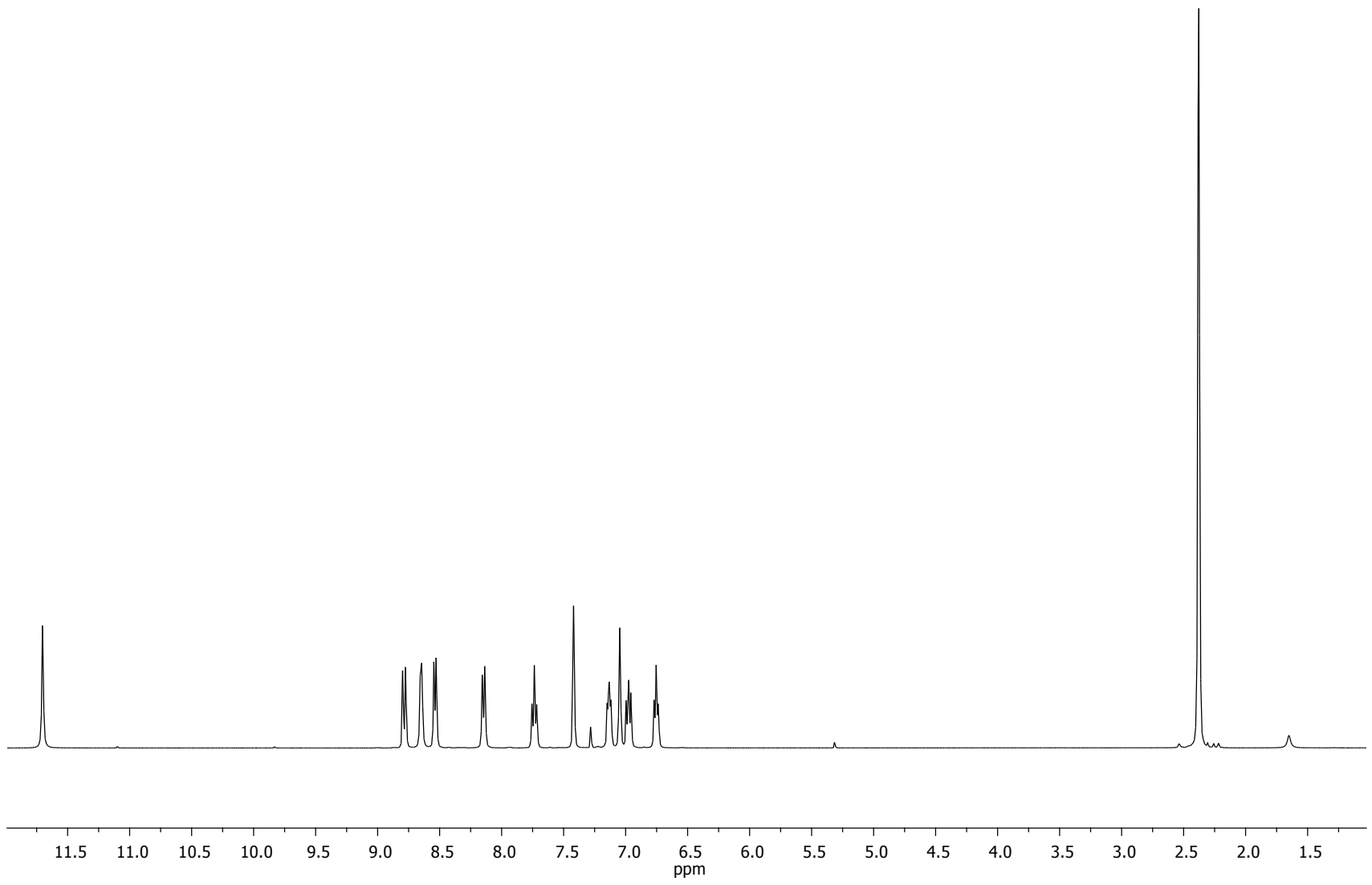

Figure S17. ^{1}H NMR spectrum ($CDCl_3$, 298 K) of **10**.

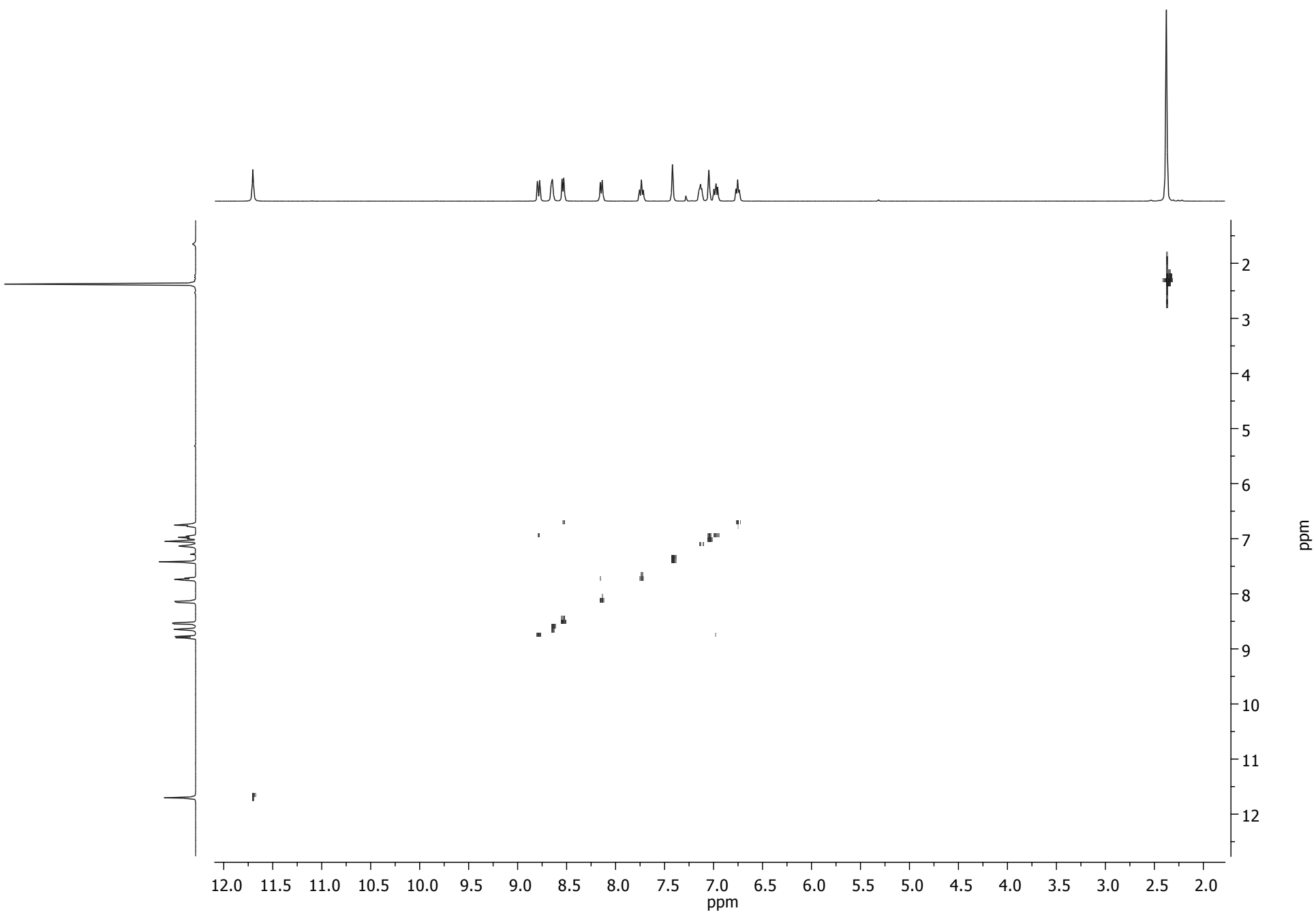

Figure S18. COSY-NMR spectrum ($CDCl_3$, 298 K) of **10.**

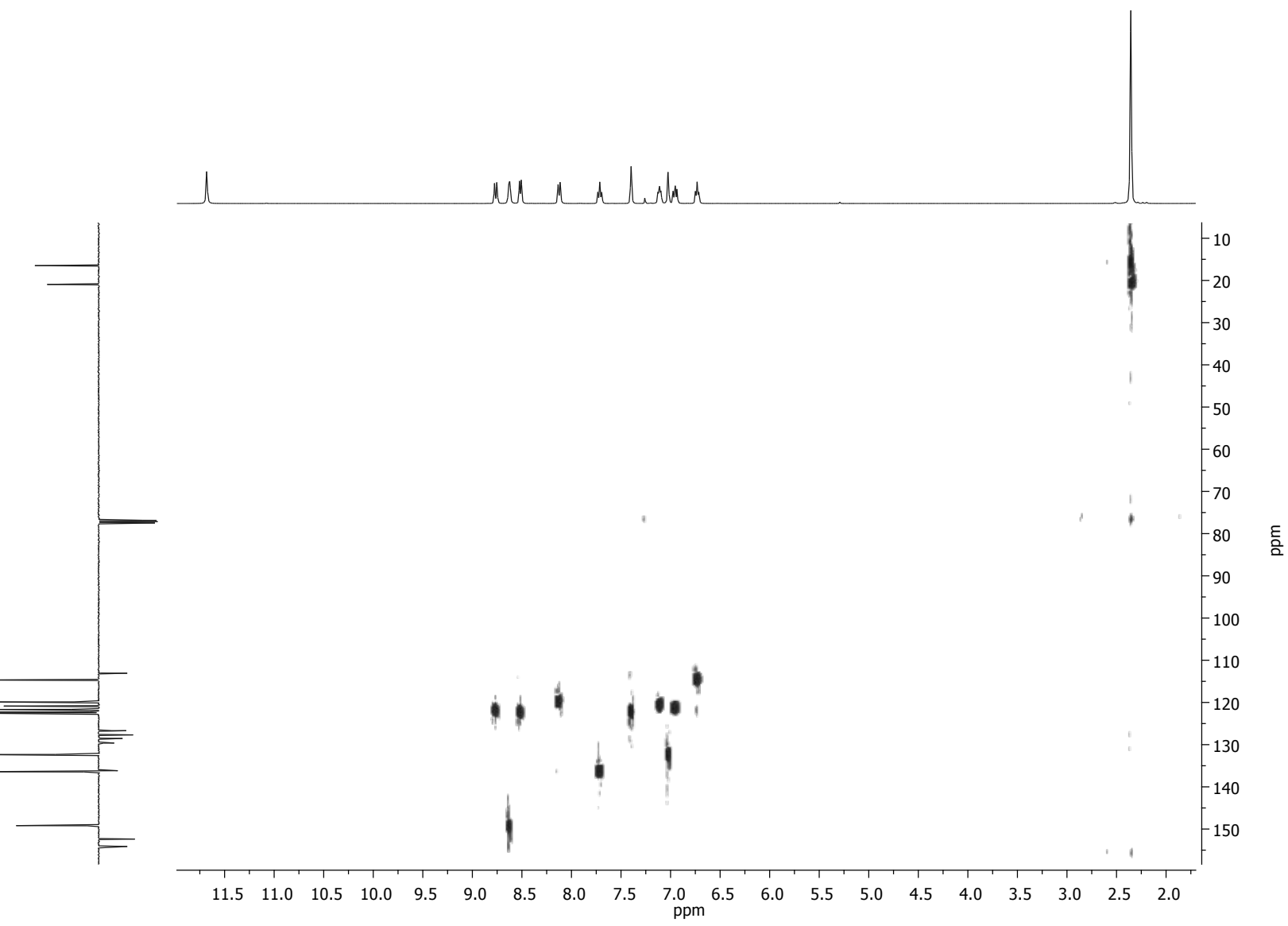

Figure S19. HSQC-NMR spectrum ($CDCl_3$, 298 K) of **10.**

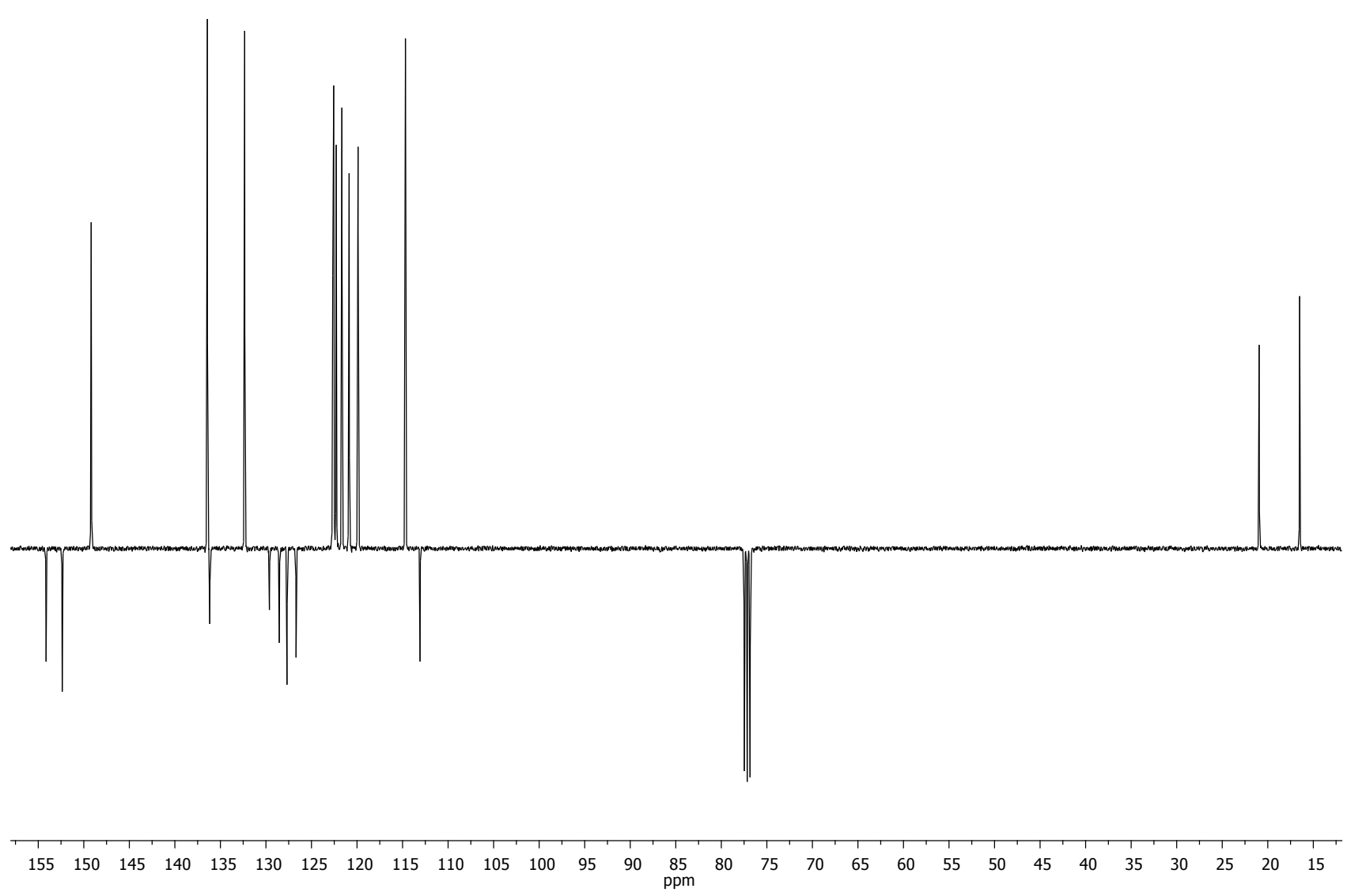

Figure S20. ^{13}C NMR spectrum ($CDCl_3$, 298 K) of **10**.

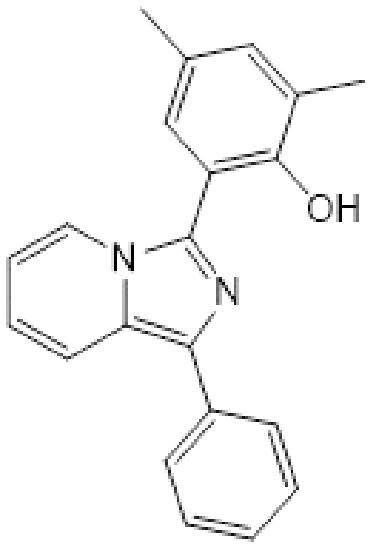

14: ^{1}H NMR (400 MHz, $CDCl_3$, 298 K, *J* [Hz]): δ = 11.88 (s, 1H), 8.52 (d, J = 7.3 Hz, 1H), 7.89 (m, 3H), 7.47 (t, 7.6 Hz, 2H), 7.41 (s, 1H), 7.33 (t, J = 7.3 Hz, 1H), 7.03 (s, 1H), 6.87 – 6.84 (m, 1H), 6.69 (t, J = 6.8 Hz, 1H), 2.36 (s, 6H). ^{13}C NMR (101 MHz, $CDCl_3$, 298 K): δ = 152.50, 136.29, 133.97, 132.30, 129.89, 128.94, 127.64, 127.08, 127.01, 126.88, 126.80, 122.91, 122.39, 120.48, 119.55, 114.09, 113.13, 20.98, 16.55.

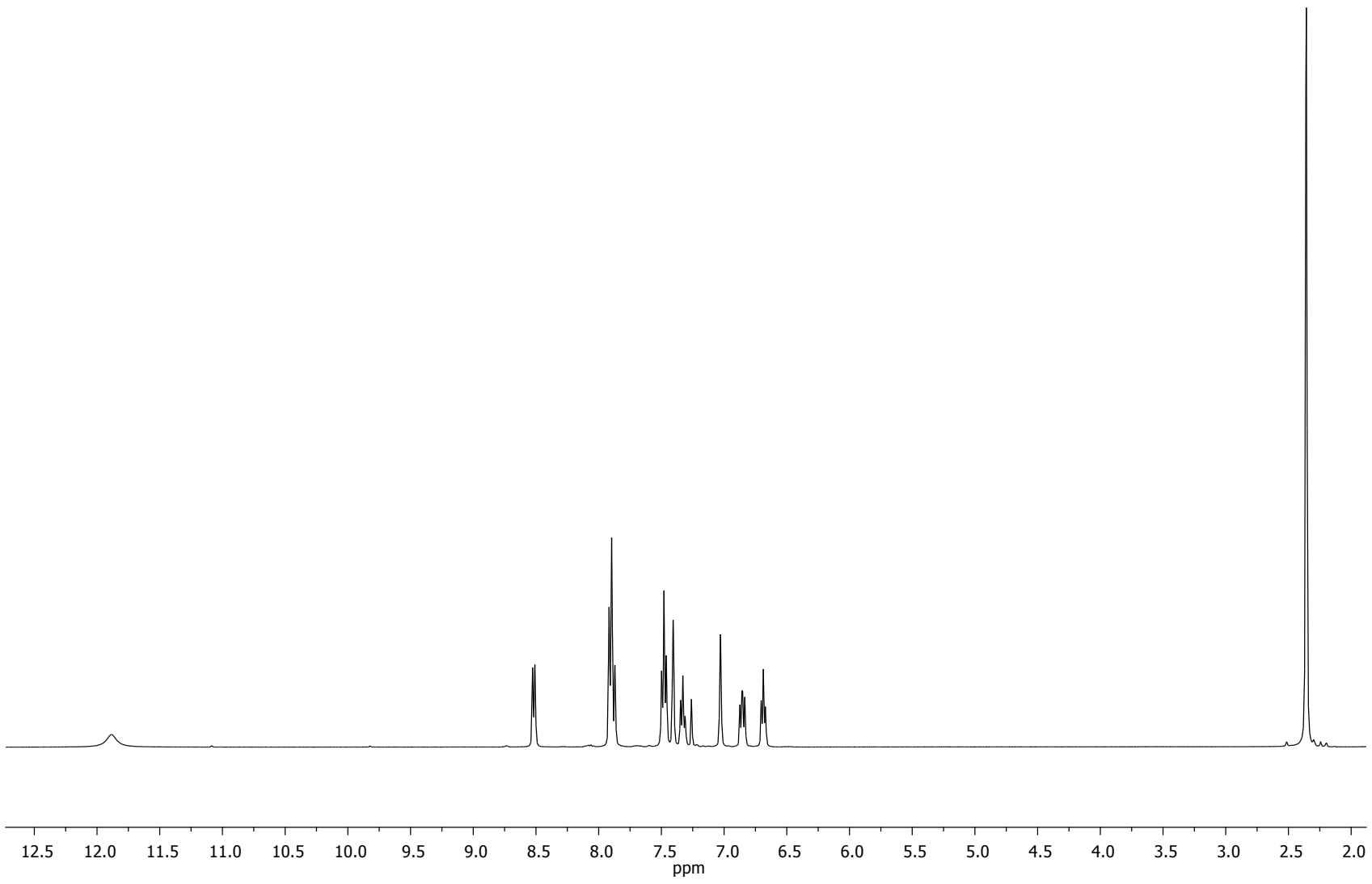

Figure S21. ^{1}H NMR spectrum ($CDCl_3$, 298 K) of **14**.

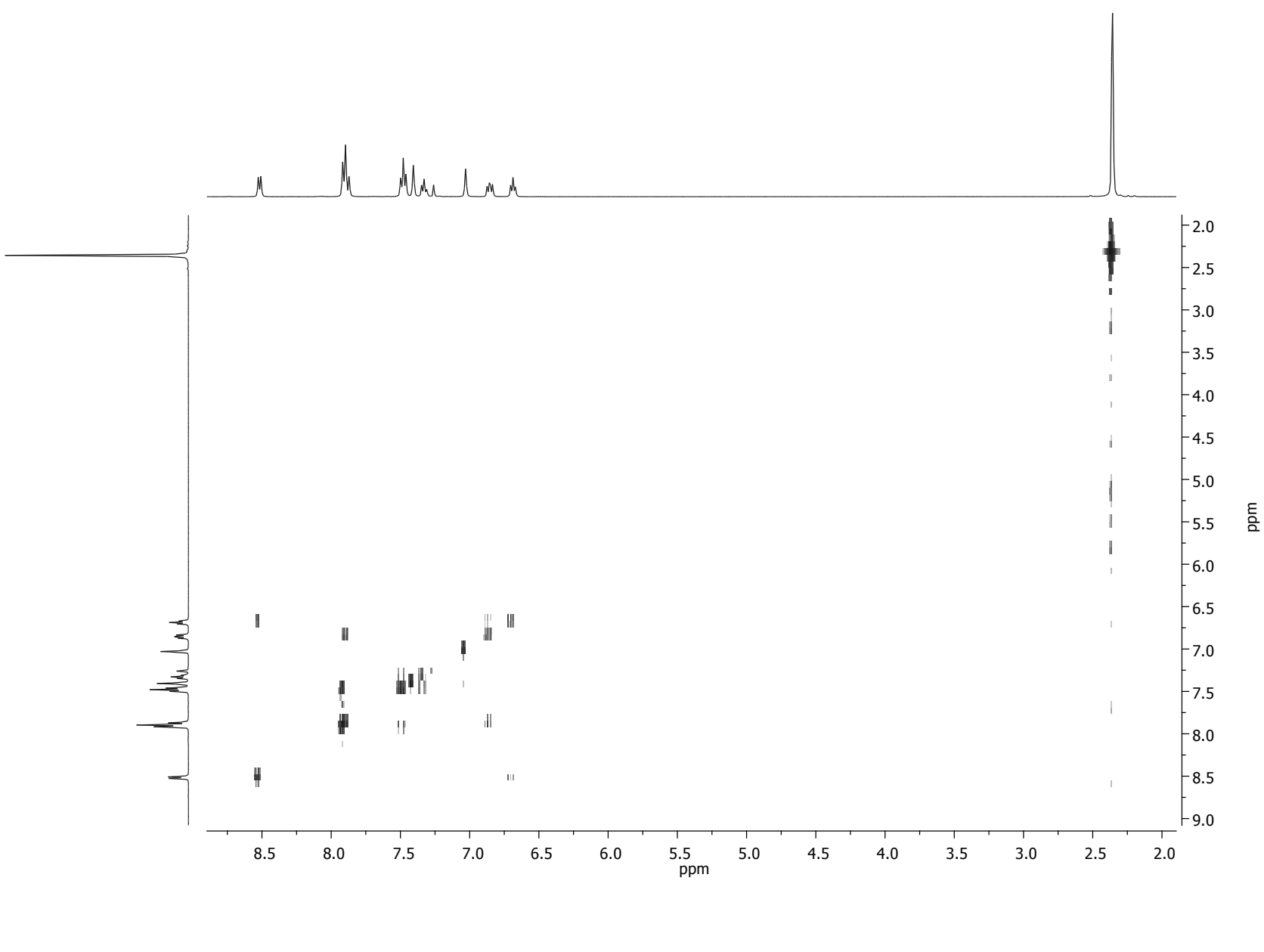

Figure S22. COSY-NMR spectrum ($CDCl_3$, 298 K) of **14.**

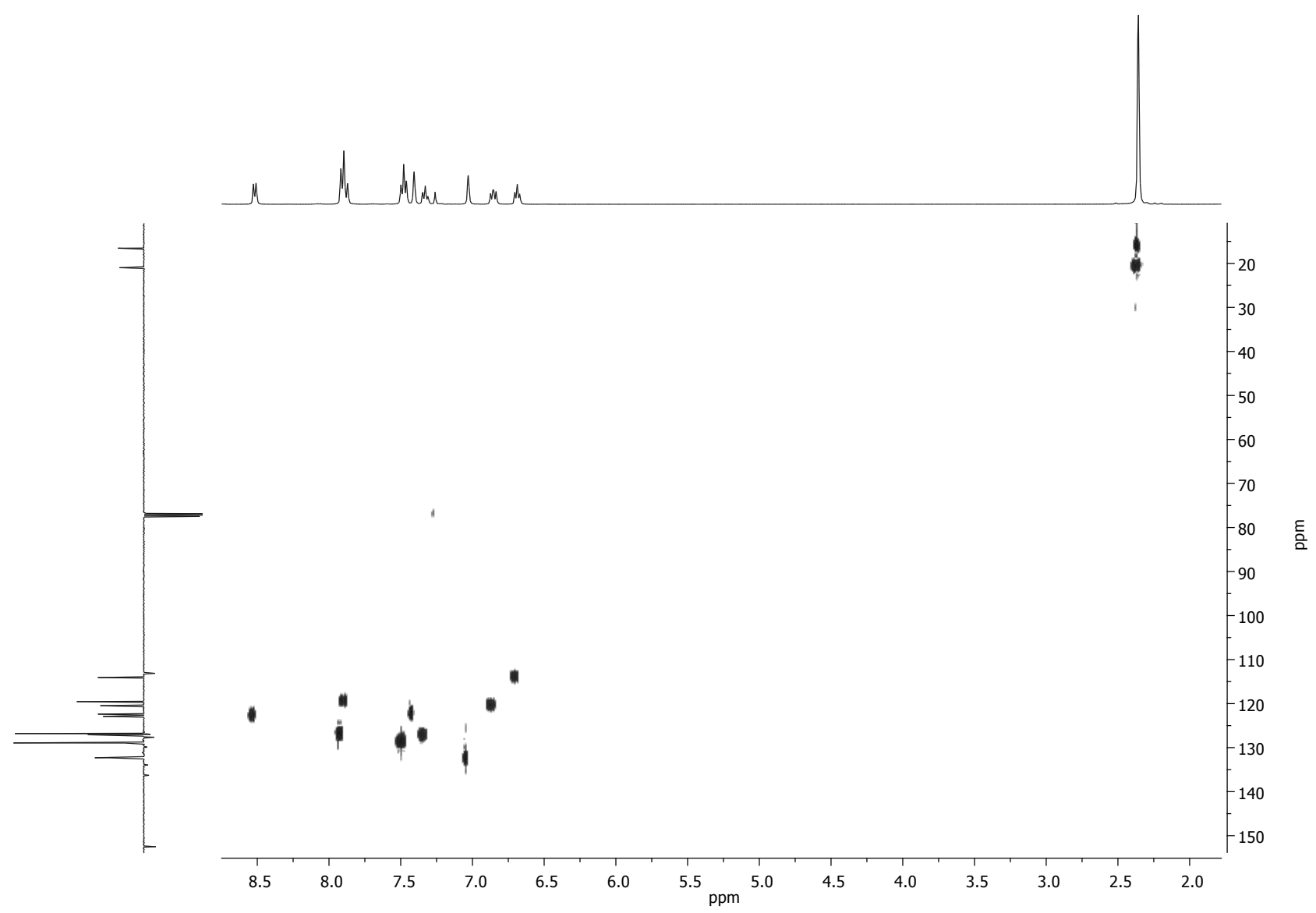

Figure S23. HSQC-NMR spectrum ($CDCl_3$, 298 K) of **14.**

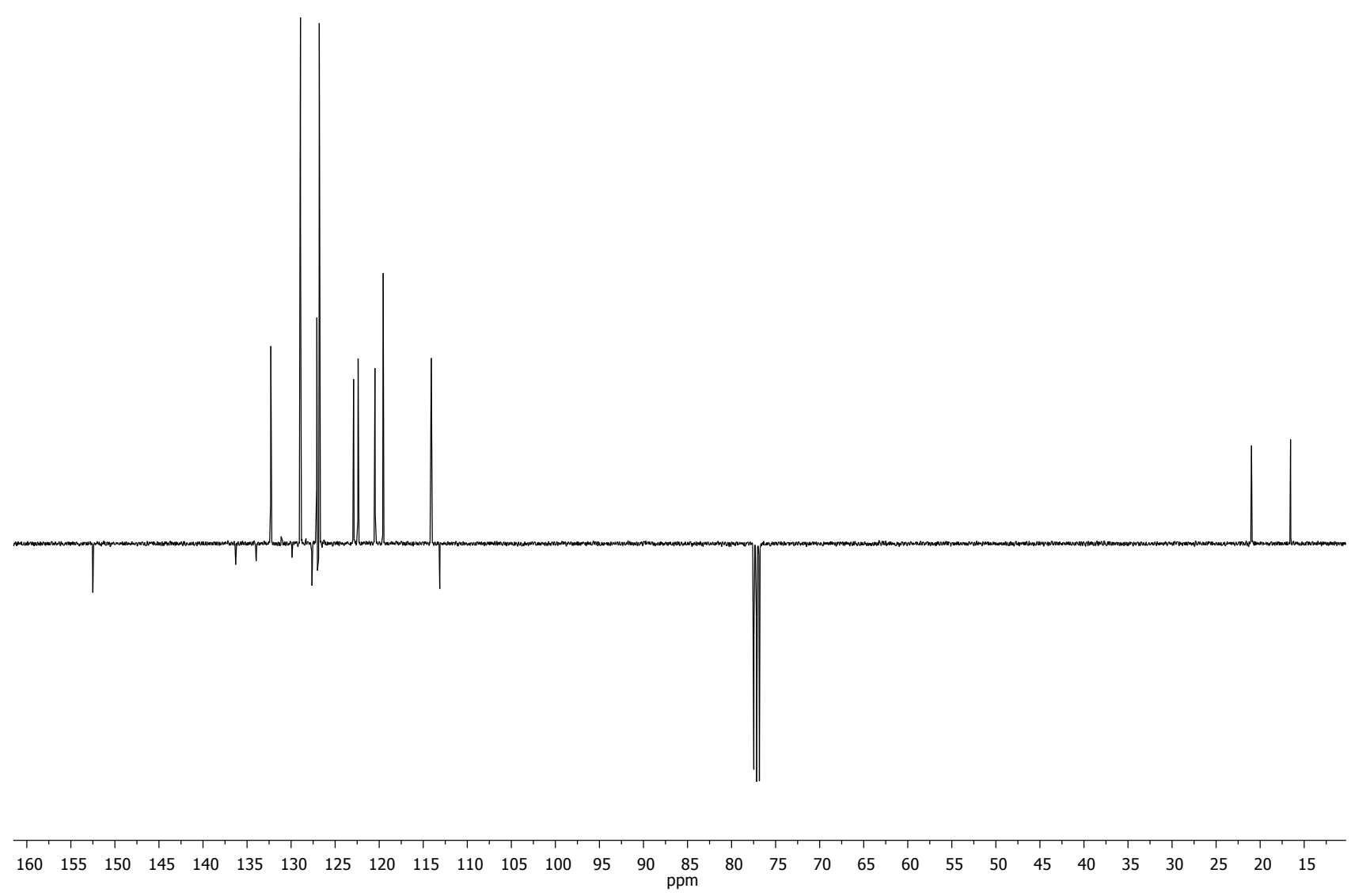

Figure S24. ^{13}C NMR spectrum ($CDCl_3$, 298 K) of **14**.

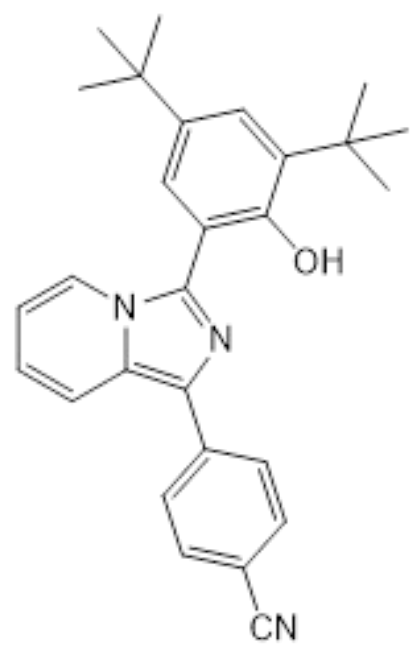

15: ^{1}H NMR (400 MHz, $CDCl_3$, 298 K, *J* [Hz]): δ = 11.21 (s, 1H), 8.49 (d, *J* = 7.3 Hz, 1H), 8.04 (d, *J* = 8.4 Hz, 2H), 7.90 (d, *J* = 9.3 Hz, 1H), 7.75 (d, *J* = 8.4 Hz, 2H), 7.57 (d, *J* = 2.2 Hz, 1H), 7.44 (d, *J* = 2.2 Hz, 1H), 7.02 – 6.98 (m, 1H), 6.76 (t, *J* = 6.7 Hz, 1H), 1.51 (s, 9H), 1.38 (s, 9H). ^{13}C NMR (101 MHz, $CDCl_3$, 298 K): δ = 152.77, 141.05, 132.81, 128.43, 126.71, 125.67, 123.17, 122.41, 120.04, 119.29, 119.01, 114.49, 112.95, 35.56, 34.65, 31.77, 29.71.

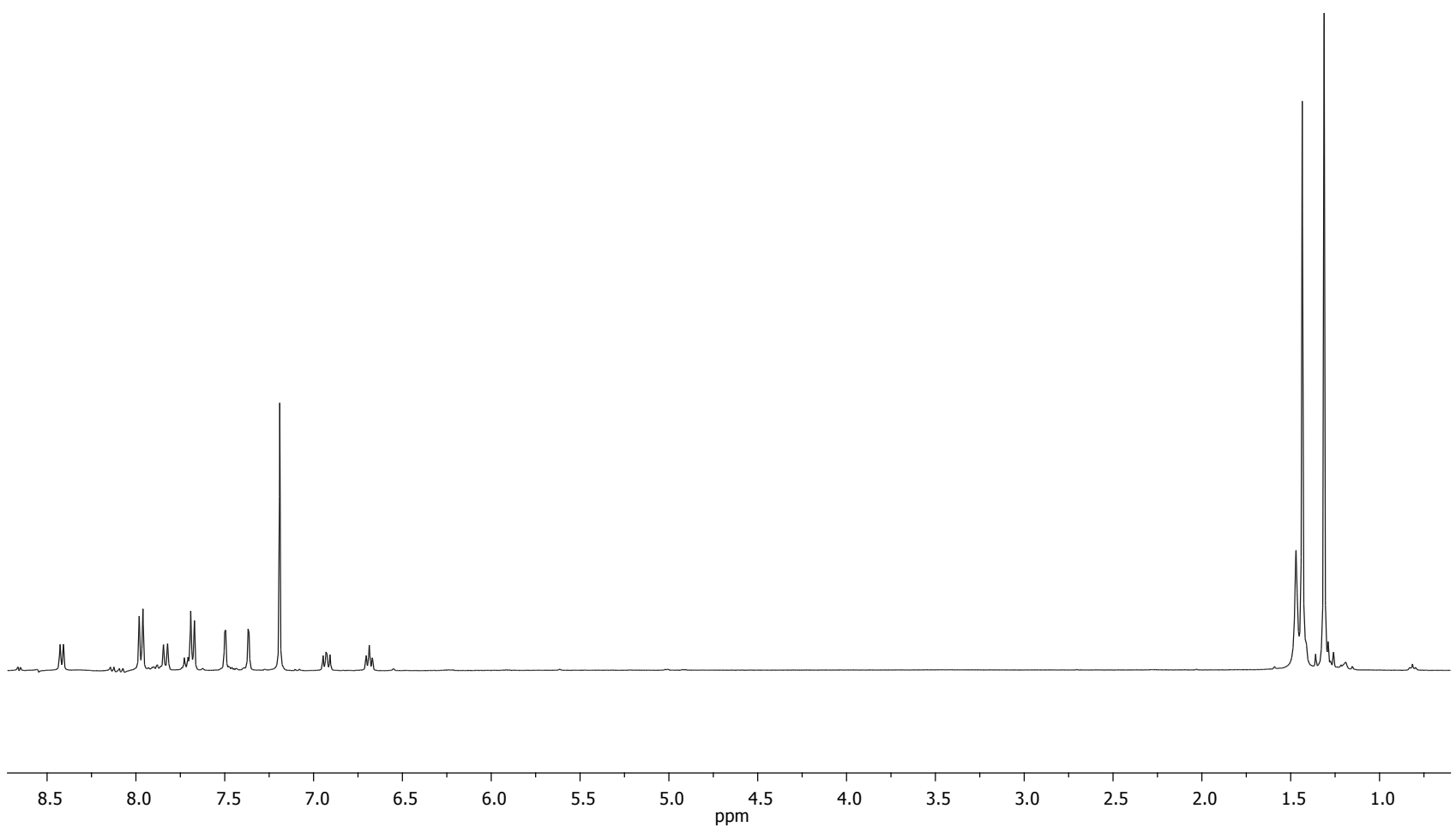

Figure S25. ^{1}H NMR spectrum ($CDCl_3$, 298 K) of **15**.

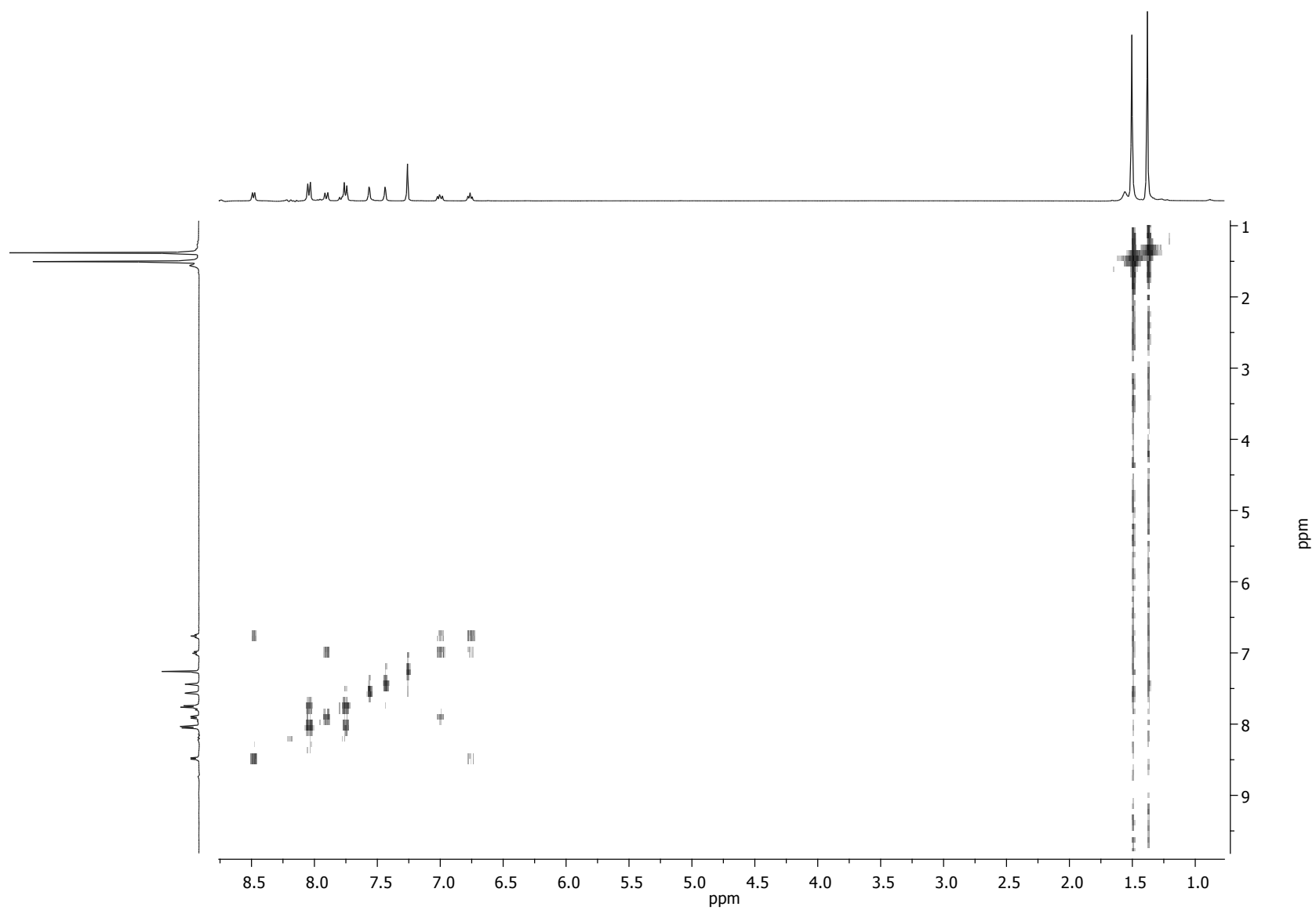

Figure S26. COSY-NMR spectrum ($CDCl_3$, 298 K) of **15.**

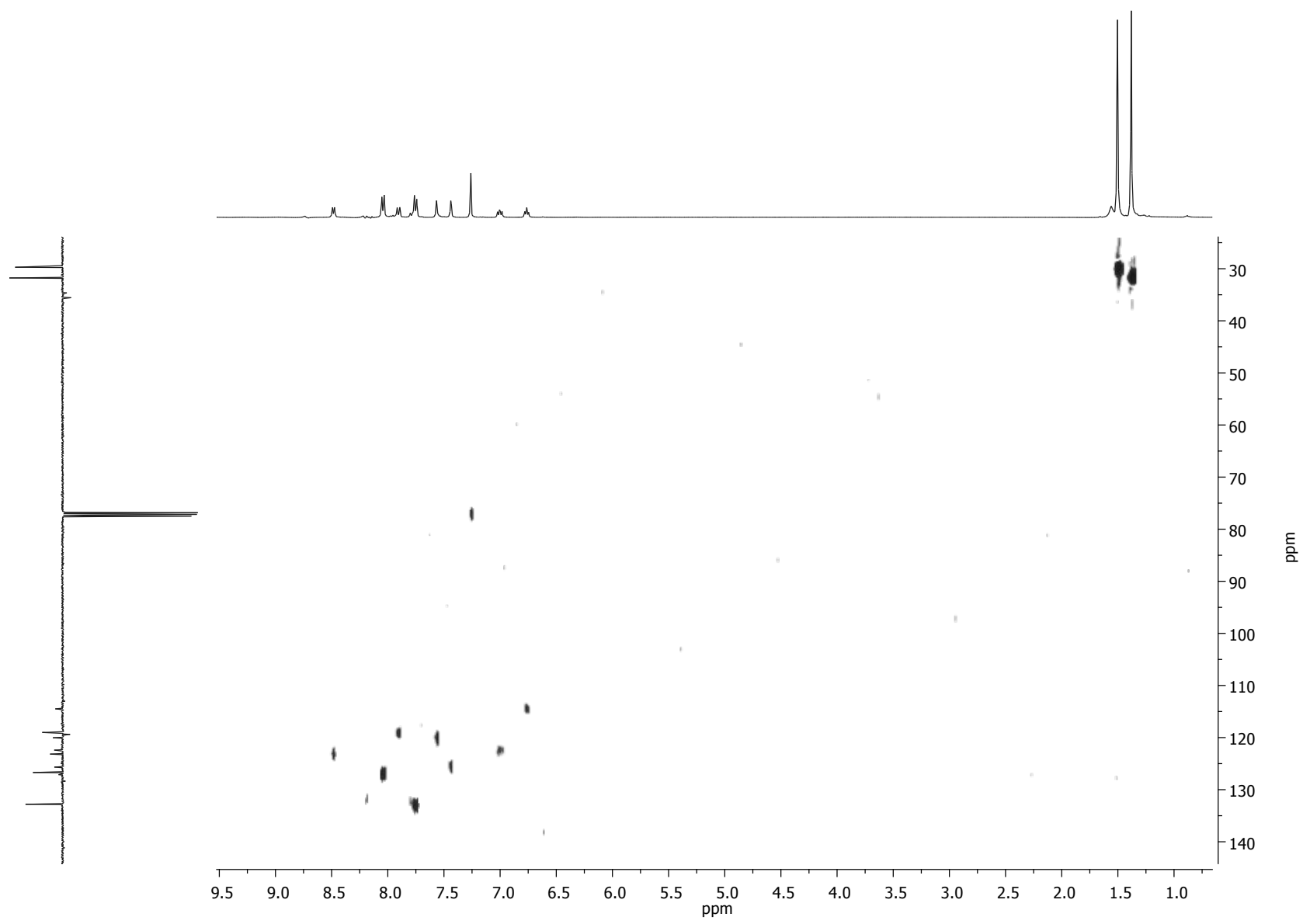

Figure S27. HSQC-NMR spectrum ($CDCl_3$, 298 K) of **15.**

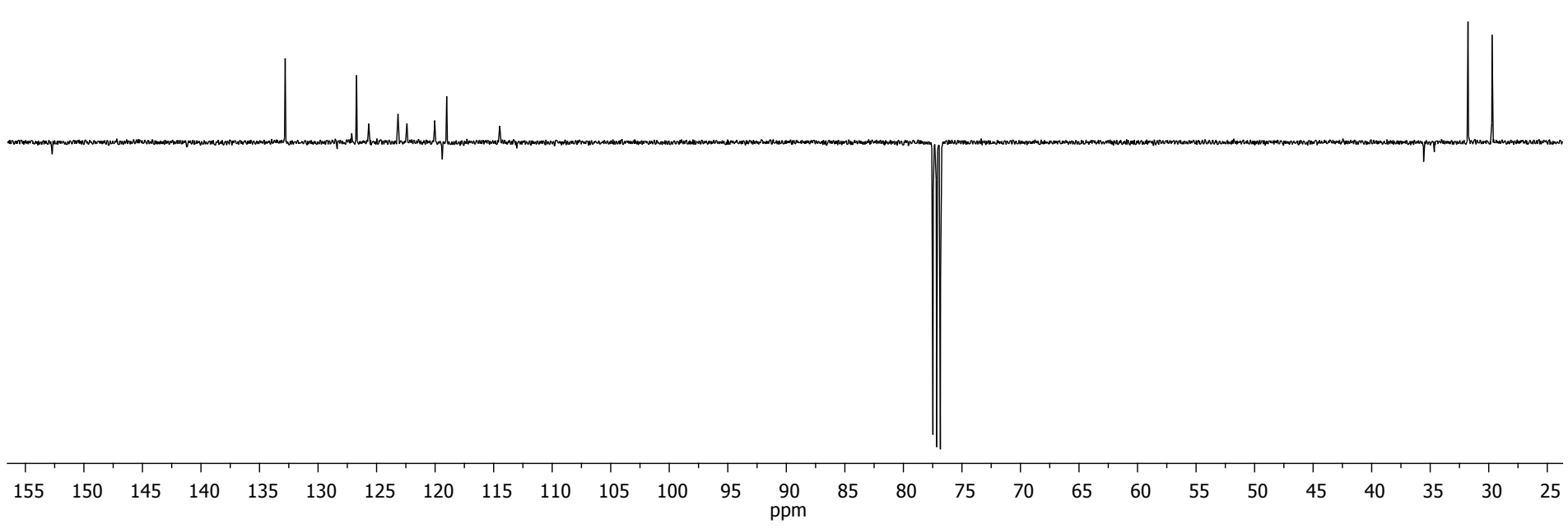

Figure S28. ^{13}C NMR spectrum ($CDCl_3$, 298 K) of **15**.

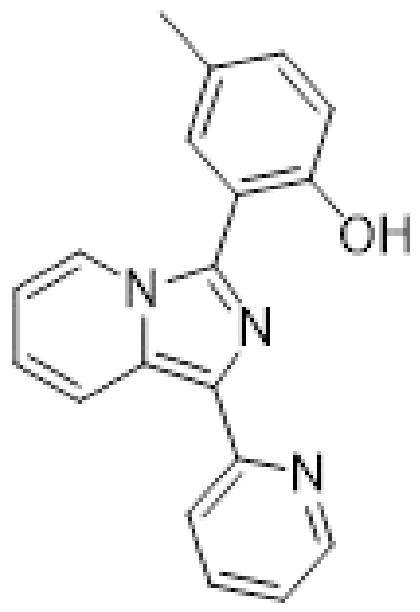

20: ^{1}H NMR (400 MHz, $CDCl_3$, 298 K, *J* [Hz]): δ = 11.47 (s, 1H), 8.79 (d, *J* = 9.2 Hz, 1H), 8.64 (d, *J* = 4.1 Hz, 1H), 8.54 (d, *J* = 7.2 Hz, 1H), 8.12 (d, *J* = 8.0 Hz, 1H), 7.74 (t, *J* = 7.7 Hz, 1H), 7.57 (s, 1H), 7.18 – 7.05 (m, 3H), 7.01 – 6.97 (m, 1H), 6.77 (t, *J* = 6.7 Hz, 1H), 2.39 (s, 3H). ^{13}C NMR (101 MHz, $CDCl_3$, 298 K): δ = 154.26, 154.10, 149.23, 136.53, 135.77, 130.97, 129.70, 128.77, 128.37, 124.94, 122.53, 122.39, 121.77, 120.97, 119.94, 117.74, 114.88, 113.86, 21.00.

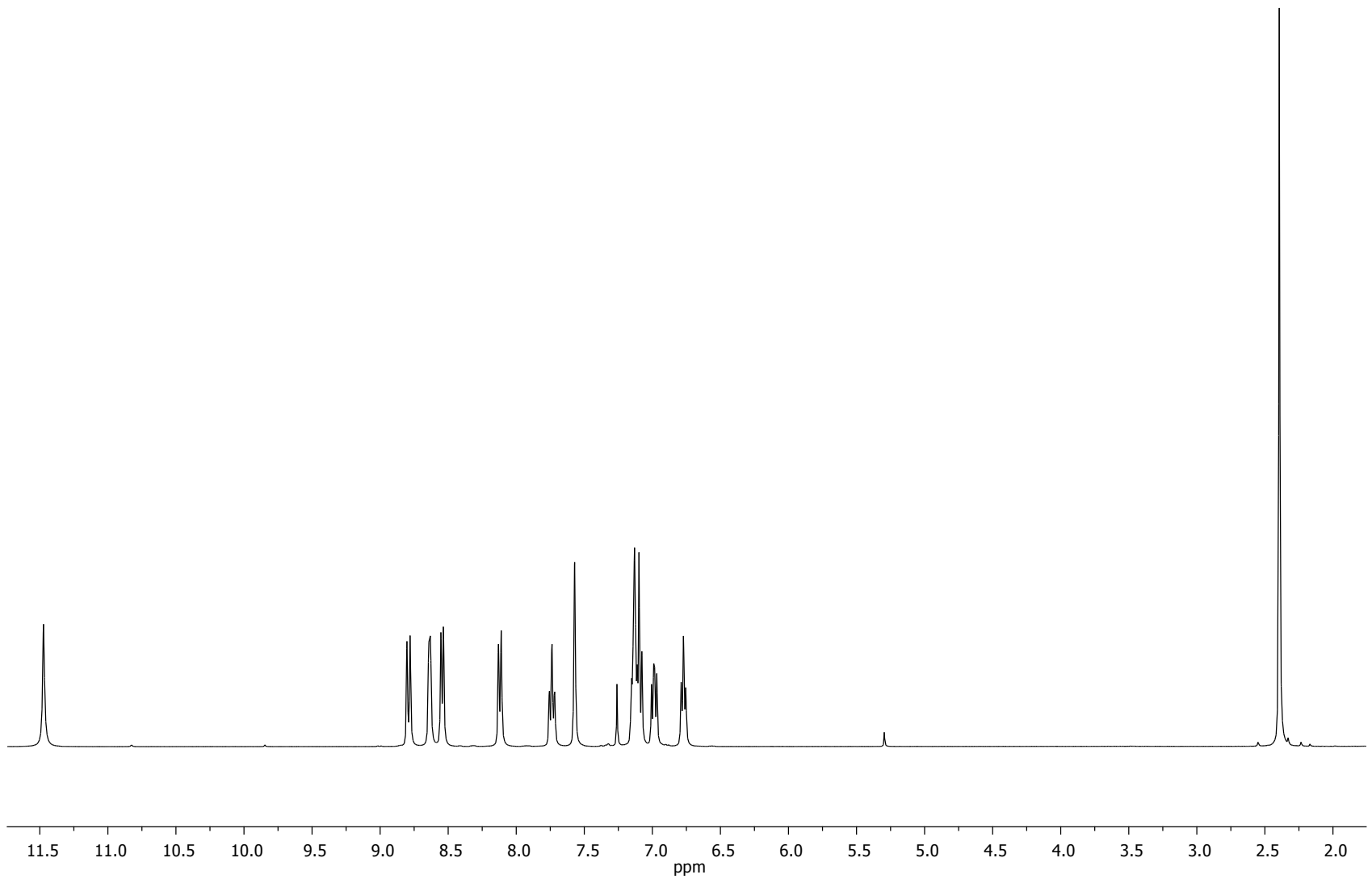

Figure S29. ^{1}H NMR spectrum ($CDCl_3$, 298 K) of **20**.

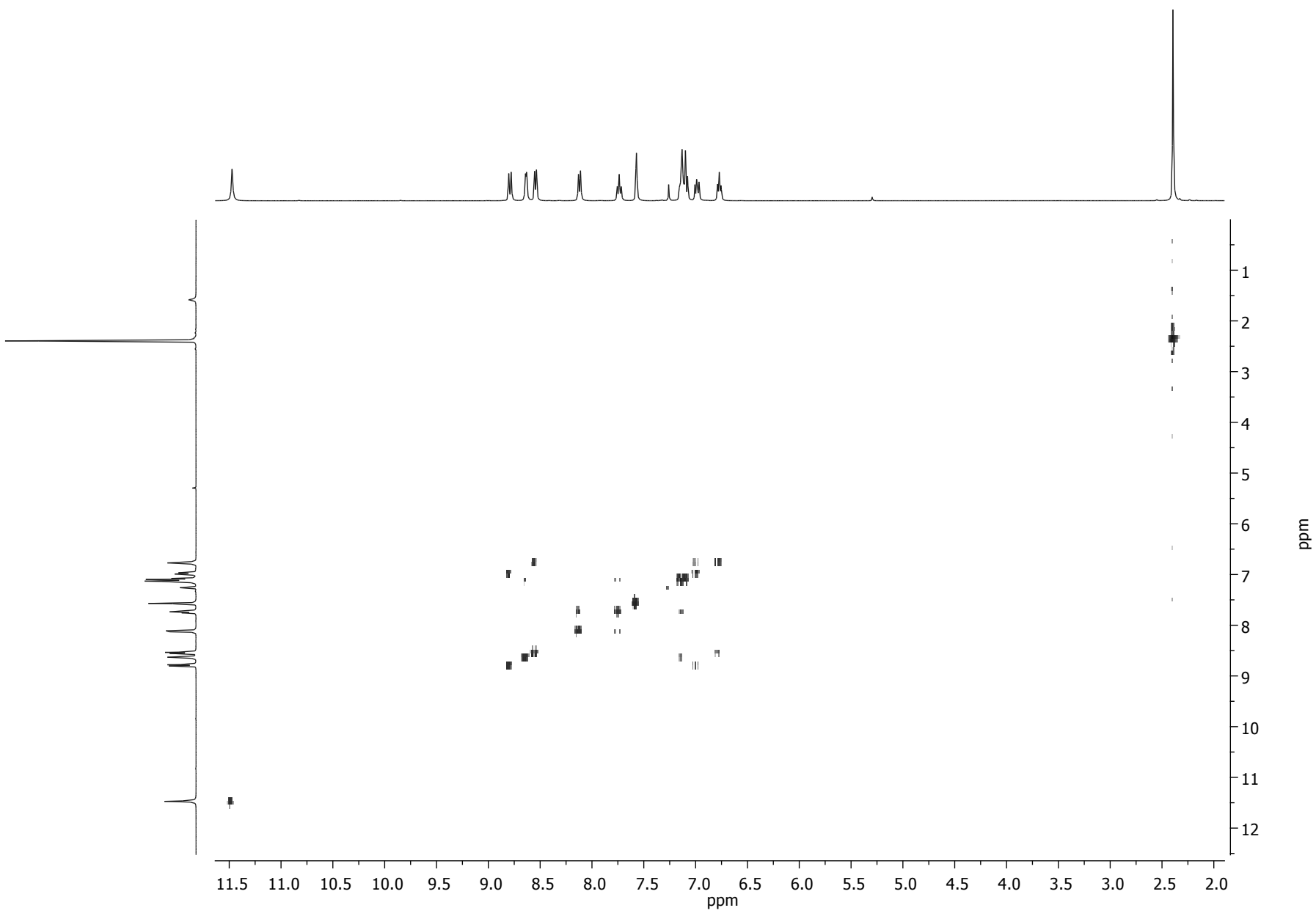

Figure S30. COSY-NMR spectrum ($CDCl_3$, 298 K) of **20.**

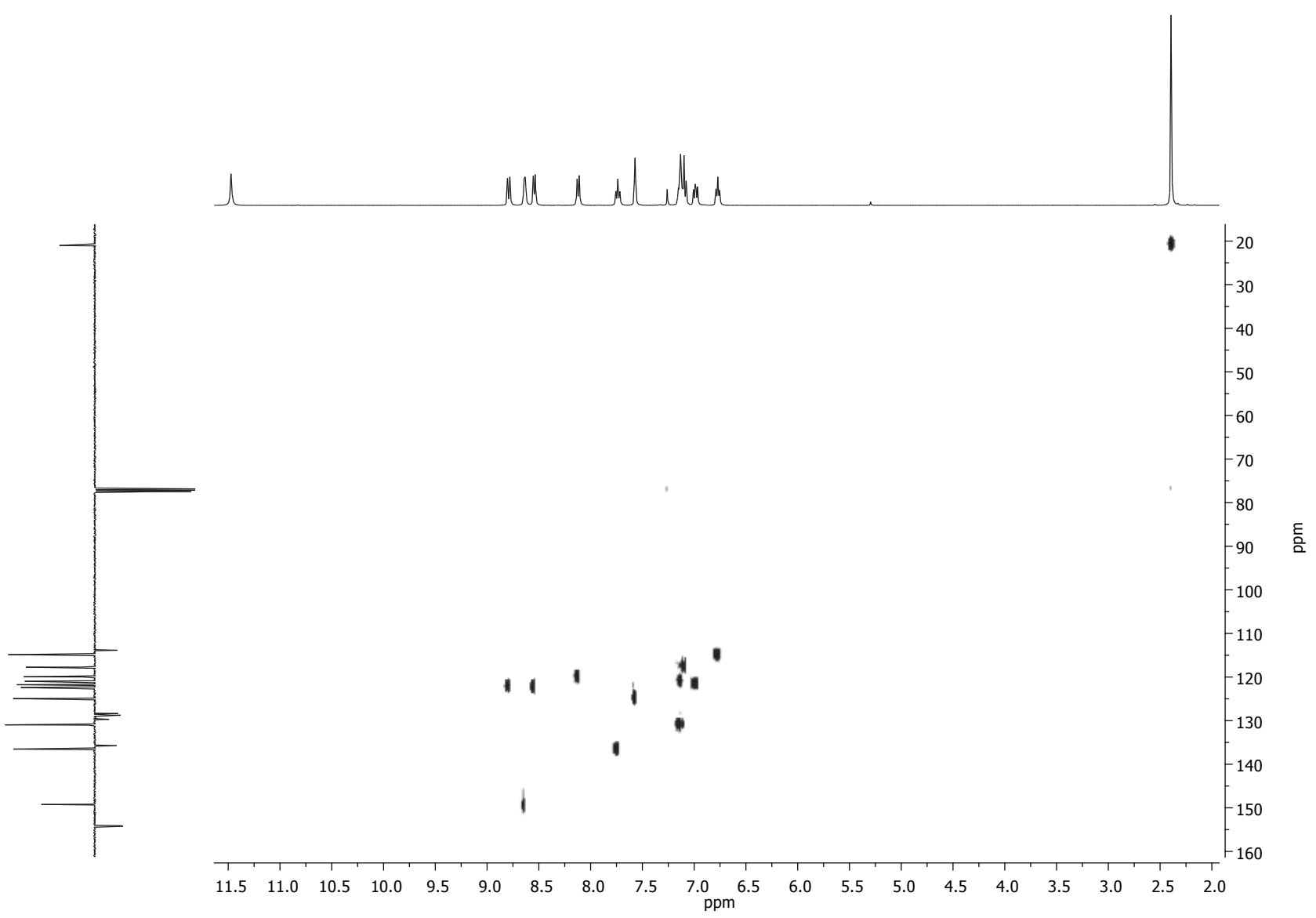

Figure S31. HSQC-NMR spectrum ($CDCl_3$, 298 K) of **20.**

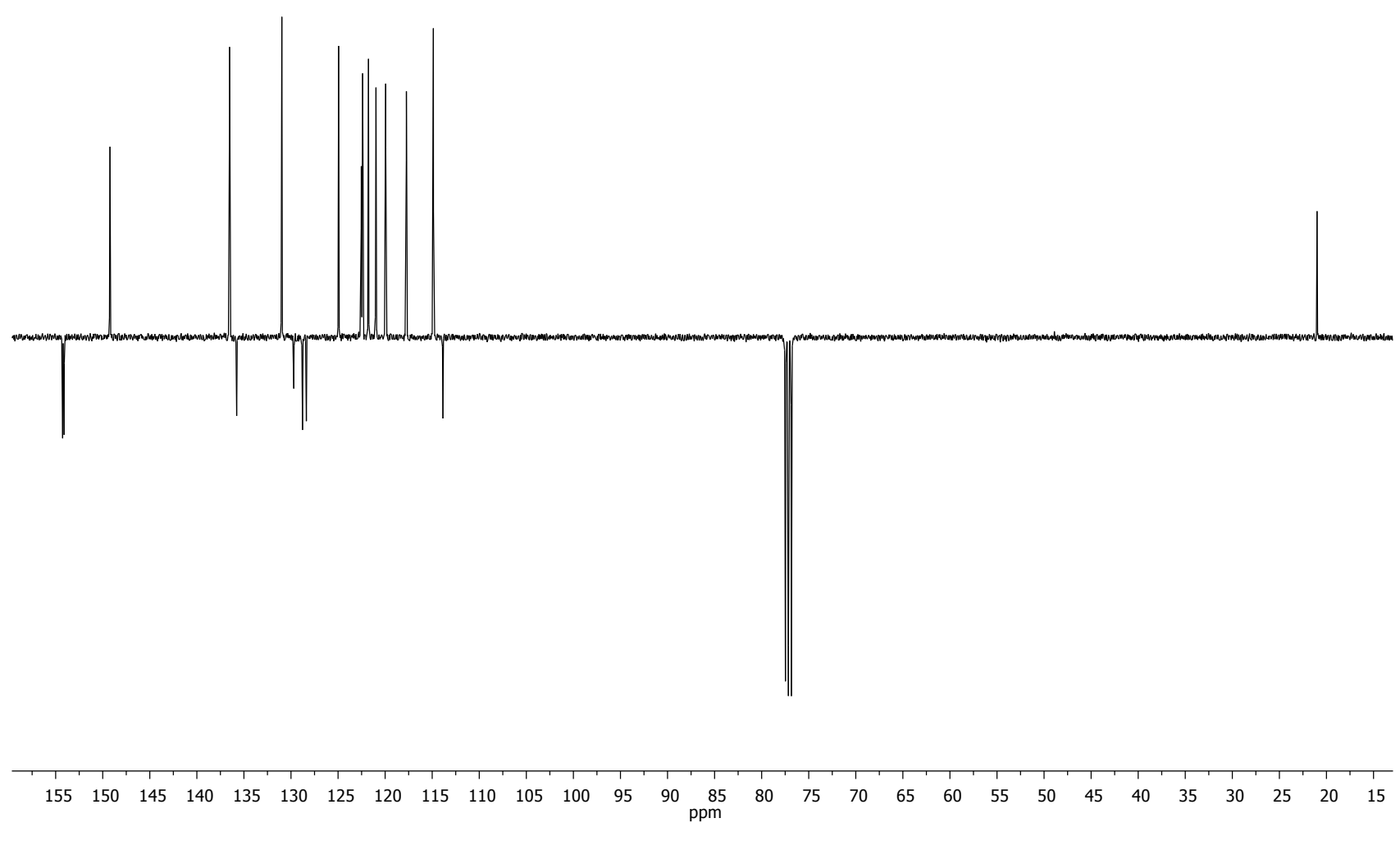

Figure S32. ^{13}C NMR spectrum ($CDCl_3$, 298 K) of **20**.

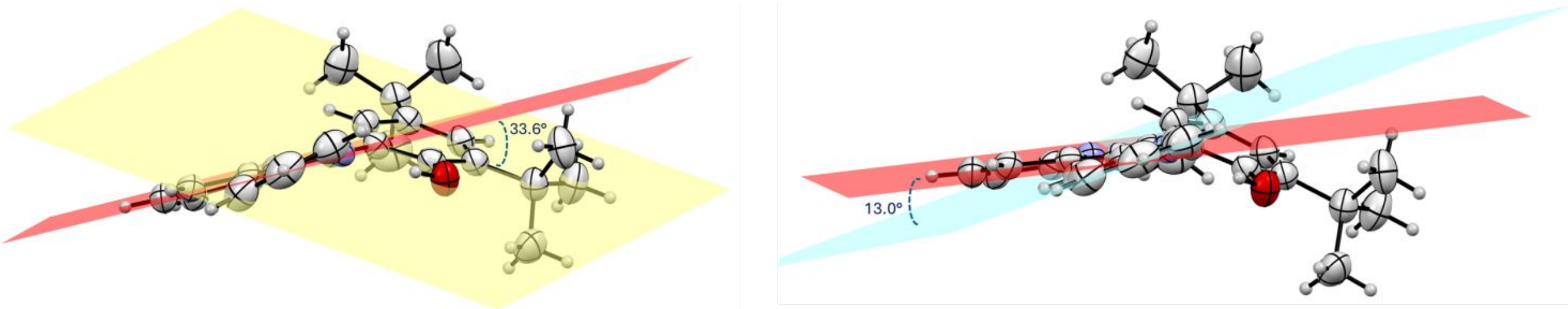

Figure S33. Crystal structure of compound **7** in which are highlighted the angles between the imidazopyridine moiety and the phenol ring (left) and between the imidazopyridine moiety and the phenyl substituent (right).

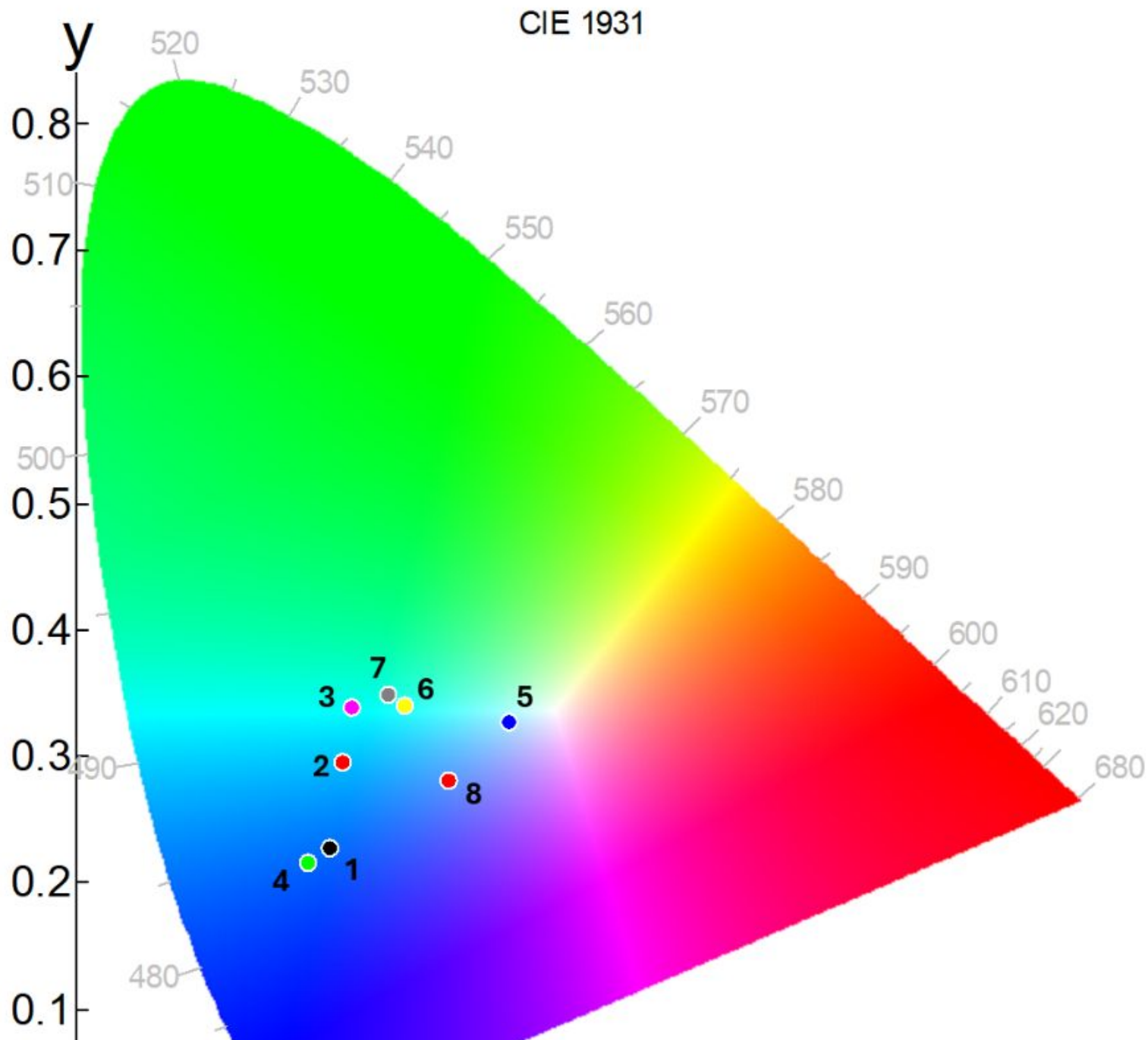

Figure S34. CIE 1931 chromaticity plot for compounds **1**-**8** recorded in solution (CH_2Cl_2, $5 \cdot 10^{-5}$ M).

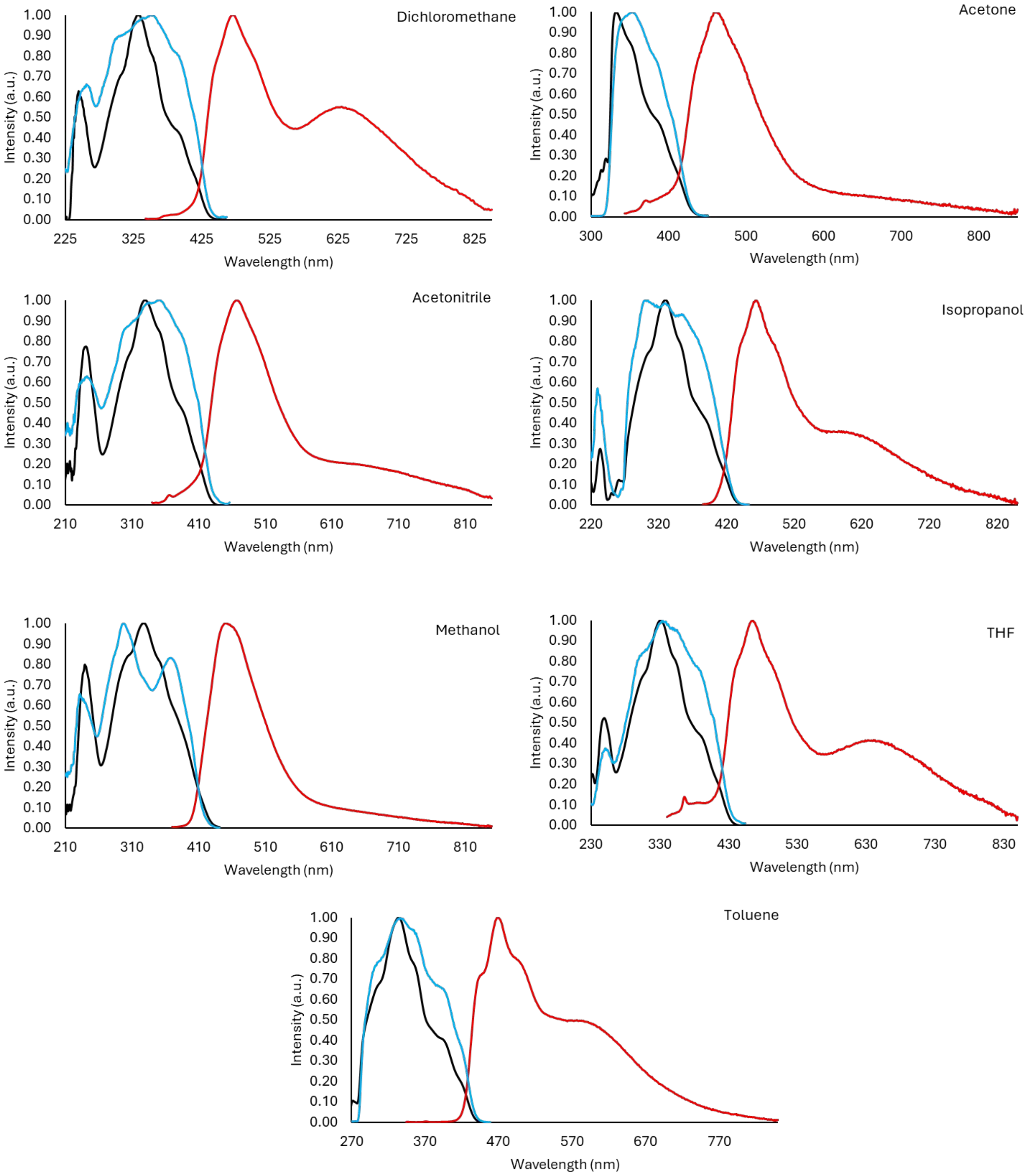

Figure S35. Normalized absorption (black), emission (red) and excitation (light blue) spectra of compounds **8** recorded in solution ($5{\cdot}10^{-5}$ M) in different solvents.

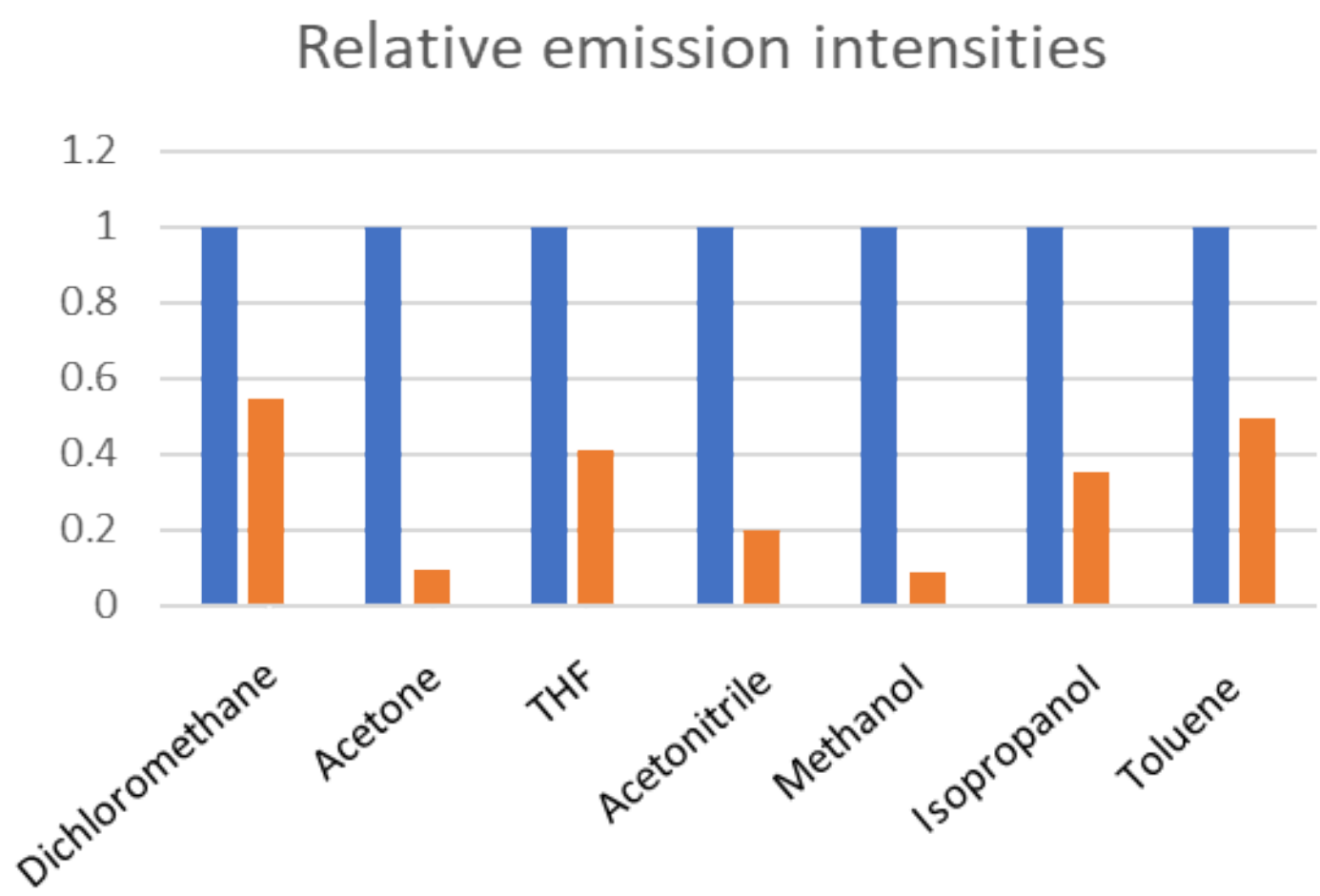

Figure S36. Relative emission intensities of the enol (blue, normalized) and keto (orange) forms of compound **8** measured in different solvents.

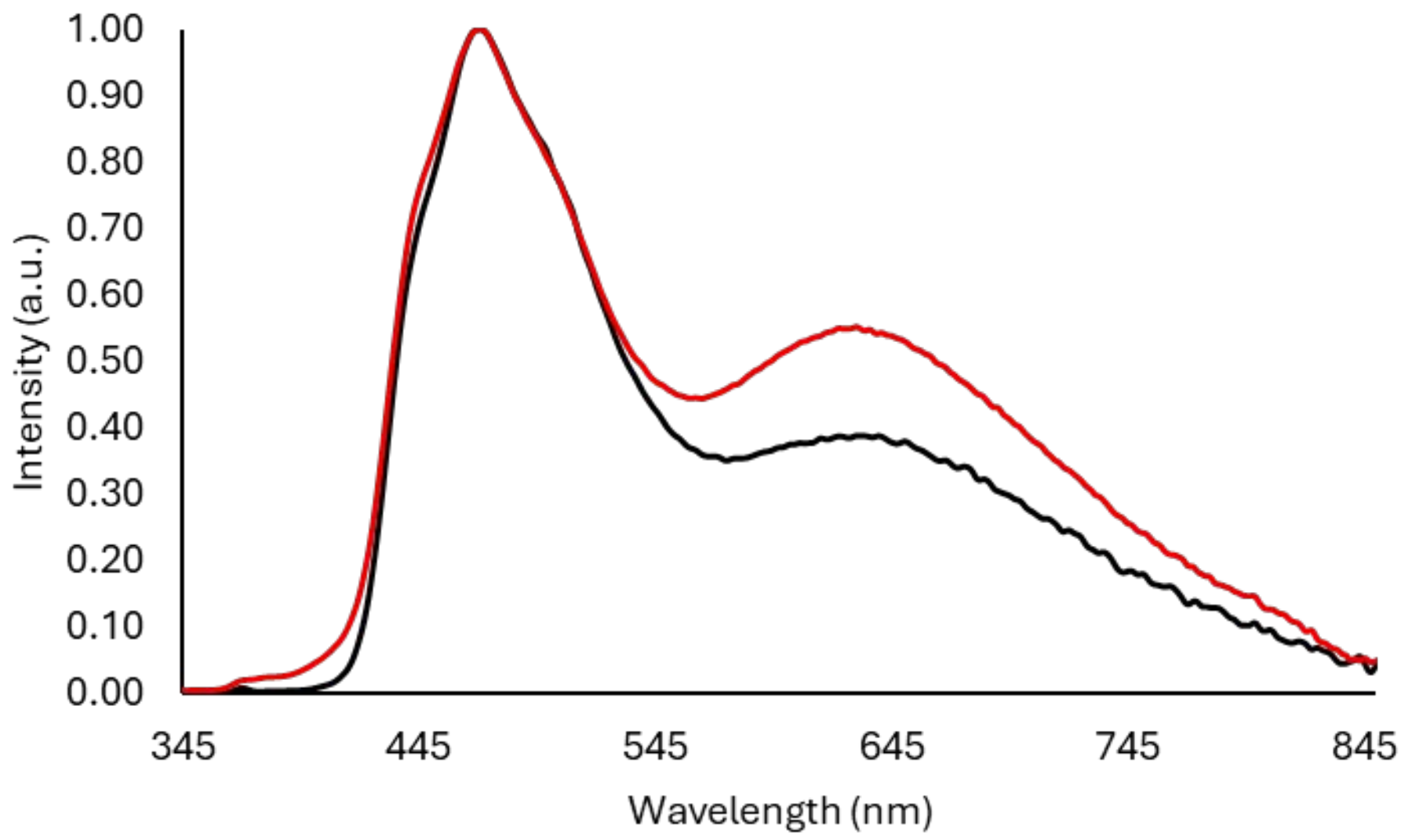

Figure S37. Normalized emission spectra of compound **8** measured in dichloromethane solution ($5x10^{-5}$ M) before (red) and after (black) deuteration with D_2O.

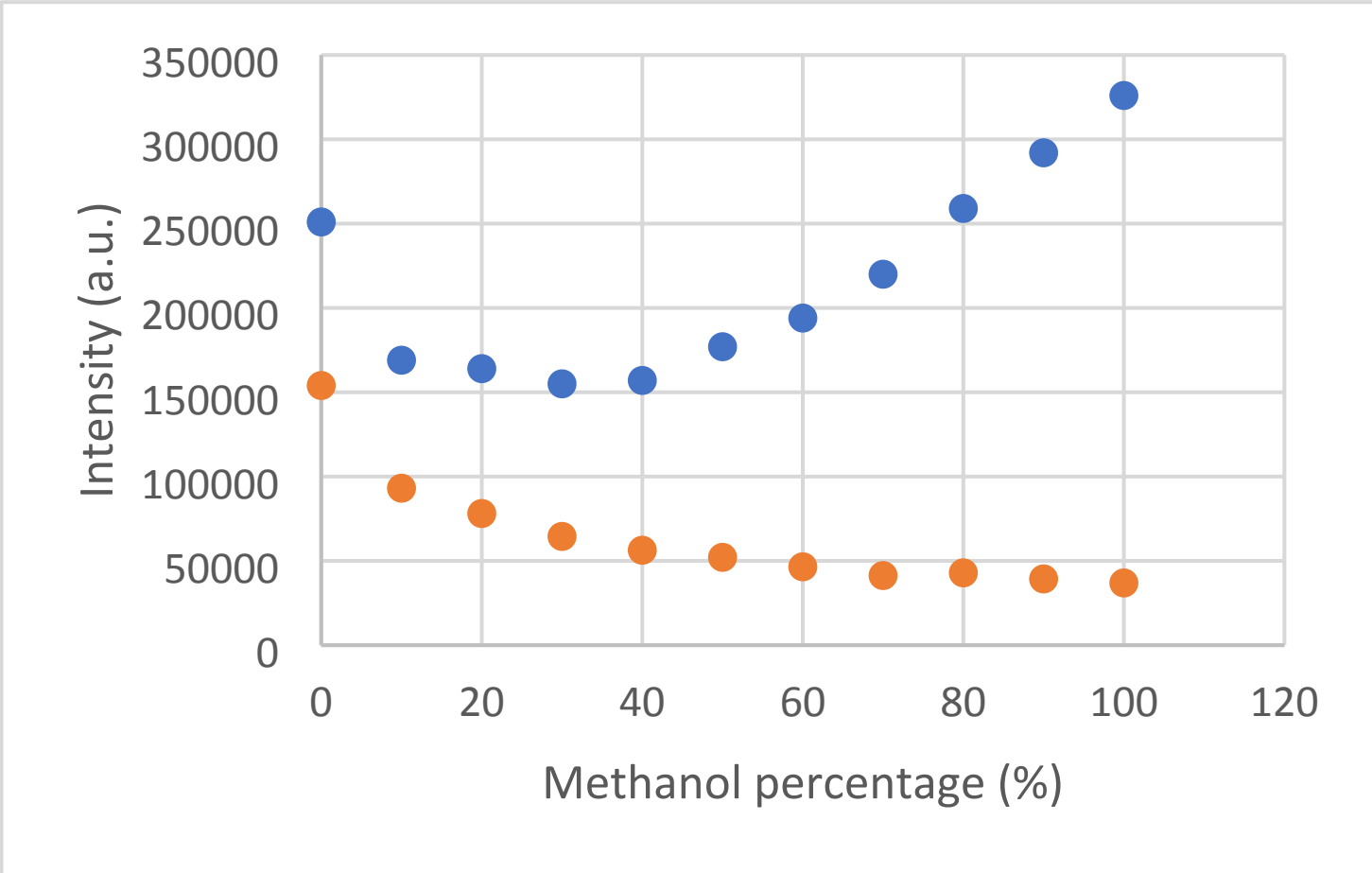

Figure S38. Relative emission intensities of the enol (blue, normalized) and keto (orange) forms of compound **8** measured in dichloromethane/methanol solution (5x10^{-5} M) with different solvent ratios.

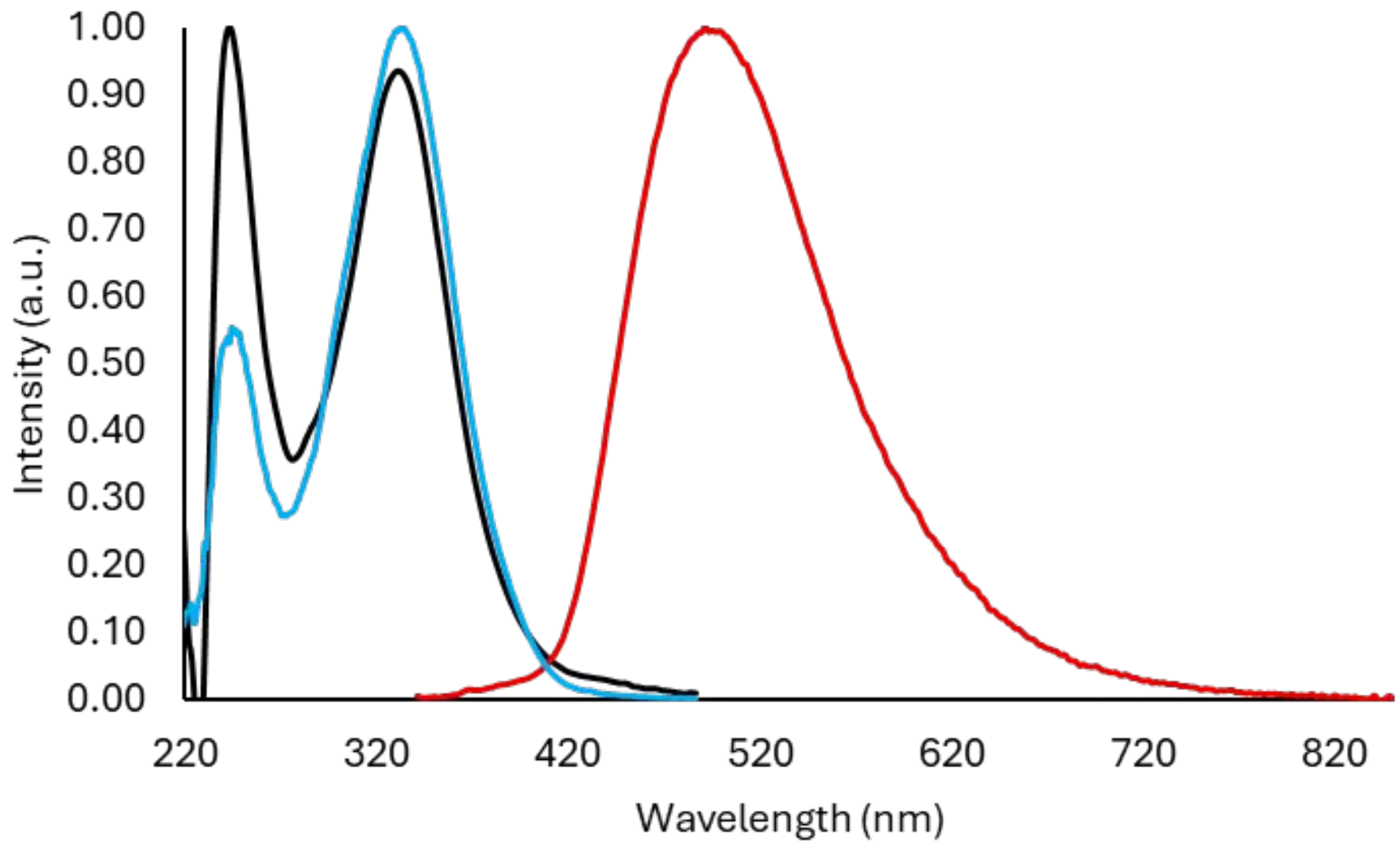

Figure S39. Normalized absorption (black), emission (red) and excitation (light blue) spectra of compound **9** measured in dichloromethane solution (5x10^{-5}).

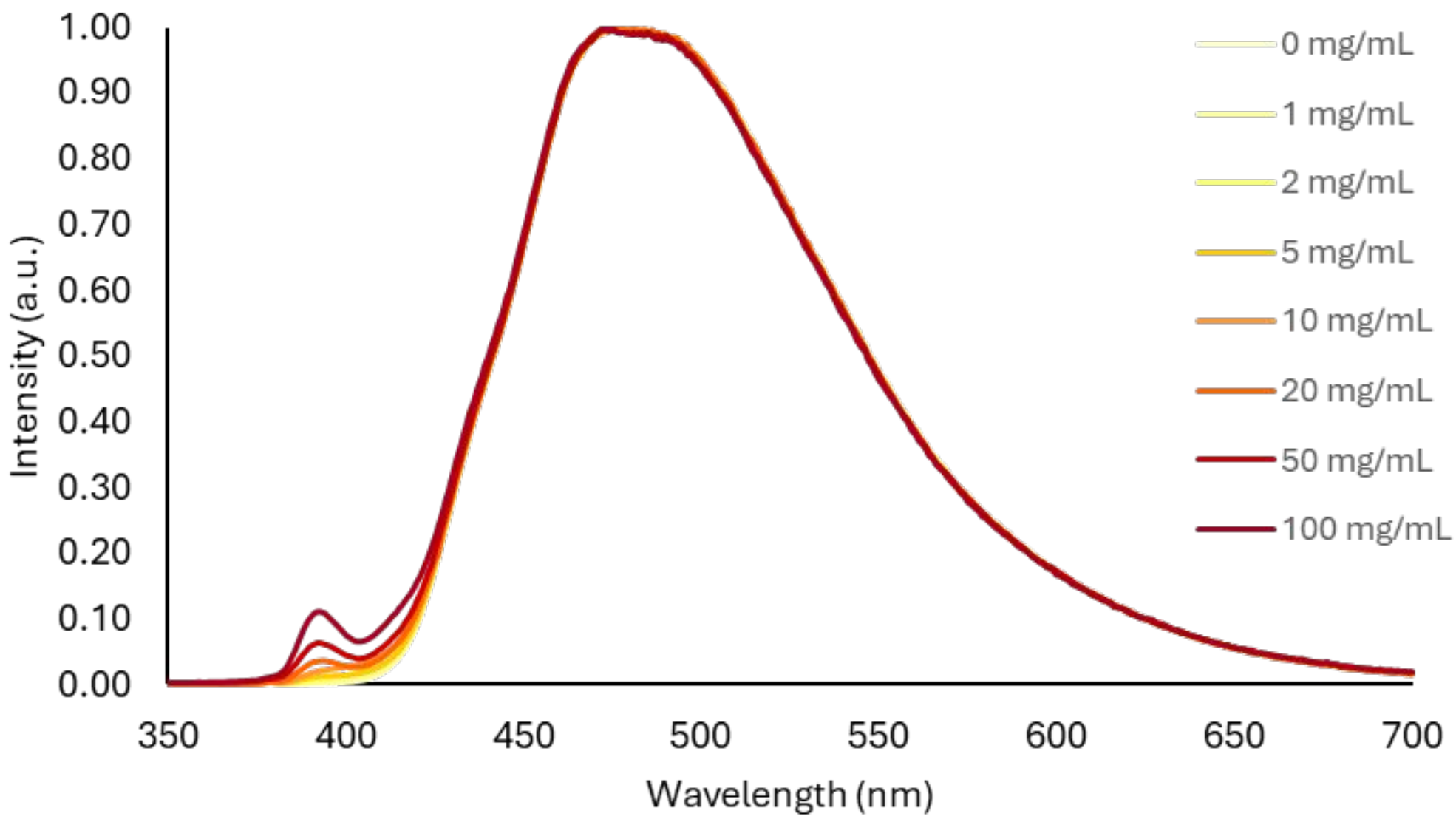

Figure S40. Normalized emission spectra of compound **2** measure in dichloromethane solution ($5x10^{-5}$ M), increasing the viscosity of the solution with eicosane.

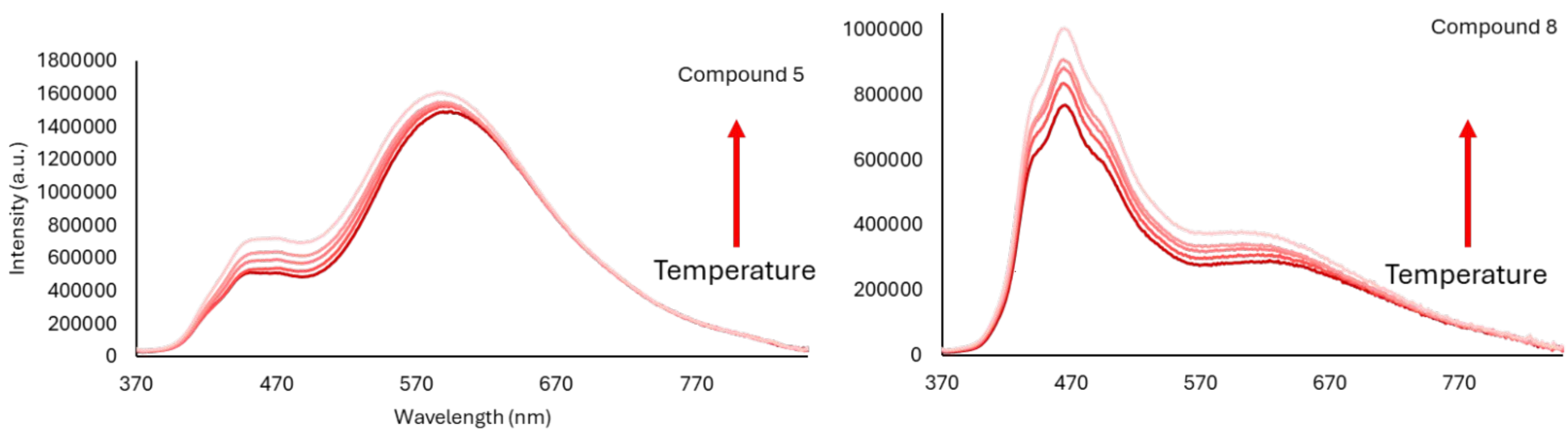

Figure S41. Emission spectra of compounds **5** and **8** measured in 2-methyl tetrahydrofuran ($5x10^{-5}$ M) at different temperatures.

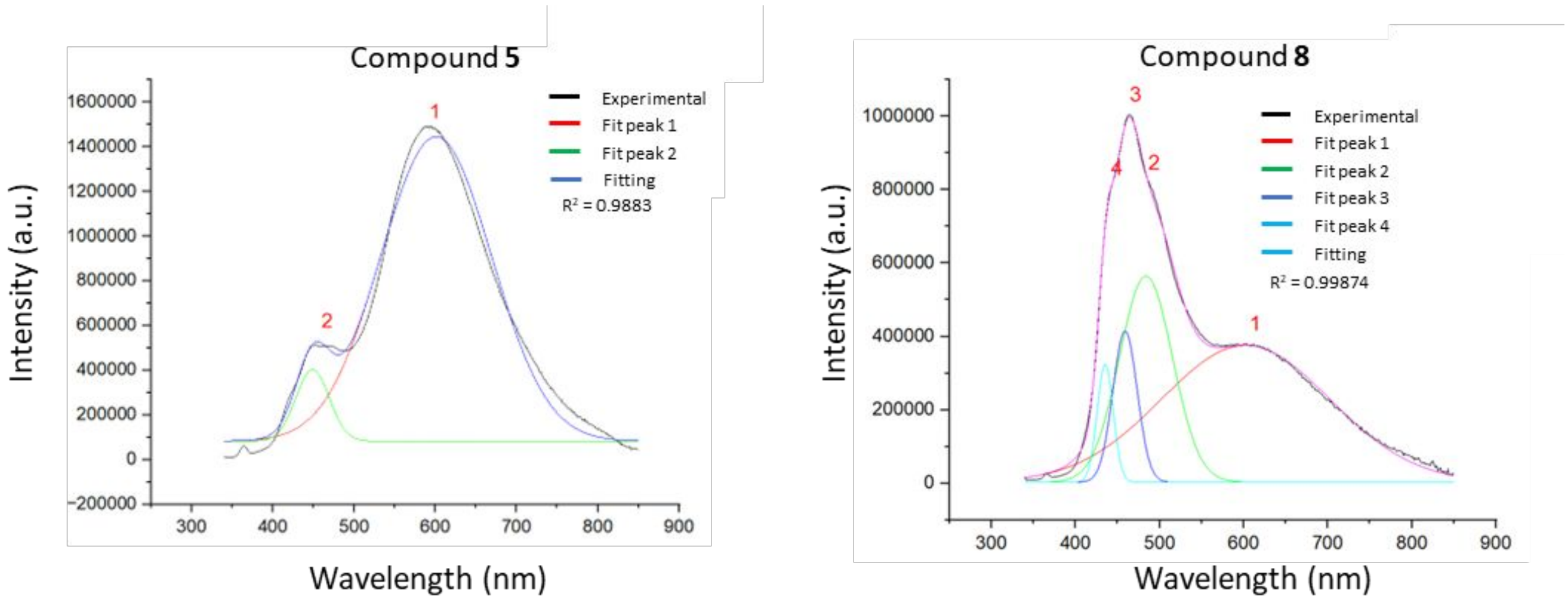

Figure S42. Deconvolution of the emission spectra of compounds **5** and **8** measured in 2-methyl tetrahydrofuran solution (5x10^{-5} M).

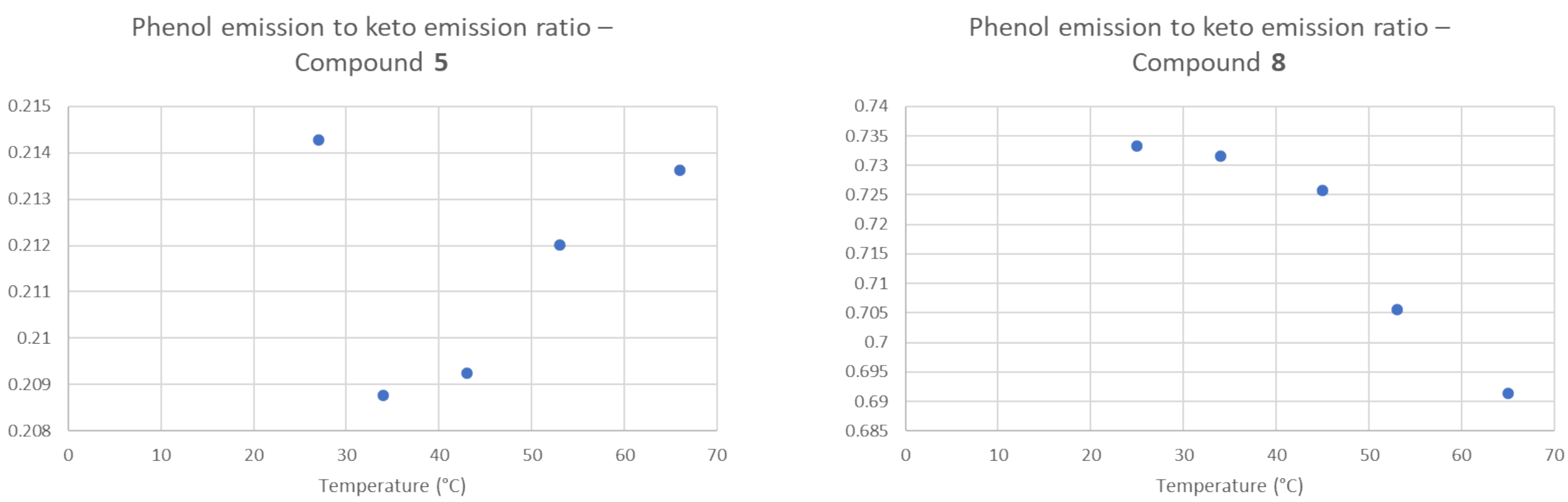

Figure S43. Ratio between the phenol and the keto emission intensities of compounds **5** and **8** measured in 2-methyl tetrahydrofuran solution (5x10^{-5} M) at different temperatures.

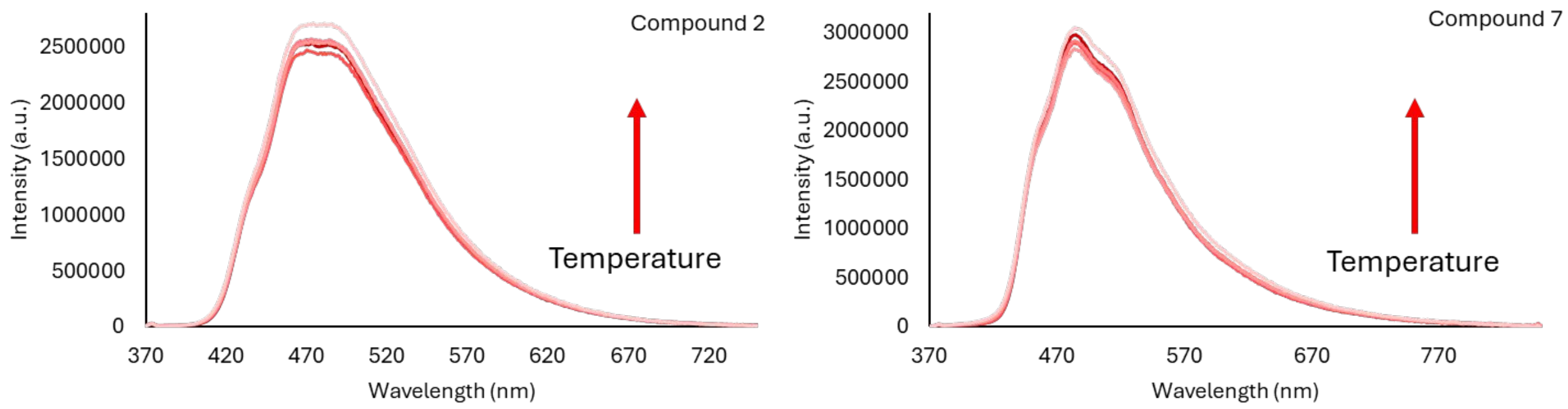

Figure S44. Emission spectra of compounds **2** and **7** measured in 2-methyl tetrahydrofuran ($5x10^{-5}$ M) at different temperatures.

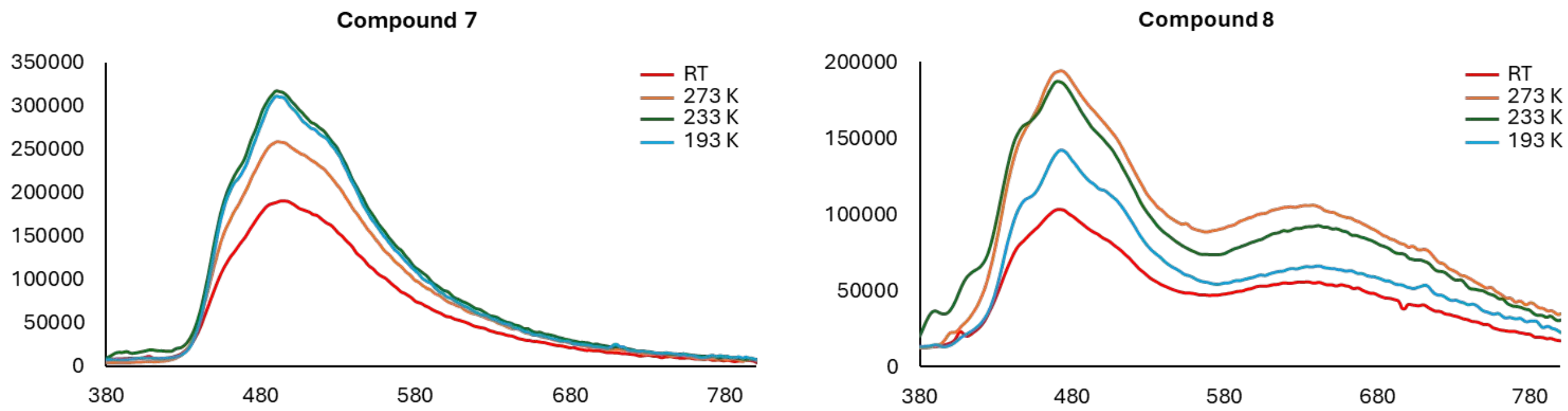

Figure S45. Emission spectra of compounds **7** and **8** measured in dichloromethane ($5x10^{-5}$ M) at low temperatures.

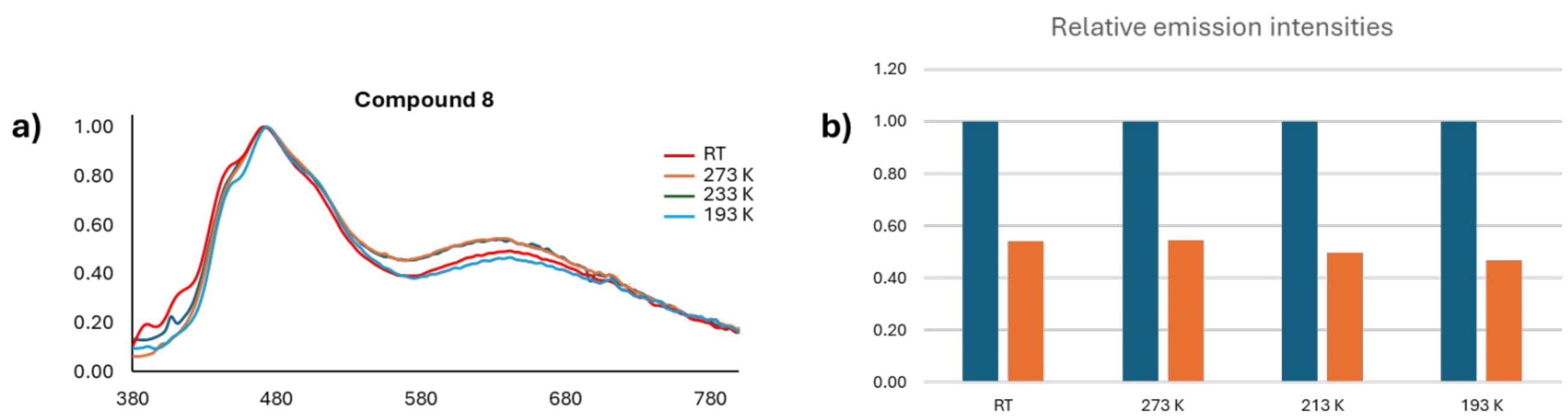

Figure S46. a) Normalized emission spectra of compounds **8** measured in dichloromethane ($5x10^{-5}$ M) at low temperatures. b) Relative emission intensities of the enol (blue, normalized) and keto (orange) forms of compound **8** measured at low temperatures.

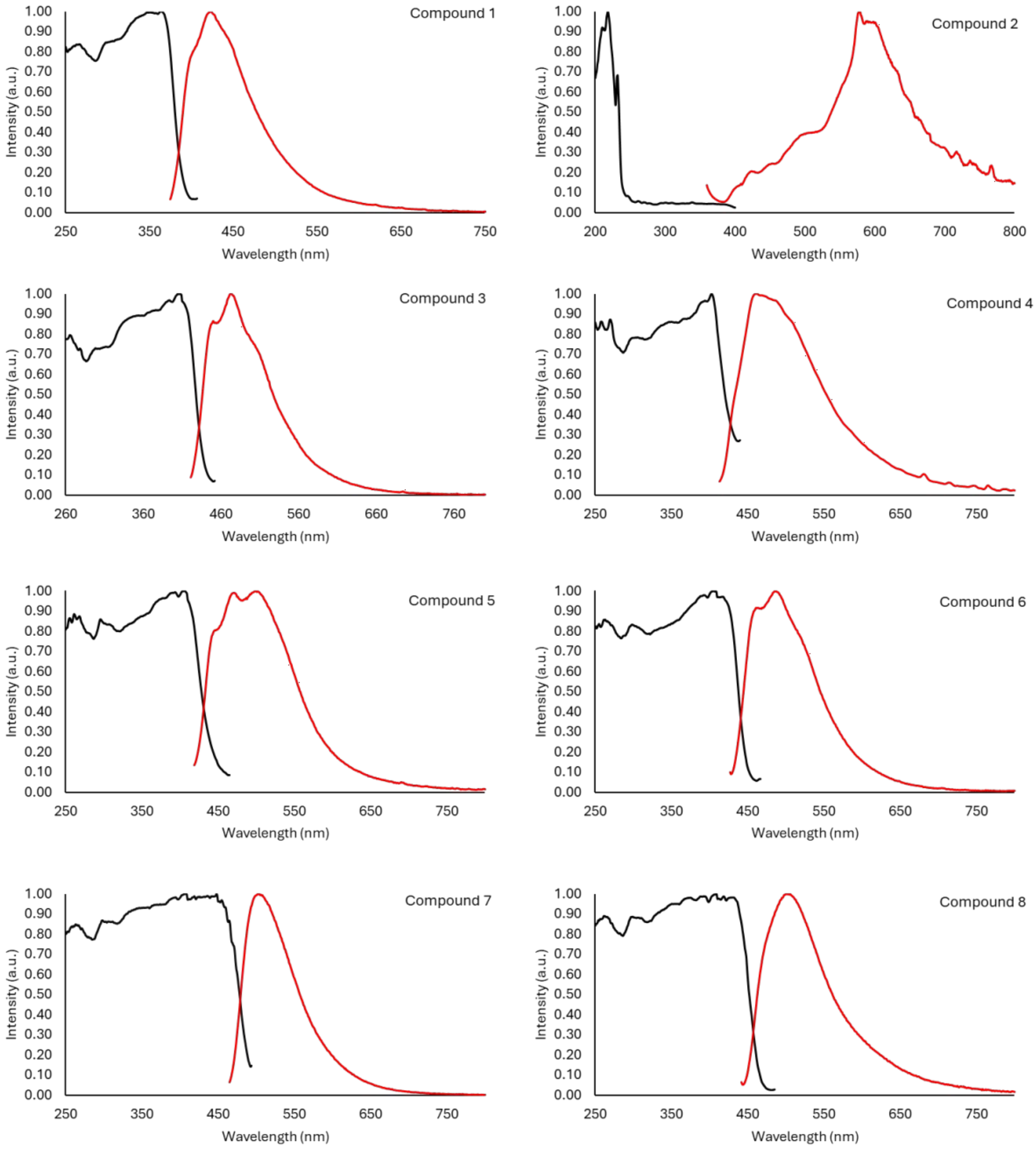

Figure S47. Normalized excitation (black) and emission (red) spectra of compounds **1**-**8** recorded in solid state.

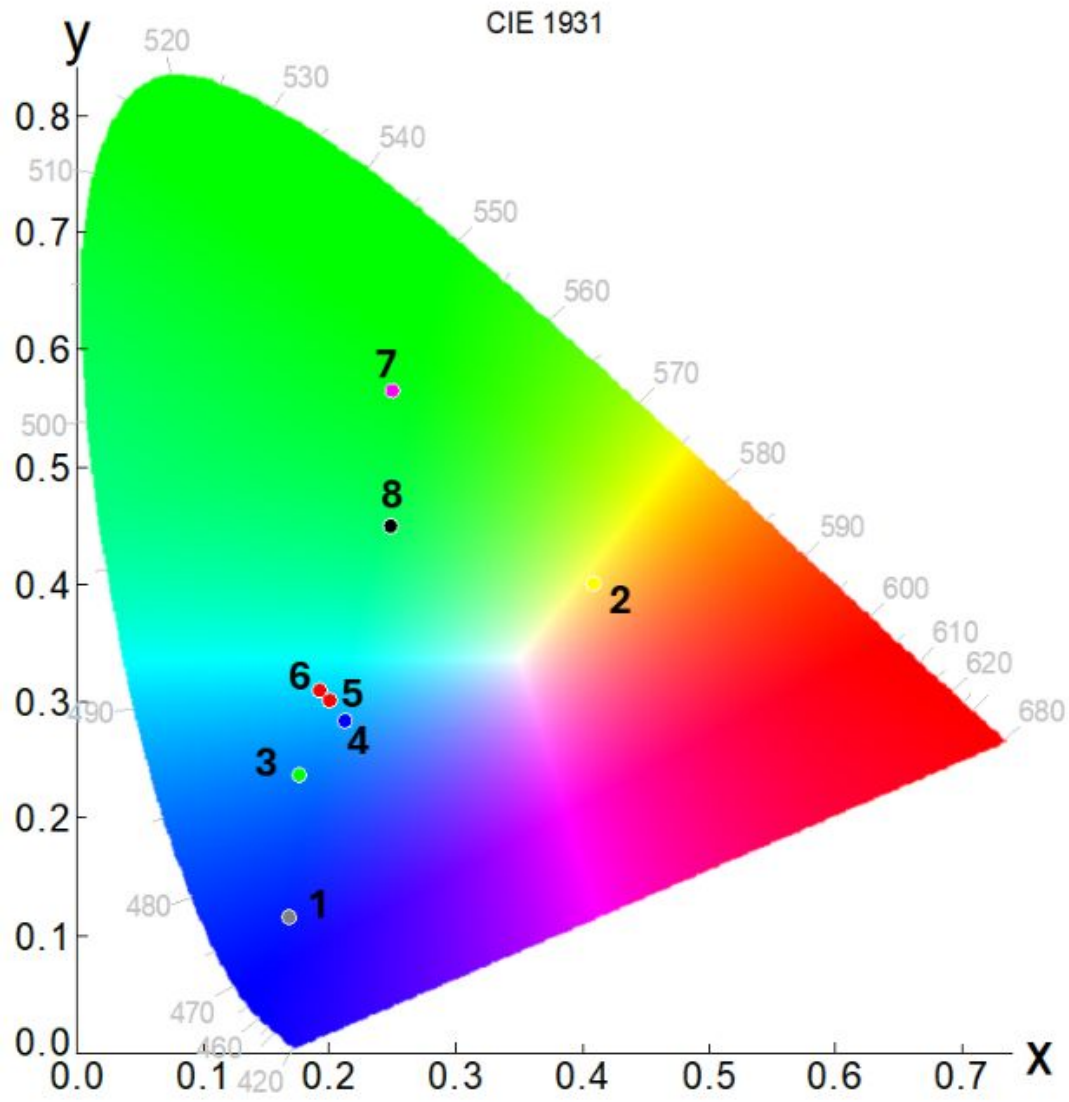

Figure S48. CIE 1931 chromaticity plot of compounds **1**-**8** recorded in solid state.

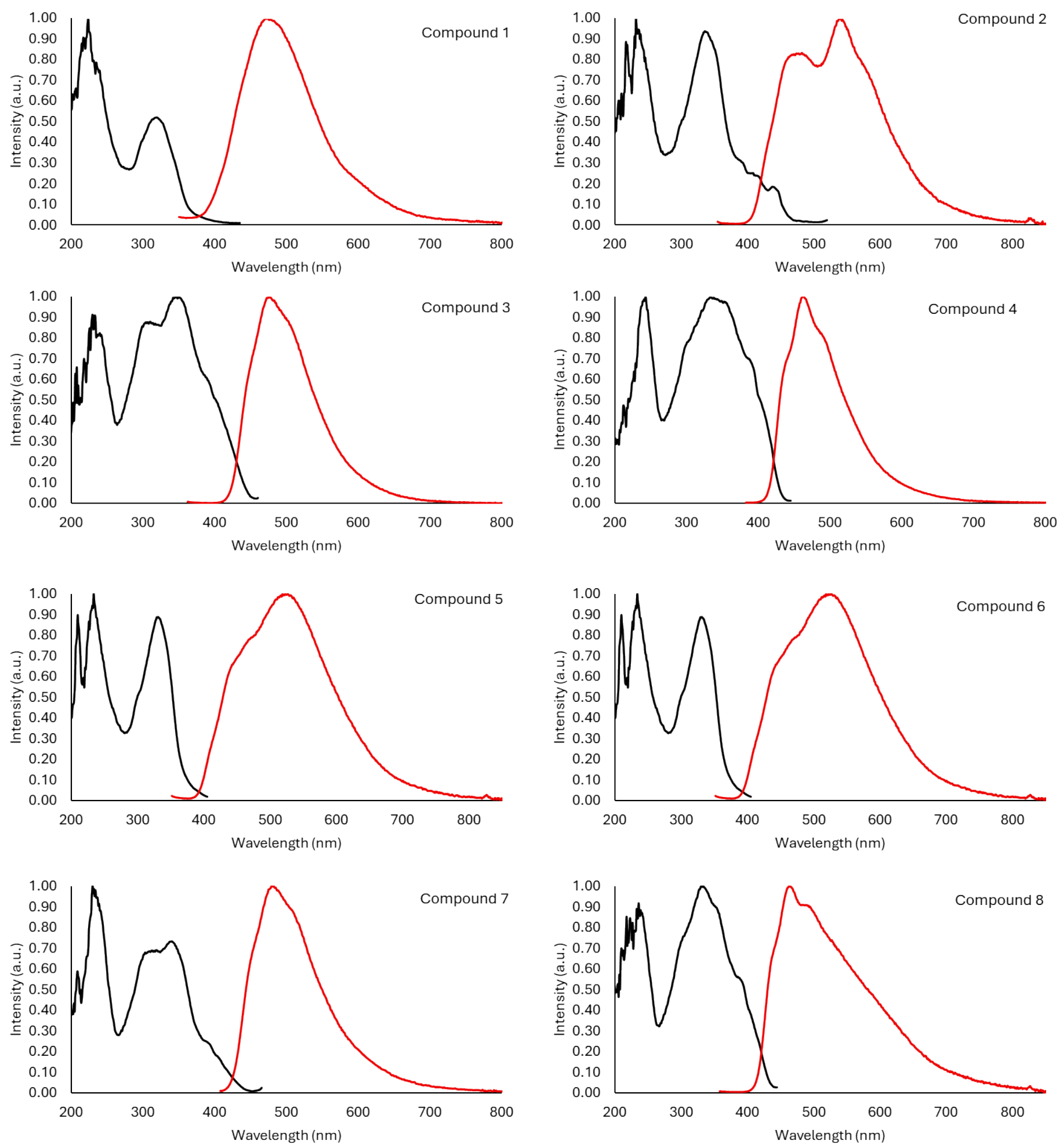

Figure S49. Normalized excitation (black) and emission (red) spectra of compounds **1**-**8** recorded in thin film using PMMA as matrix.

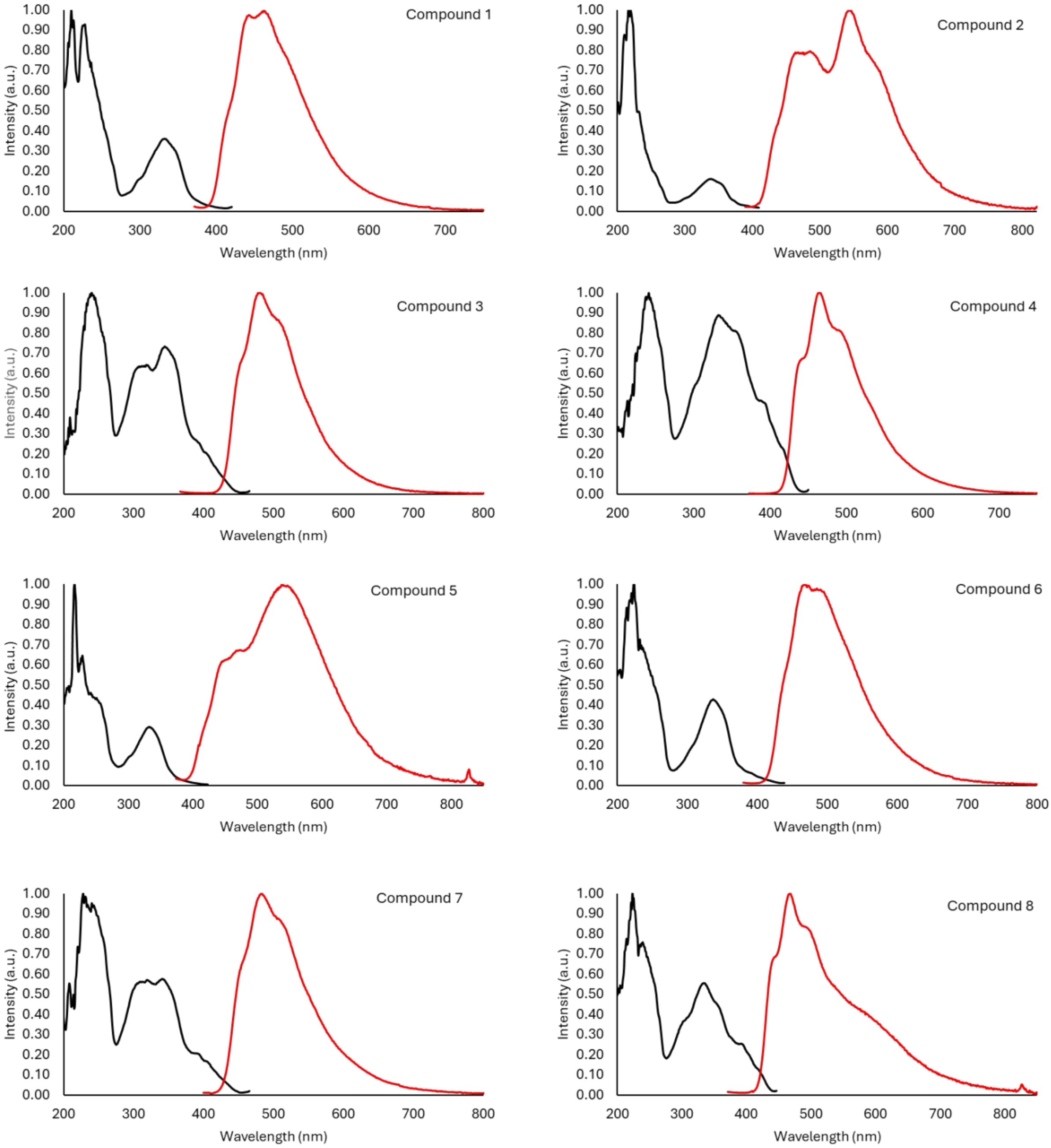

Figure S50. Normalized excitation (black) and emission (red) spectra of compounds **1**-**8** recorded in thin film using PS as matrix.

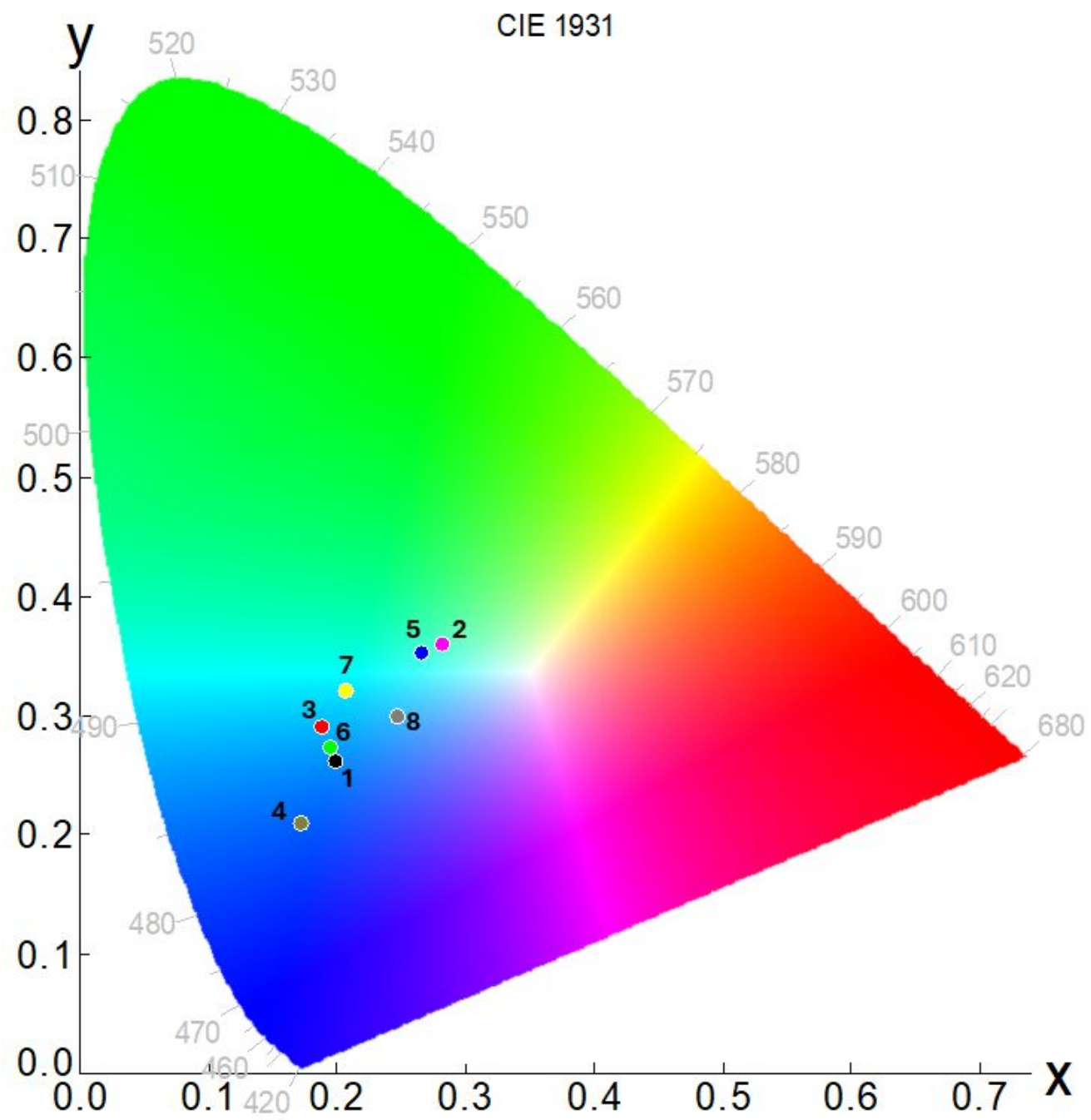

Figure S51. CIE 1931 chromaticity plot of compounds **1**-**8** recorded in thin film using PMMA as matrix.

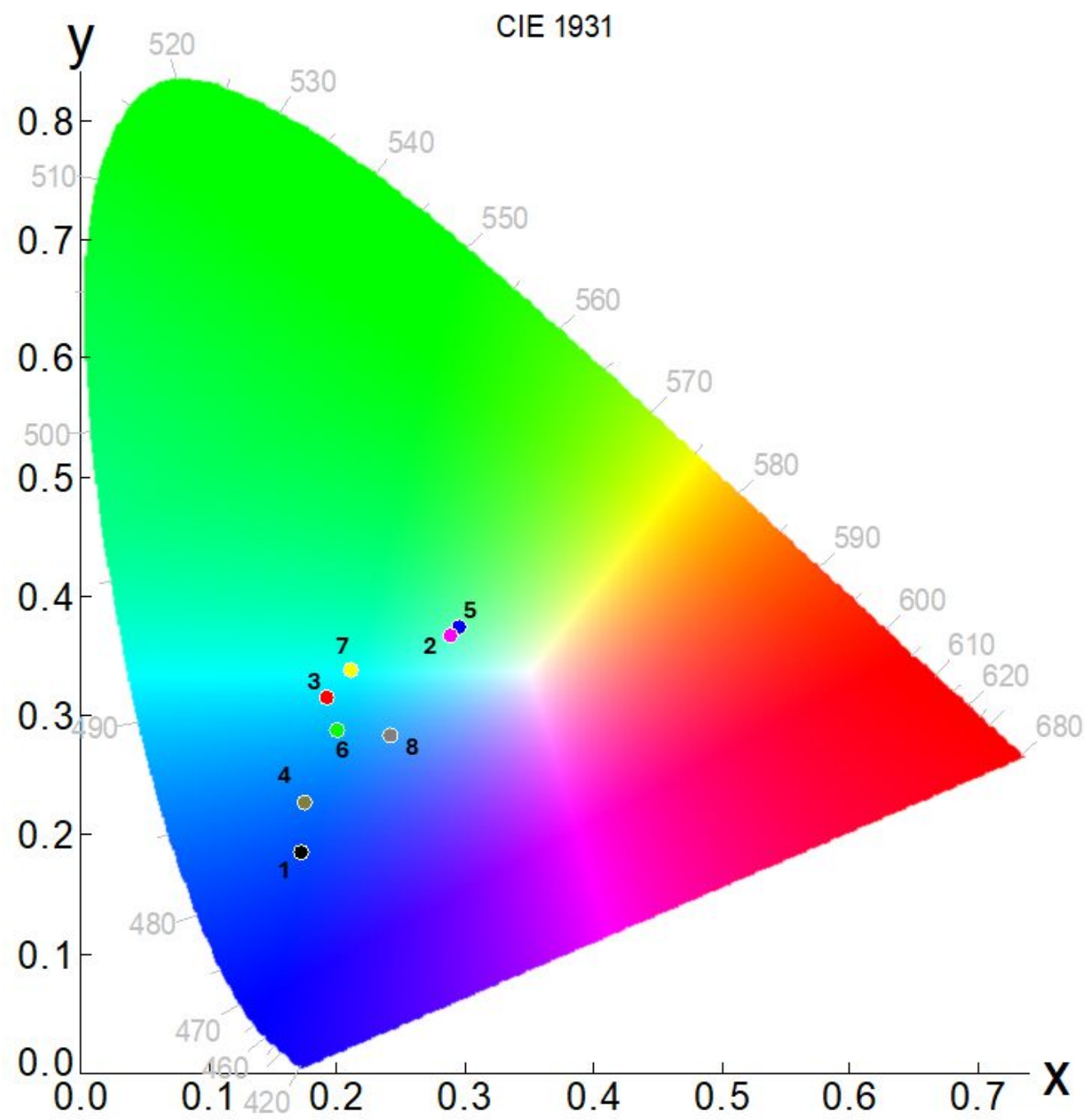

Figure S52. CIE 1931 chromaticity plot of compounds **1**-**8** recorded in thin film using PS as matrix.

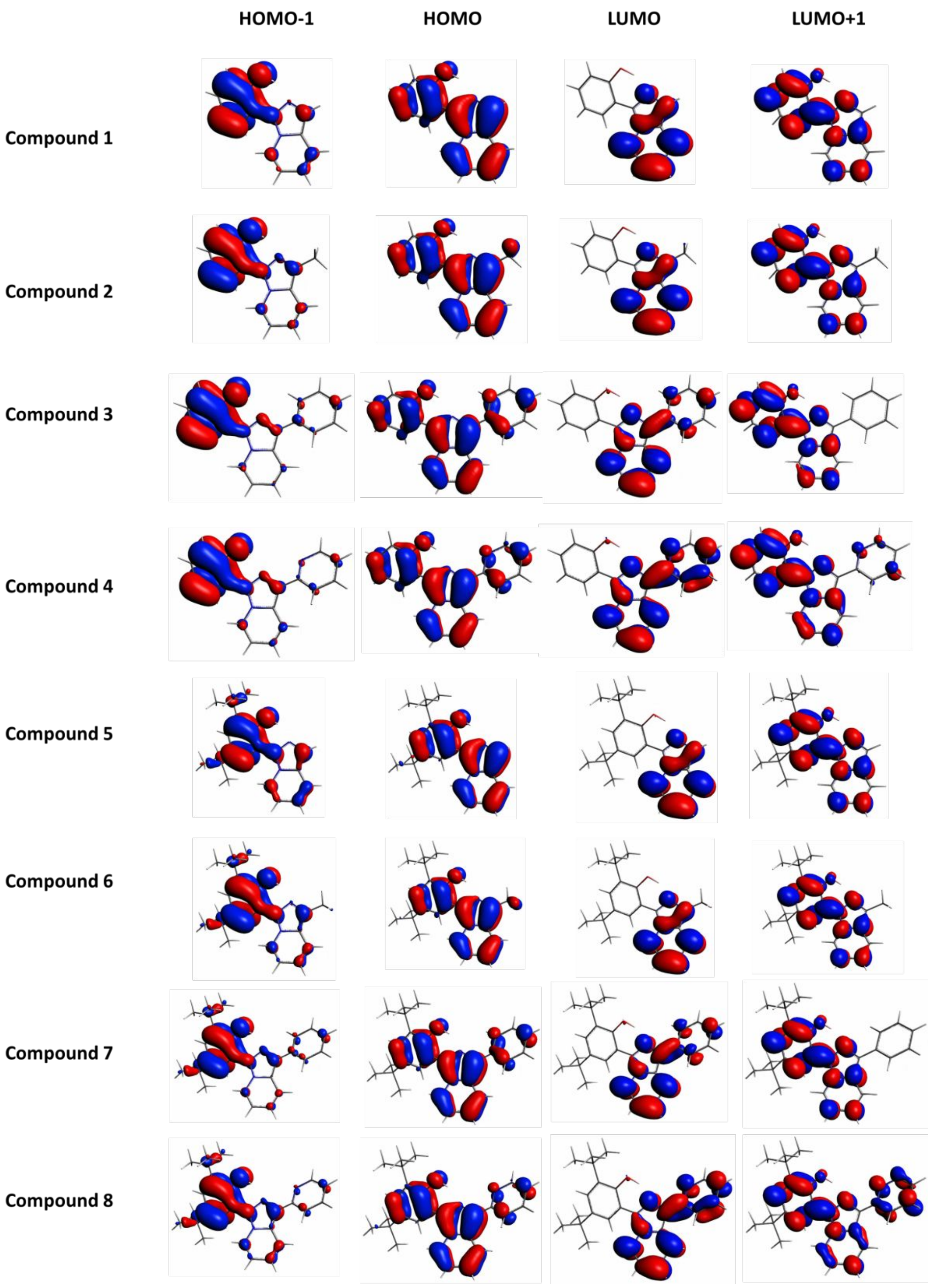

Figure S53. Frontier orbitals of compounds **1**-**8**.

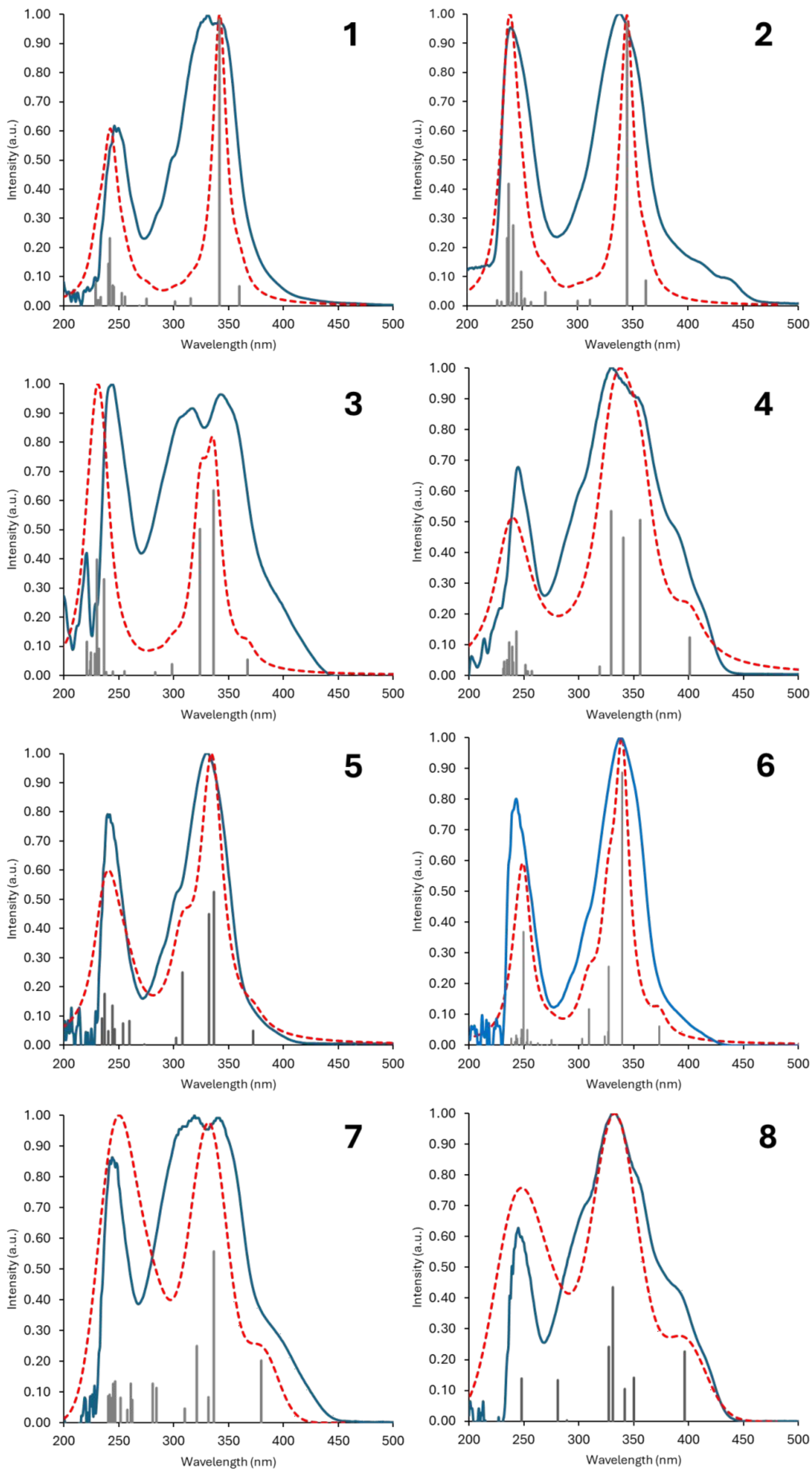

Figure S54. Experimental (solid blue line) and calculated (dashed red line) UV-vis spectra of compounds **1**-**8** in dichloromethane solution.

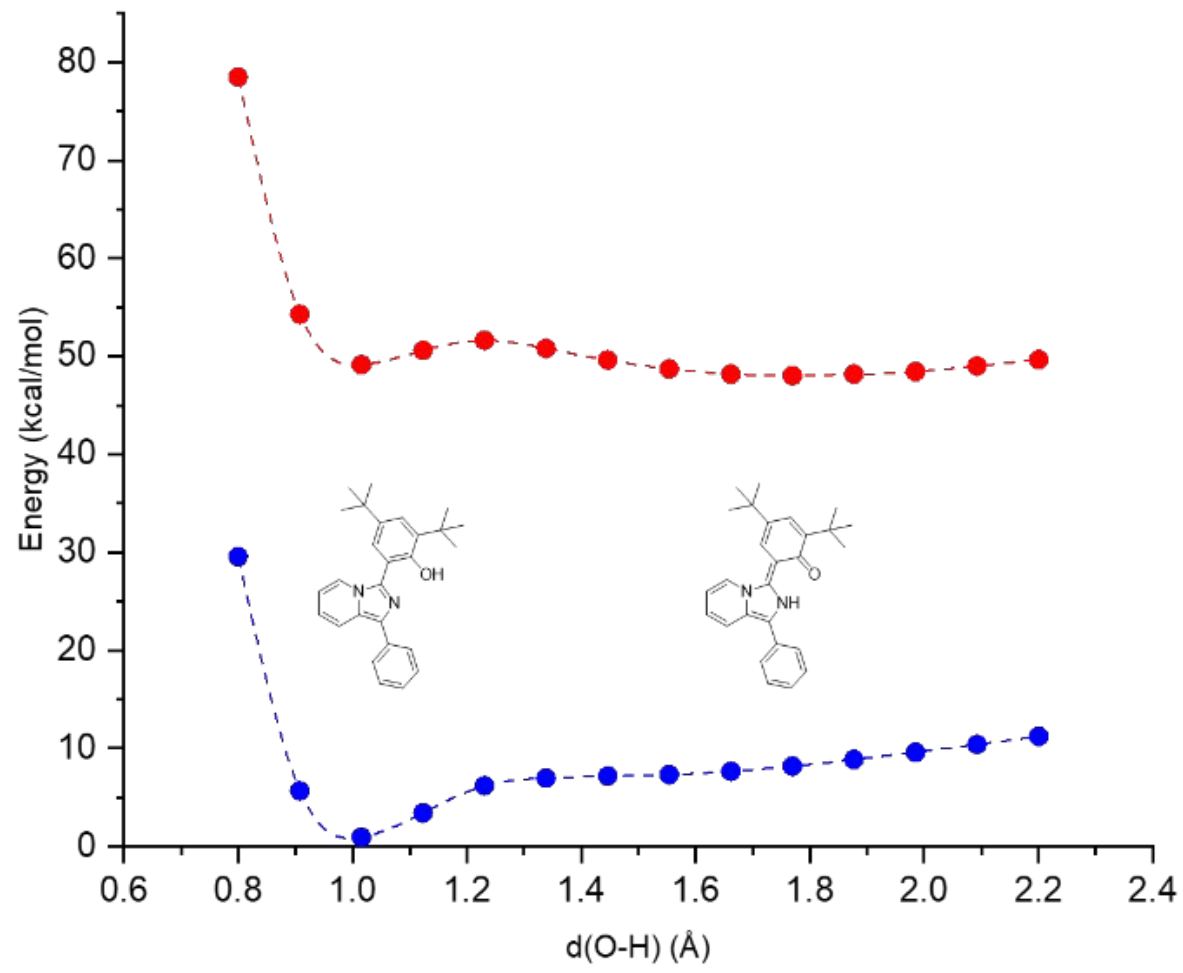

Figure S55. PES Scan curves for the enol and keto forms of compound **7** computed both in the ground and first excited state. Blue trace: ground state; red trace: first excited singlet state.

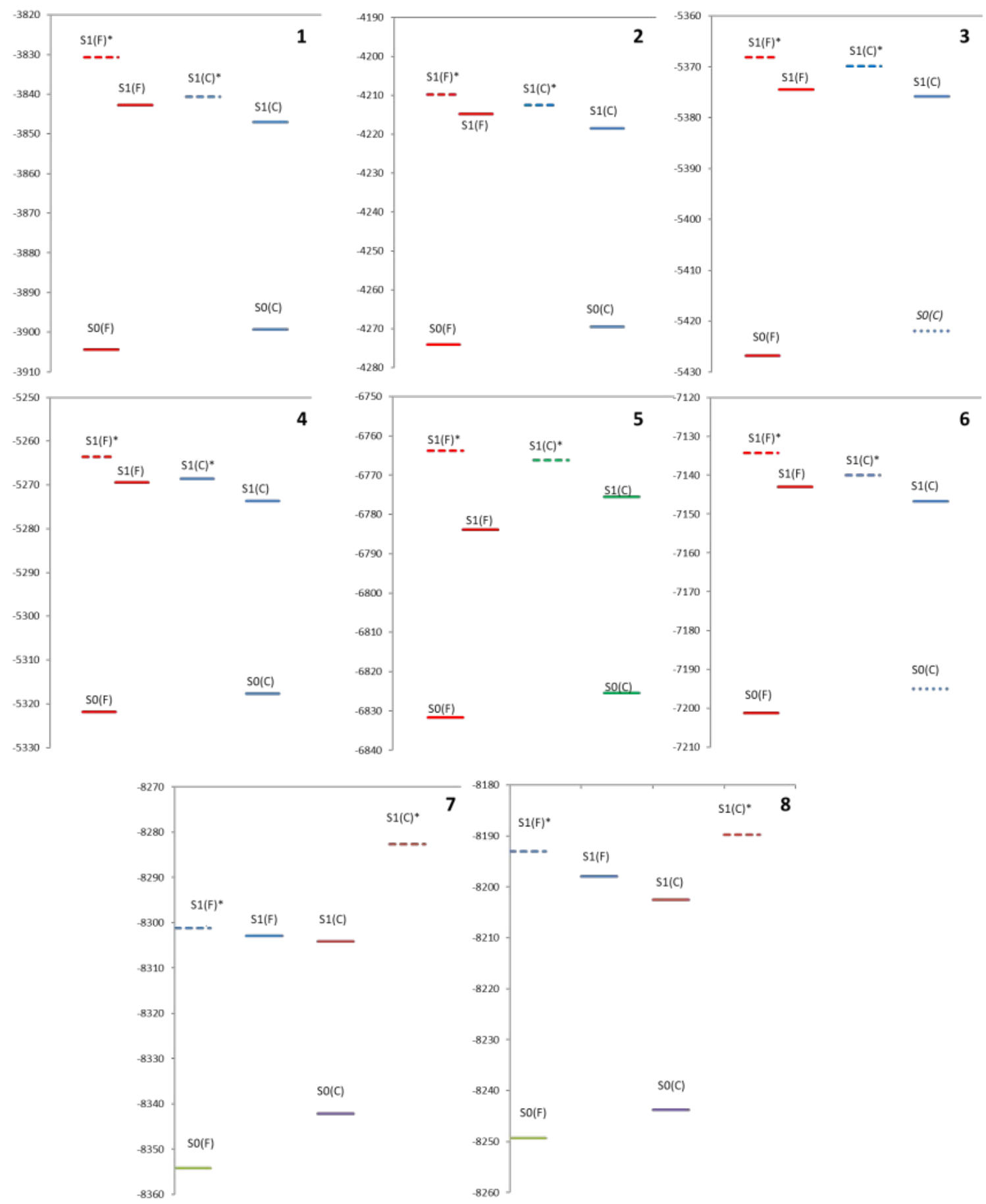

Figure S56. Computed energies of compounds **1-8** calculated at the DFT/BLYP-D3(BJ) level of theory. S0(F): phenol at the ground state; S1(F)*: phenol at the first excited state after the vertical transition; S1(F): phenol at the first excited state after internal conversion; S0(C): ketone at the ground state; S1(C)*: ketone at the first excited state right after ESIPT; S1(C): ketone at the first excited state after internal conversion.

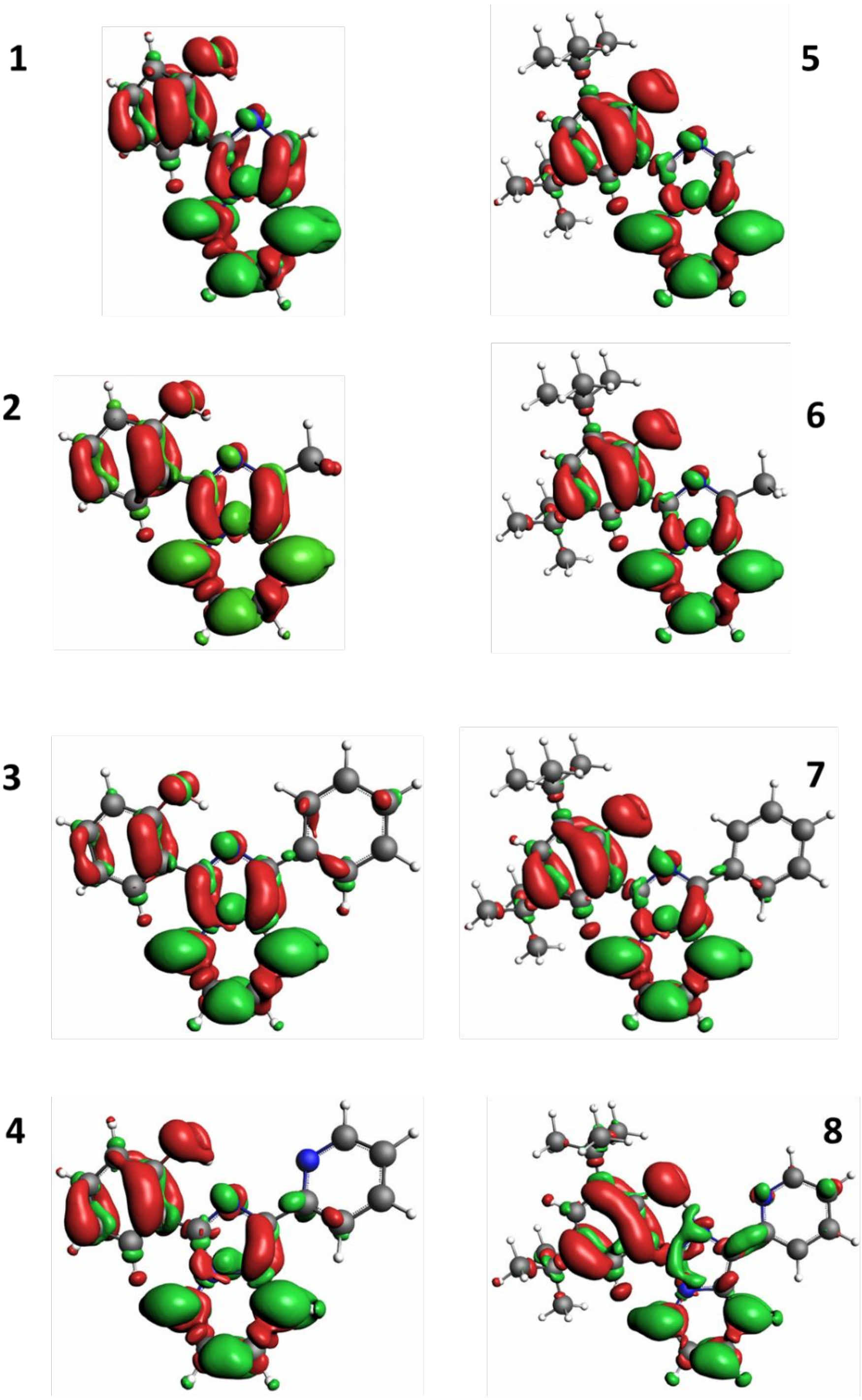

Figure S57. Electron Density Difference Maps (EDDM) for the lowest energy singlet electronic transition computed by TD-DFT (**red** indicates a decrease in electron density, **green** indicates an increase) for compounds **1**-**8**.

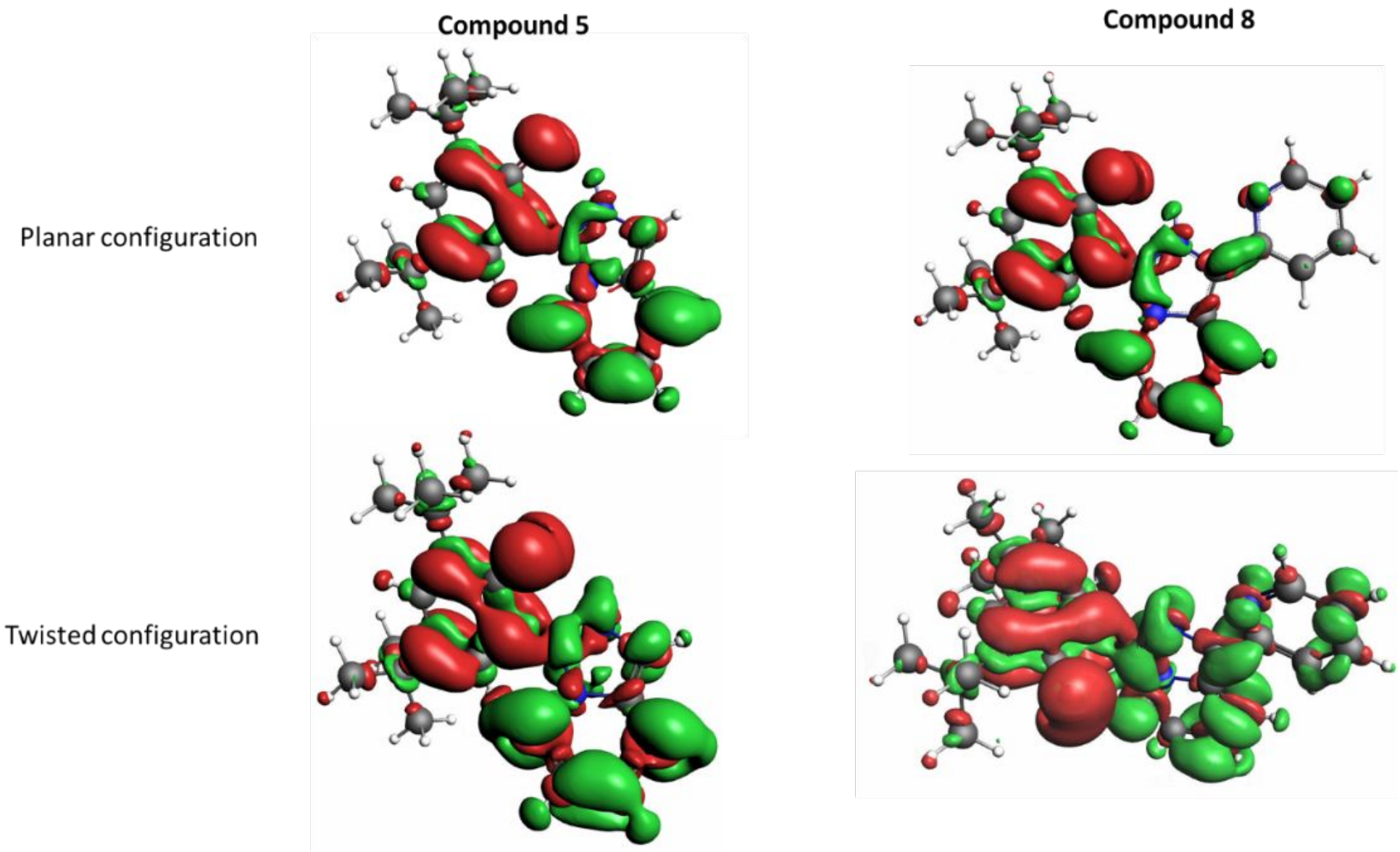

Figure S58. Electron Density Difference Maps (EDDM) for the lowest energy singlet electronic transition computed by TD-DFT (**red** indicates a decrease in electron density, **green** indicates an increase) for compounds **5** and **8** in their ketone form. Planar configuration indicates the geometry immediately after the proton transfer; twisted configuration indicates the geometry in the S_1 state after geometry optimization.

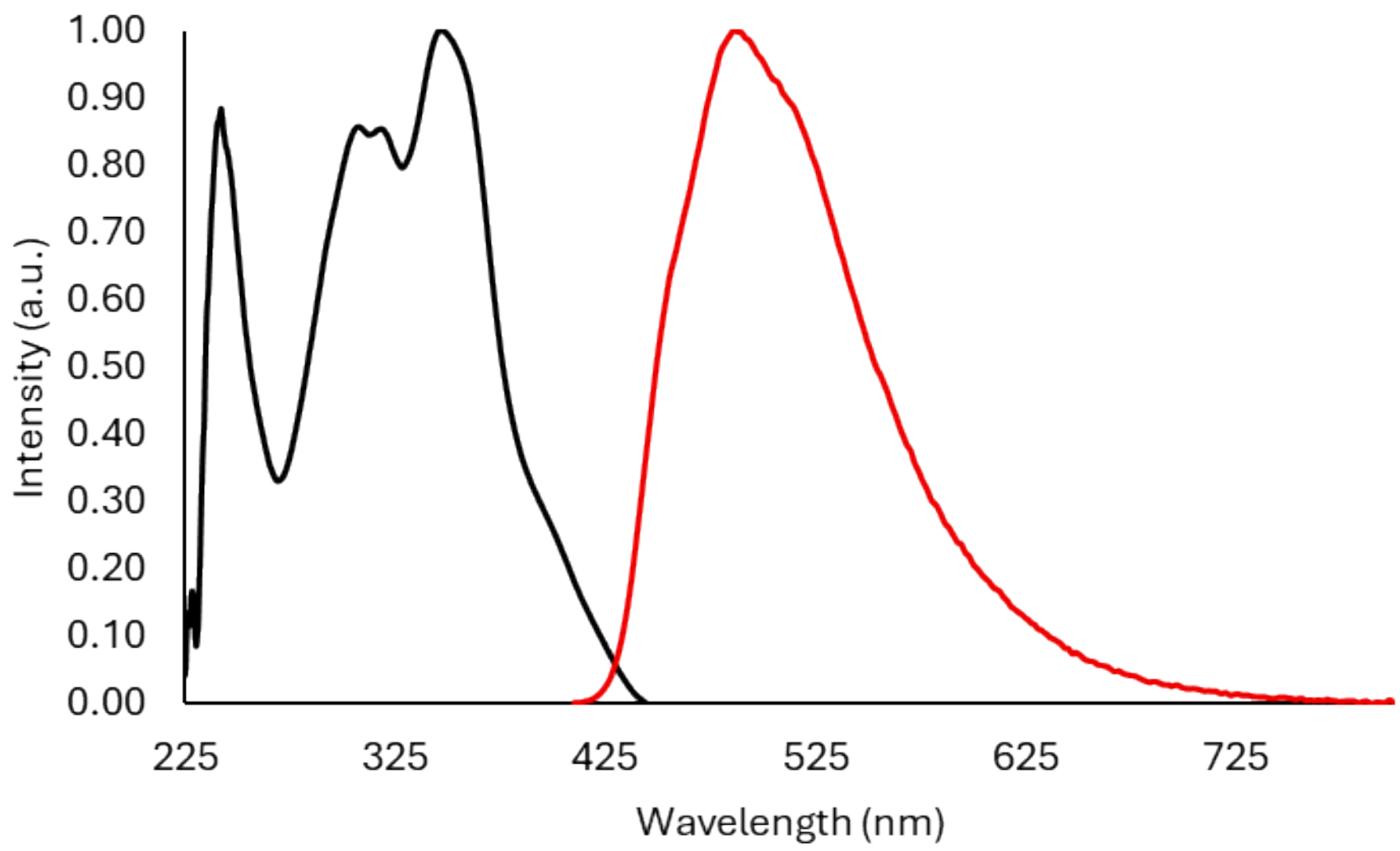

Figure S59. Normalized absorption (black) and emission (red) spectra of compound **11** recorded in dichloromethane (5x10^{-5} M).

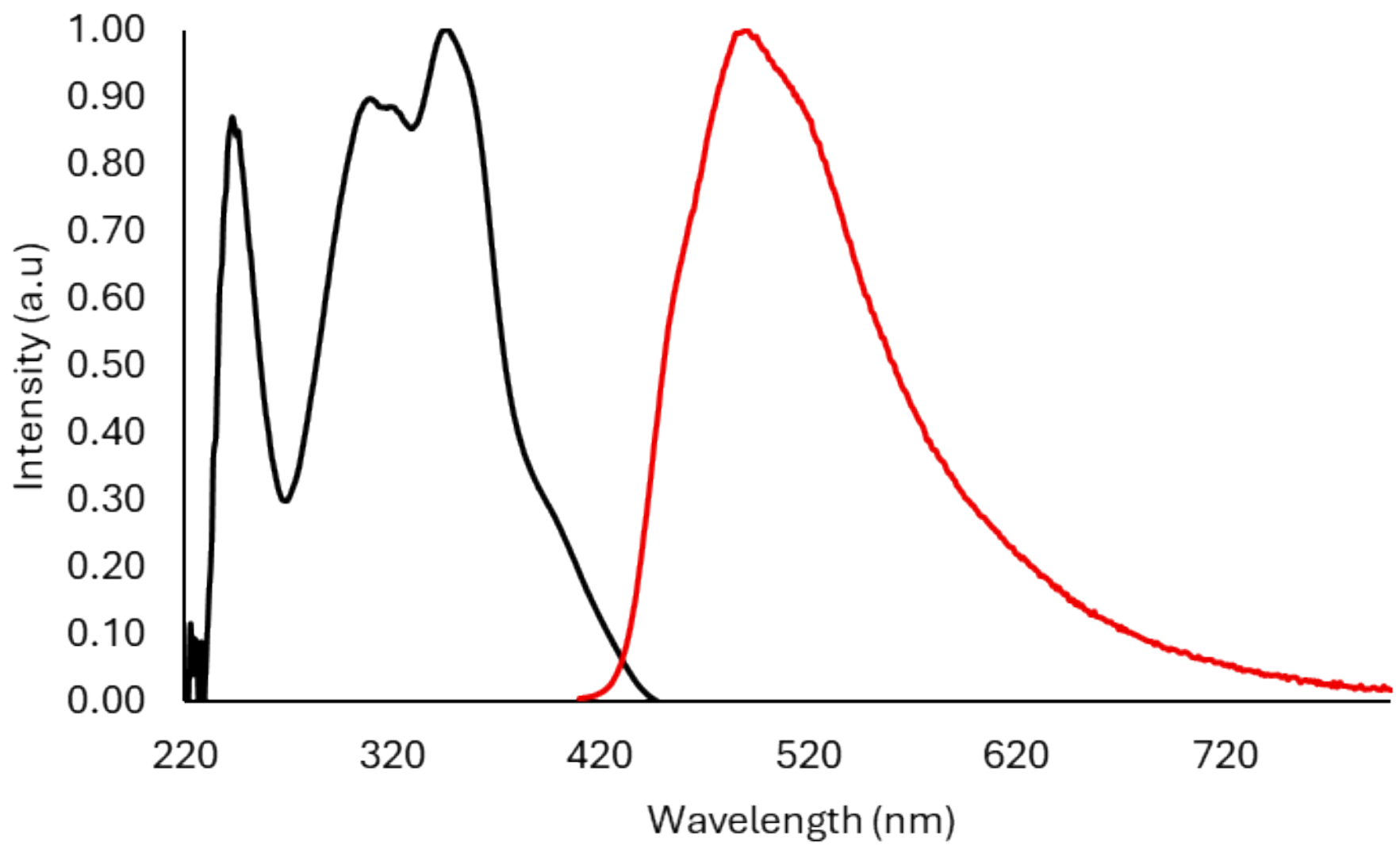

Figure S60. Normalized absorption (black) and emission (red) spectra of compound **14** recorded in dichloromethane (5x10^{-5} M).

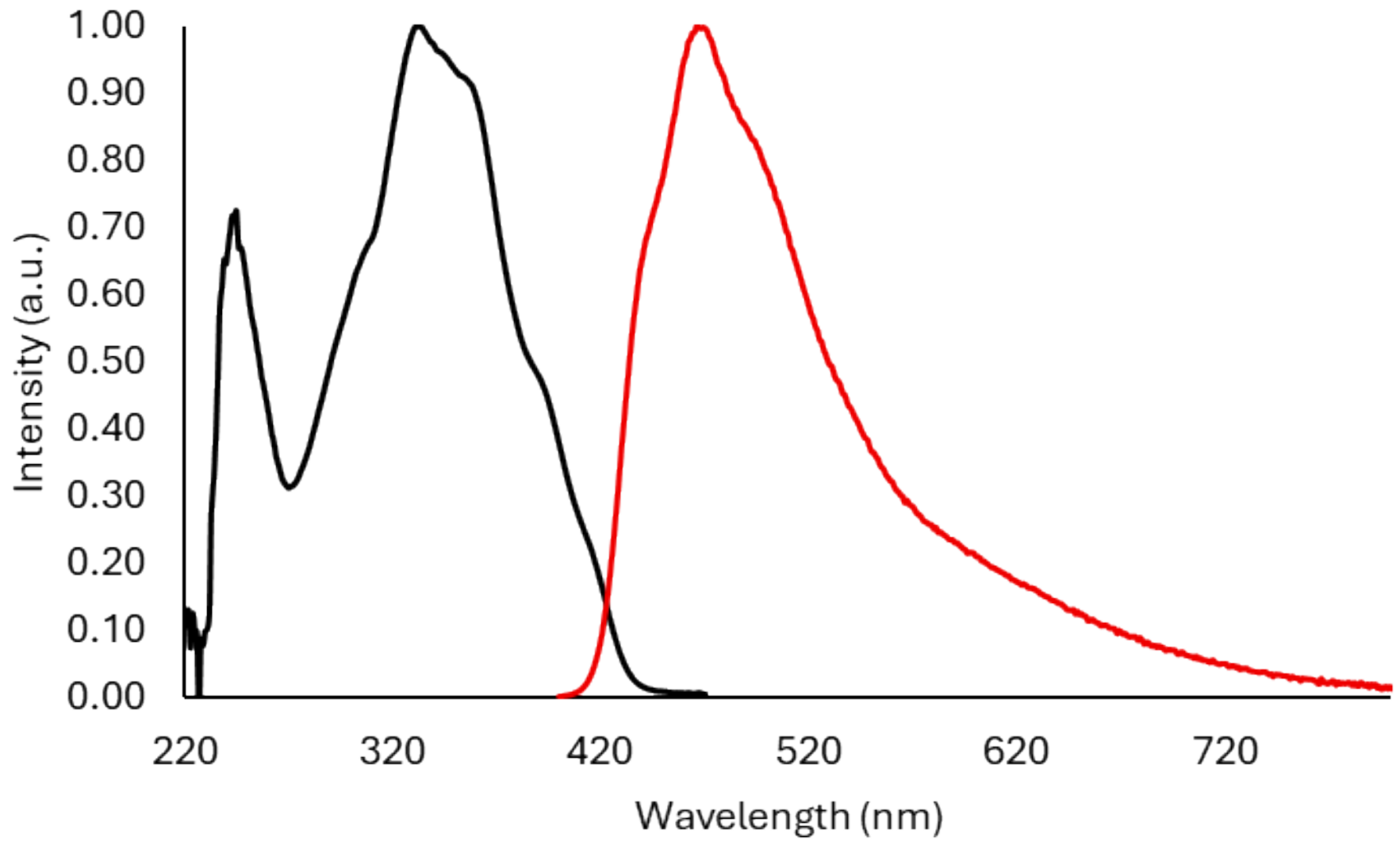

Figure S61. Normalized absorption (black) and emission (red) spectra of compound **20** recorded in dichloromethane solution (5x10^{-5} M).

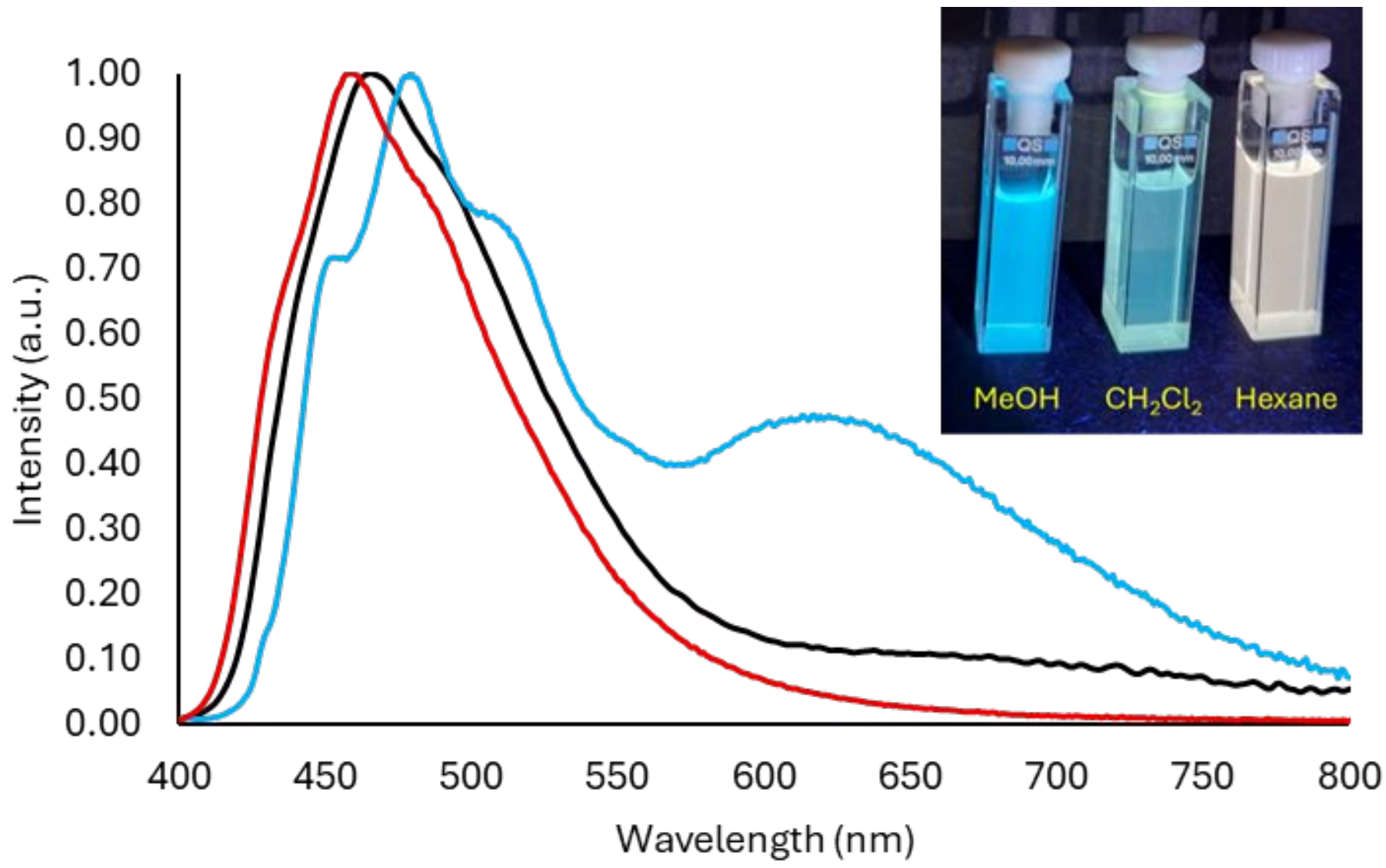

Figure S62. Normalized emission spectra of compound **15** recorded in dichloromethane (black), hexane (light blue) and methanol (red) ($5x10^{-5}$ M). Inset: picture of compound **15** emission in solution in different solvents under UV light (365 nm).

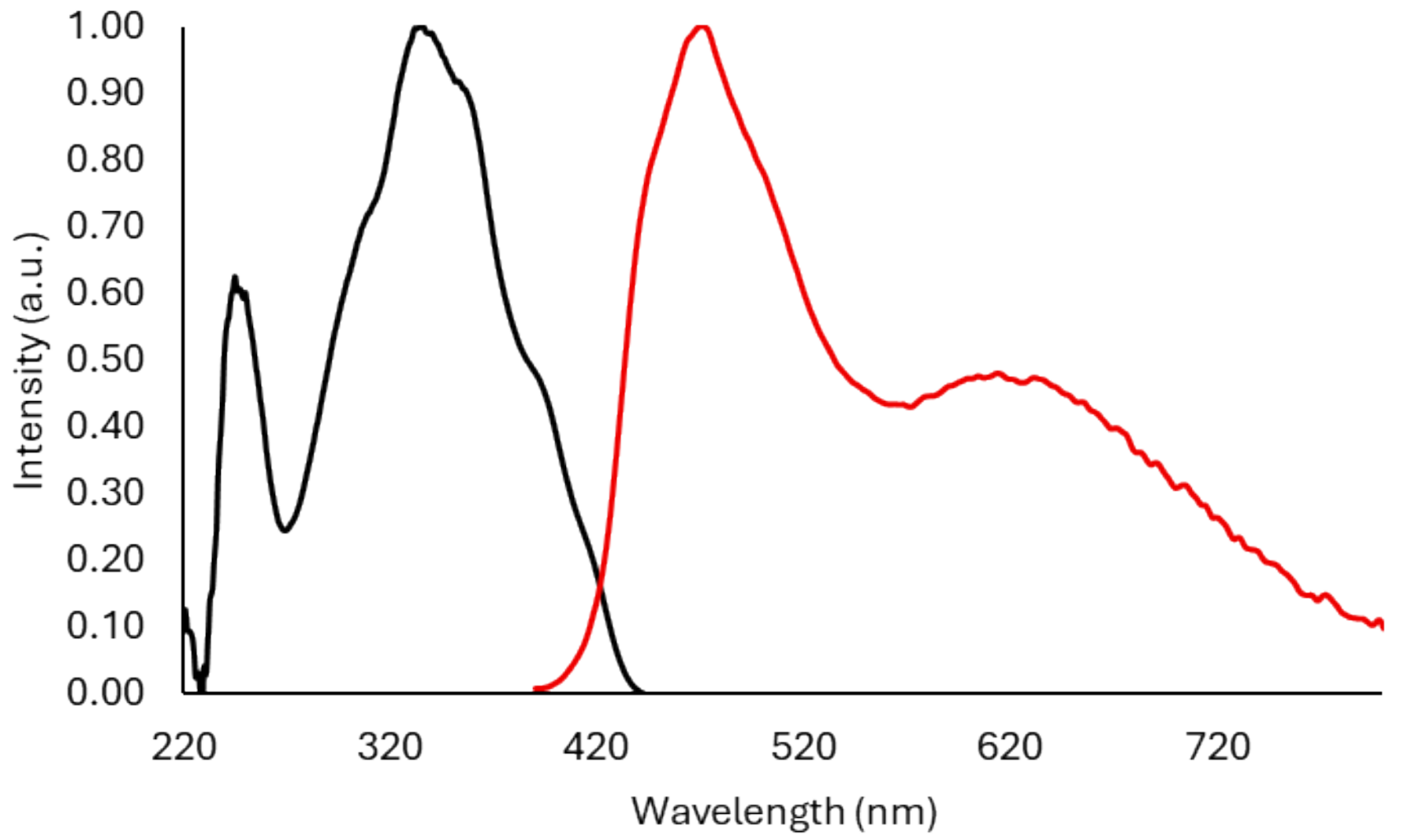

Figure S63. Normalized absorption (black) and emission (red) spectra of compound **10** recorded in dichloromethane solution ($5x10^{-5}$ M).

	**7**
Chemical formula	$C_{27}H_{30}N_2O$
Formula weight	398.552
Crystal system	Triclinic
Space group	*P* -1 (no. 2)
Crystal color and shape	Green block
Crystal size	0.70 x 0.65 x 0.56
a (Å)	7.0826(5)
b (Å)	9.5577(6)
c (Å)	17.9941(12)
α (°)	103.459(5)
β (°)	91.190(5)
γ (°)	107.734(5)
V (Å^3)	1122.80(14)
Z	2
T (K)	250(2)
D_c (g·cm^{-3})	1.179
μ (mm^{-1})	0.071
Scan range (°)	2.31 < θ < 26.31
Unique reflections	4482
Observed refls [I>2σ(I)]	3822
R_{int}	0.0204
Final *R* indices [I>2σ(I)]*	0.0447, wR_2 0.1191
R indices (all data)	0.0517, wR_2 0.1281
Goodness-of-fit	1.0787
Max, Min Δρ/e (Å^{-3})	0.2546, - 0.1811
CCDC Number	2413876

* Structures were refined on F_0^2: $wR_2 = [\Sigma[w(F_0^2 - F_c^2)^2] / \Sigma w(F_0^2)^2]^{1/2}$, where $w^{-1} = [\Sigma(F_0^2) + (aP)^2 + bP]$ and $P = [\max(F_0^2, 0) + 2F_c^2]/3$

Table S1. Crystallographic and structure refinement parameters for compound **7**.

**Solvent**	**λ_{abs} (nm)**	**λ_{exc} (nm)**	**λ_{em} (nm)**	**Φ_{PL}**	**τ (ns)**
Dichloromethane	333	353	471, 628	< 0.05	0.27 (84.3%) 6.27 (15,7%)
Acetone	333	353	461	< 0.05	2.04
Acetonitrile	330	351	467	< 0.05	0.29 (85.94%) 5.33 (14.06%)
Isopropanol	330	354	463, 601	< 0.05	0.36 (98.75%) 6.10 (1.25%)
Methanol	327	368	451	< 0.05	0.22 (97.64%) 5.13 (2.36%)
Tetrahydrofurane	331	334	465, 640	< 0.05	0.32 (96.05%) 5.96 (3.95%)
Toluene	334	336	469, 580	0.10	1.58

Table S2. Photophysical data of compound **8** recorded in solution ($5 \cdot 10^{-5}$ M) in different solvents.

	R_1	R_2	τ standard emission (ns)	τ ESIPT emission (ns)
**5**	H	*t*Bu	0.50 (33.38 %) 4.70 (66.62 %)	0.34 (88.39%) 5.44 (11.61%)
**8**	Py	*t*Bu	0.27 (84.3 %) 6.27 (15.7 %)	0.25 (97.83%) 5.09 (2.17%)

Table S3. Lifetime decays comparison between those measured at the standard emission and those measured at the ESIPT emission, in dichloromethane solution (5×10^{-5} M).

Compound	Experimental Emission			Calculated emission		
	Phenol	Keto	Anion	Phenol	Keto	Anion
**1**	464	/		443	/	
**2**	472	/		471	/	
**3**	491	/		490	/	
**4**	469	/		474	/	
**5**	472	598	580	452	517	528
**6**	490	/		477	/	
**7**	496	/		490	/	
**8**	471	622	611	488	617	598

Table S4. Comparison between experimental and calculated emission wavelengths of compounds **1**-**8**.

	R1	R2	Polymer	λ_{exc} (nm)	λ_{em} (nm)	Φ_{PL}	τ (ns)
**1**	H	H	PMMA	319	475	0.14	2.08 (41.11 %)<br>3.80 (33.69 %)
			PS	332	462	0.10	3.15
**2**	Me	H	PMMA	336	474, 540	0.07	1.39 (78.91 %)<br>3.48 (21.09 %)
			PS	333	485, 543	0.08	1.35 (72.28 %)<br>3.06 (27.72 %)
**3**	Ph	H	PMMA	348	476	0.23	2.30 (41.63 %)<br>4.78 (58.37 %)
			PS	344	480	0.11	2.23 (26.80 %)<br>4.41 (73.20 %)
**4**	Py	H	PMMA	334	462	0.46	1.57 (48.87 %)<br>3.39 (51.13 %)
			PS	333	464	0.09	1.80 (43.33 %)<br>3.43 (56.67 %)
**5**	H	*t*Bu	PMMA	331	524	0.45	1.67 (53.55 %)<br>3.37 (46.45 %)
			PS	333	473, 543	< 0.05	2.10 (74.04 %)<br>4.31 (25.96 %)
**6**	Me	*t*Bu	PMMA	336	477	< 0.05	1.90 (52.70 %)<br>3.58 (47.30 %)
			PS	337	467	< 0.05	1.98 (48.47 %)<br>3.62 (51.53 %)
**7**	Ph	*t*Bu	PMMA	340	480	0.32	1.94 (48.33 %)<br>4.18 (51.67 %)
			PS	341	483	0.06	2.49 (41.52 %)<br>4.53 (58.48 %)
**8**	Py	*t*Bu	PMMA	332	468. 569	0.39	1.11 (59.73 %)<br>2.76 (40.27 %)
			PS	334	476	0.12	1.41 (62.36 %)<br>3.08 (37.64 %)

Table S5. Photophysical data of compounds **1**-**8** recorded in thin film with polymeric matrixes.

Compound	E (S_0)	E (S_1)
**1**	-6.22204	-6.12109
**2**	-6.81129	-6.71395
**3**	-8.64826	-8.55528
**4**	-8.48102	-8.38755
**5**	-10.8869	-10.8108
**6**	-11.4758	-11.3832
**7**	-13.3133	-13.2244
**8**	-13.1461	-13.0861
**9**	-7.97718	-7.87387
**10**	-9.65319	-9.5920
**11**	-9.23389	-9.14595
**12**	-14.8876	-14.8104
**13**	-11.349	-11.2618
**14**	-9.82046	-9.73352
**15**	-13.7755	-13.7141
**16**	-13.3238	-13.2565
**17**	-10.8111	-10.7223
**18**	-10.8156	-10.7504
**19**	-9.06765	-8.97847
**20**	-9.06662	-8.97923
**21**	-9.23486	-9.14595
**22**	-10.9829	-10.8920
**23**	-10.9783	-10.8865
**24**	-7.85606	-7.78834
**25**	-7.01938	-6.94616
**26**	-7.02449	-6.95322
**27**	-7.82192	-7.75739

Table S6. Total energies (Hartree) for ground and first excited singlet state, computed at the DFT/BLYP-D3(BJ) level of theory for compounds **1**-**24**.

Compound	O-H length (Å)	H---N length (Å)	O-H-N angle (°)
**1**	1.011	1.696	149.5
**2**	1.013	1.683	149.7
**3**	1.013	1.685	149.5
**4**	1.013	1.688	149.1
**5**	1.008	1.698	150.7
**6**	1.001	1.684	151.0
**7**	1.011	1.676	150.9
**8**	1.011	1.675	150.4

Table S7. Calculated hydrogen bond parameters of compounds **1**-**8**.

	Transition	Percentage
**1**	HOMO → LUMO	93.67 %
	HOMO-1 → LUMO	3.57 %
	HOMO-2 → LUMO	0.91 %
**2**	HOMO → LUMO	94.82 %
	HOMO-1 → LUMO	2.29 %
	HOMO-2 → LUMO	0.74 %
**3**	HOMO → LUMO	95.89 %
	HOMO-1 → LUMO	1.32 %
	HOMO → LUMO+1	0.79 %
**4**	HOMO → LUMO	95.34 %
	HOMO-1 → LUMO	2.16 %
	HOMO → LUMO+1	0.74 %
**5**	HOMO → LUMO	91.48 %
	HOMO-1 → LUMO	6.54 %
	HOMO-2 → LUMO	0.77 %
**6**	HOMO → LUMO	93.27 %
	HOMO-1 → LUMO	4.35 %
	HOMO-2 → LUMO	0.69 %
	HOMO → LUMO+1	0.45 %
**7**	HOMO → LUMO	94.28 %
	HOMO-1 → LUMO	3.69 %
	HOMO-4 → LUMO	0.52 %
	HOMO → LUMO+1	0.36 %
**8**	HOMO → LUMO	92.92 %
	HOMO-1 → LUMO	5.43 %
	HOMO → LUMO+1	0.26 %

Table S8. Most important contribution of single orbital transitions to lower energy absorption of compounds **1**-**8**.

Entry	$f_{(ESIPT)}$	*o*-*t*Bu	*p*-*t*Bu	*o*-Me	*p*-Me	*o*-H	*p*-H	H	Me	Ph	Py	CN	*p*-CNPh	C_6F_5	*p*-N(Me)$_2$Ph	*o*-OMe	*p*-OMe
**1**	0	0	0	0	0	1	1	1	0	0	0	0	0	0	0	0	0
**2**	0	0	0	0	0	1	1	0	1	0	0	0	0	0	0	0	0
**3**	0	0	0	0	0	1	1	0	0	1	0	0	0	0	0	0	0
**4**	0	0	0	0	0	1	1	0	0	0	1	0	0	0	0	0	0
**5**	1	1	1	0	0	0	0	1	0	0	0	0	0	0	0	0	0
**6**	0	1	1	0	0	0	0	0	1	0	0	0	0	0	0	0	0
**7**	0	1	1	0	0	0	0	0	0	1	0	0	0	0	0	0	0
**8**	1	1	1	0	0	0	0	0	0	0	1	0	0	0	0	0	0
**10**	1	0	0	1	1	0	0	0	0	0	1	0	0	0	0	0	0
**11**	0	0	0	0	1	1	0	0	0	1	0	0	0	0	0	0	0
**12**	0	1	1	0	0	0	0	0	0	0	0	0	0	0	1	0	0
**13**	0	1	1	0	0	0	0	0	0	0	0	1	0	0	0	0	0
**14**	0	0	0	1	1	0	0	0	0	1	0	0	0	0	0	0	0
**15**	1	1	1	0	0	0	0	0	0	0	0	0	1	0	0	0	0
**16**	1	1	1	0	0	0	0	0	0	0	0	0	0	1	0	0	0
**17**	0	1	0	0	0	0	1	0	0	0	1	0	0	0	0	0	0
**18**	1	0	1	0	0	1	0	0	0	0	1	0	0	0	0	0	0
**19**	0	0	0	1	0	0	1	0	0	0	1	0	0	0	0	0	0
**20**	0	0	0	0	1	1	0	0	0	0	0	1	0	0	0	0	0
**21**	0	0	0	1	0	0	1	0	0	1	0	0	0	0	0	0	0
**22**	0	0	1	0	0	1	0	0	0	1	0	0	0	0	0	0	0
**23**	0	1	0	0	0	0	1	0	0	1	0	0	0	0	0	0	0
**24**	1	0	0	1	1	0	0	0	0	0	0	1	0	0	0	0	0
**25**	1	0	0	0	0	0	1	0	0	0	0	0	0	0	0	1	0
**26**	1	0	0	0	0	1	0	0	0	0	0	0	0	0	0	0	1
**27**	1	0	0	0	0	0	0	0	0	0	0	0	0	0	0	1	1

Table S9. System of linear equations written in matrix form for the calculation of Ω. *p* and *o* indicate the position of the substituents with respect to the hydroxyl group, respectively; all the other entries represent the substituents on the imidazopyridine moiety.

Entry	HOMO S_0 DFT (eV)	*o*-*t*Bu	*p*-*t*Bu	*o*-Me	*p*-Me	*o*-H	*p*-H	H	Me	Ph	Py	CN	*p*-CNPh	C_6F_5	*p*-N(Me)$_2$Ph	*o*-OMe	*p*-OMe
**1**	-4.718	0	0	0	0	1	1	1	0	0	0	0	0	0	0	0	0
**2**	-4.580	0	0	0	0	1	1	0	1	0	0	0	0	0	0	0	0
**3**	-4.604	0	0	0	0	1	1	0	0	1	0	0	0	0	0	0	0
**4**	-4.702	0	0	0	0	1	1	0	0	0	1	0	0	0	0	0	0
**5**	-4.622	1	1	0	0	0	0	1	0	0	0	0	0	0	0	0	0
**6**	-4.483	1	1	0	0	0	0	0	1	0	0	0	0	0	0	0	0
**7**	-4.527	1	1	0	0	0	0	0	0	1	0	0	0	0	0	0	0
**8**	-4.615	1	1	0	0	0	0	0	0	0	1	0	0	0	0	0	0
**10**	-4.633	0	0	1	1	0	0	0	0	0	1	0	0	0	0	0	0
**11**	-4.570	0	0	0	1	1	0	0	0	1	0	0	0	0	0	0	0
**12**	-4.173	1	1	0	0	0	0	0	0	0	0	0	0	0	1	0	0
**13**	-4.970	1	1	0	0	0	0	0	0	0	0	1	0	0	0	0	0
**14**	-4.543	0	0	1	1	0	0	0	0	1	0	0	0	0	0	0	0
**15**	-4.688	1	1	0	0	0	0	0	0	0	0	0	1	0	0	0	0
**16**	-4.724	1	1	0	0	0	0	0	0	0	0	0	0	1	0	0	0
**17**	-4.671	1	0	0	0	0	1	0	0	0	1	0	0	0	0	0	0
**18**	-4.650	0	1	0	0	1	0	0	0	0	1	0	0	0	0	0	0
**19**	-4.676	0	0	1	0	0	1	0	0	0	1	0	0	0	0	0	0
**20**	-4.662	0	0	0	1	1	0	0	0	0	0	1	0	0	0	0	0
**21**	-4.580	0	0	1	0	0	1	0	0	1	0	0	0	0	0	0	0
**22**	-4.562	0	1	0	0	1	0	0	0	1	0	0	0	0	0	0	0
**23**	-4.575	1	0	0	0	0	1	0	0	1	0	0	0	0	0	0	0
**24**	-4.973	0	0	1	1	0	0	0	0	0	0	1	0	0	0	0	0
**25**	-4.736	0	0	0	0	0	1	0	0	0	0	0	0	0	0	1	0
**26**	-4.626	0	0	0	0	1	0	0	0	0	0	0	0	0	0	0	1
**27**	-4.633	0	0	0	0	0	0	0	0	0	0	0	0	0	0	1	1

Table S10. System of linear equations written in matrix form for the calculation of HOMO energies in the ground state. *p* and *o* indicate the position of the substituents with respect to the hydroxyl group, respectively; all the other entries represent the substituents on the imidazopyridine moiety.

Entry	LUMO S_0 DFT (eV)	*o*-*t*Bu	*p*-*t*Bu	*o*-Me	*p*-Me	*o*-H	*p*-H	H	Me	Ph	Py	CN	*p*-CNPh	C_6F_5	*p*-$N(Me)_2$Ph	*o*-OMe	*p*-OMe
**1**	-2.011	0	0	0	0	1	1	1	0	0	0	0	0	0	0	0	0
**2**	-1.963	0	0	0	0	1	1	0	1	0	0	0	0	0	0	0	0
**3**	-2.165	0	0	0	0	1	1	0	0	1	0	0	0	0	0	0	0
**4**	-2.278	0	0	0	0	1	1	0	0	0	1	0	0	0	0	0	0
**5**	-1.993	1	1	0	0	0	0	1	0	0	0	0	0	0	0	0	0
**6**	-1.958	1	1	0	0	0	0	0	1	0	0	0	0	0	0	0	0
**7**	-2.157	1	1	0	0	0	0	0	0	1	0	0	0	0	0	0	0
**8**	-2.265	1	1	0	0	0	0	0	0	0	1	0	0	0	0	0	0
**10**	-2.259	0	0	1	1	0	0	0	0	0	1	0	0	0	0	0	0
**11**	-2.155	0	0	0	1	1	0	0	0	1	0	0	0	0	0	0	0
**12**	-2.008	1	1	0	0	0	0	0	0	0	0	0	0	0	1	0	0
**13**	-2.467	1	1	0	0	0	0	0	0	0	0	1	0	0	0	0	0
**14**	-2.144	0	0	1	1	0	0	0	0	1	0	0	0	0	0	0	0
**15**	-2.568	1	1	0	0	0	0	0	0	0	0	0	1	0	0	0	0
**16**	-2.274	1	1	0	0	0	0	0	0	0	0	0	0	1	0	0	0
**17**	-2.262	1	0	0	0	0	1	0	0	0	1	0	0	0	0	0	0
**18**	-2.273	0	1	0	0	1	0	0	0	0	1	0	0	0	0	0	0
**19**	-2.267	0	0	1	0	0	1	0	0	0	1	0	0	0	0	0	0
**20**	-2.270	0	0	0	1	1	0	0	0	0	0	1	0	0	0	0	0
**21**	-2.154	0	0	1	0	0	1	0	0	1	0	0	0	0	0	0	0
**22**	-2.164	0	1	0	0	1	0	0	0	1	0	0	0	0	0	0	0
**23**	-2.150	1	0	0	0	0	1	0	0	1	0	0	0	0	0	0	0
**24**	-2.463	0	0	1	1	0	0	0	0	0	0	1	0	0	0	0	0
**25**	-2.021	0	0	0	0	0	1	0	0	0	0	0	0	0	0	1	0
**26**	-2.003	0	0	0	0	1	0	0	0	0	0	0	0	0	0	0	1
**27**	-2.012	0	0	0	0	0	0	0	0	0	0	0	0	0	0	1	1

Table S11. System of linear equations written in matrix form for the calculation of LUMO energies in the ground state. *p* and *o* indicate the position of the substituents with respect to the hydroxyl group, respectively; all the other entries represent the substituents on the imidazopyridine moiety.
